# Open-Source Chromatographic Data Analysis for Reaction Optimization and Screening

**DOI:** 10.1021/acscentsci.2c01042

**Published:** 2023-02-09

**Authors:** Christian P. Haas, Maximilian Lübbesmeyer, Edward H. Jin, Matthew A. McDonald, Brent A. Koscher, Nicolas Guimond, Laura Di Rocco, Henning Kayser, Samuel Leweke, Sebastian Niedenführ, Rachel Nicholls, Emily Greeves, David M. Barber, Julius Hillenbrand, Giulio Volpin, Klavs F. Jensen

**Affiliations:** † Department of Chemical Engineering, Massachusetts Institute of Technology, 77 Massachusetts Avenue, Cambridge, Massachusetts 02139, United States; ‡ Research and Development, Small Molecules Technologies, Bayer AG, Crop Science Division, Industriepark Höchst, 65926 Frankfurt am Main, Germany; § Research and Development, Small Molecules Technologies, Bayer AG, Crop Science Division, Alfred-Nobel-Straße 50, 40789 Monheim am Rhein, Germany; ∥ Chemical & Pharmaceutical Development, Bayer AG, Pharmaceuticals Division, Müllerstraße 178, 13353 Berlin, Germany; ⊥ Applied Mathematics, Bayer AG, Enabling Functions Division, Kaiser-Wilhelm-Allee 1, 51368 Leverkusen, Germany; # Research and Development, Computational Life Science, Bayer AG, Crop Science Division, Alfred-Nobel-Straße 50, 40789 Monheim am Rhein, Germany; ¶ Research and Development, Weed Control Chemistry, Bayer AG, Crop Science Division, Industriepark Höchst, 65926 Frankfurt am Main, Germany; ■ Chemical & Pharmaceutical Development, Bayer AG, Pharmaceuticals Division, Friedrich-Ebert-Straße 475, 42117 Wuppertal, Germany

## Abstract

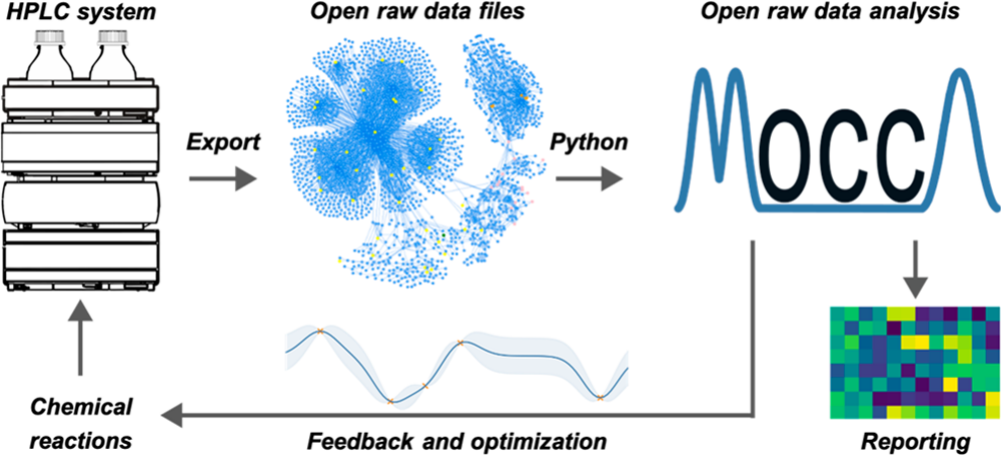

Automation and digitalization solutions in the field of small molecule synthesis face new challenges for chemical reaction analysis, especially in the field of high-performance liquid chromatography (HPLC). Chromatographic data remains locked in vendors’ hardware and software components, limiting their potential in automated workflows and data science applications. In this work, we present an open-source Python project called MOCCA for the analysis of HPLC–DAD (photodiode array detector) raw data. MOCCA provides a comprehensive set of data analysis features, including an automated peak deconvolution routine of known signals, even if overlapped with signals of unexpected impurities or side products. We highlight the broad applicability of MOCCA in four studies: (i) a simulation study to validate MOCCA’s data analysis features; (ii) a reaction kinetics study on a Knoevenagel condensation reaction demonstrating MOCCA’s peak deconvolution feature; (iii) a closed-loop optimization study for the alkylation of 2-pyridone without human control during data analysis; (iv) a well plate screening of categorical reaction parameters for a novel palladium-catalyzed cyanation of aryl halides employing *O*-protected cyanohydrins. By publishing MOCCA as a Python package with this work, we envision an open-source community project for chromatographic data analysis with the potential of further advancing its scope and capabilities.

## Abstract 2

MOCCA is an open-source Python project for HPLC−DAD raw data analysis in reaction optimization and screening enabling data-based decisions in automated synthetic chemistry.

## 1 Introduction

Synthetic chemistry enables discovery of new chemical reactivity, access to new molecules of interest, and development of corresponding chemical processes. Ever more demanding regulatory and sustainability requirements on small molecules’ synthesis and development make this endeavor complex and cost-intensive.^1−5^ Increasing emphasis is given to automation and digitalization in synthetic chemistry to address today’s complex challenges while decreasing development time and cost.^6−8^ Automation approaches aim to facilitate chemical synthesis while increasing its safety, robustness, and efficiency.^9−11^ Digitalization approaches focus mainly on reducing the number of synthetic experiments until a given goal is reached by predicting experimental outcomes or molecular properties. For that, data science techniques are applied to existing data for experimental design and decision making.^12−15^ In both areas, generalization to the complexity and diversity of chemical reaction processes remains the main challenge. As stated by Hein and co-workers,^16^ “automation isn’t automatic,” and automated experimental setups are too often tailored to a given synthetic problem.^17−19^ Digitalized approaches toward machine learning design algorithms suffer from a highly unstructured data foundation in literature, since data was not collected and reported with data science applications in mind.^20^ Therefore, recent approaches focus on the community-based standardization of synthetic lab data (Open Reaction Database), increasing the robustness of experimental protocols against noise, or the augmentation of literature data by systematic experiments performed by automated machines.^21−24^

Interestingly, chemical reaction analysis has received less attention in recent automation and digitalization efforts despite its importance for the overall synthetic process.^25^ Analytical raw data generation and analysis remain locked in vendor-specific proprietary hardware and software components, especially in the field of high-performance liquid chromatography (HPLC), a standard analytical method for chemical reaction analysis. Most HPLC systems in academic and industrial research laboratories are equipped with photodiode array detectors (DAD) that record full UV–Vis spectra at every chromatogram time point. For analysis, the dimensionality of the HPLC–DAD data is classically reduced to chromatograms by vendor data analysis software, i.e., absorbance at a single wavelength as a function of retention time. In such data analysis software, HPLC–DAD raw data are often only used by expert users to check for peak purity or to identify a compound by comparison of the UV–Vis spectrum with a reference spectrum. Most workflows access chromatogram analysis results by vendor software in the form of peak tables. In extreme cases, a full HPLC–DAD raw data array is recorded only to extract one value out of a peak table, e.g., the area of the product signal, while all of the other information is discarded. This is incongruous with modern data-centered automation and digitalization approaches.

Commercial software solutions from Virscidian (Analytical Studio^26^) or ACD/Labs (Katalyst D2D, Spectrus^27^) have already filled the gap of modern multivariate raw data analysis. However, as commercial products, they provide limited flexibility in workflow implementation. For example, Virscidian had to implement a construct called *expressions* in their software to allow the user to extract relevant information in a customizable and flexible manner. The analytical chemistry community is also adopting multivariate data analysis, but code availability is limited.^28−31^ For example, Arase et al. explored with the Shimadzu Corporation as an HPLC instrument vendor the potential of HPLC–DAD data in the context of peak deconvolution.^32^

In this work, we present the open-source Python project MOCCA (Multivariate Online Contextual Chromatographic Analysis), which enables the direct processing and analysis of HPLC–DAD raw data in Python, the de facto standard programming/scripting language for data science projects in chemistry.^33−36^ As a ready-to-use Python package, it is easily implemented into existing automated and nonautomated workflows. By making the Pythonic library of data analysis toolkits accessible, MOCCA enables its users to develop new and powerful data analysis features. Here, we present a peak deconvolution feature that allows for automated deconvolution and quantification of known signals which overlap with signals of unexpected impurities. This overcomes a common limitation of available commercial software: the requirement for manual control of the automatic integration routine to account for overlapping peaks. With this feature implemented, MOCCA could play a major role toward autonomous laboratories by providing open actionable analytics, i.e., enabling data-based decisions without human intervention or control by putting HPLC–DAD data in the correct context for analysis.^19^

Other open-source toolkits exist for chromatographic data analysis, e.g., HappyTools^37^ and Aston^38^ in Python or chromatographR^39^ in R. The authors of the Alsace package for R emphasized the potential of DAD data for metabolomics profiling.^40^ Notably, Jason Hein and co-workers recently developed a Python-based automated data processing routine and made the code available online.^41^ However, all these efforts do not make consistent use of the multidimensionality of the HPLC–DAD data or are developed with a specific use case in mind so that they are not ready-to-use for a synthetic chemist. We envision MOCCA to serve as a basis for a joint community effort toward an open multivariate analytical raw data analysis toolkit.

MOCCA serves as a plug-and-play module and is not restrained by a specific project scope. To highlight MOCCA’s general applicability and versatility for chemical reaction analysis, we introduce and investigate MOCCA’s data analysis features in four different case studies. First, the features are validated using a large set of simulated chromatograms including overlapping signals for a quantitative investigation of the peak deconvolution feature. Then, the potential of MOCCA and its peak deconvolution feature is highlighted in an experimental reaction kinetics study on a Knoevenagel condensation reaction. In the third study, MOCCA is employed in a closed-loop process optimization for the alkylation of 2-pyridone where the peak deconvolution feature keeps the optimization cycle running despite the signal of an unexpected side product overlaps with the product signal. Finally, a newly developed cyanation of aryl halides is presented and categorical reaction parameters are screened on a well plate with MOCCA tracking all known and unknown signals.

## 2 Methods

**Figure 1:**
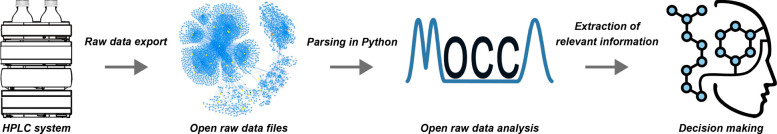
Proposed analytical workflow starting with HPLC systems controlled by vendor-specific software. HPLC–DAD raw data are exported in nonproprietary and open data formats, preferably, a metadata-enriched standardized format (Allotrope) implementing FAIR data principles. After parsing in Python, HPLC–DAD data sets are analyzed in context to each other by MOCCA. From the analysis results, structured data sets are generated for data-based decision making.

Our proposed analytical workflow employing the MOCCA package in automated, semiautomated, or nonautomated workflows is shown in Figure 1. In research laboratories, HPLC systems from a number of different vendors are used in combination with corresponding vendor-specific control software. The HPLC–DAD raw data (time–wavelength absorbance array) are typically stored in proprietary formats inaccessible to the user. To obtain open, nonproprietary HPLC–DAD raw data files, each of the softwares has its own native raw data export routine. Therefore, MOCCA includes raw data parsers for data exported from Agilent’s ChemStation, Shimadzu’s LabSolutions, and Water’s Empower software. However, we highly encourage, if possible, exporting to standardized and metadata-enriched data formats such as the Allotrope data format,^42^ for which a parser is implemented in the MOCCA package. These standardized data formats ensure the implementation of FAIR (findability, accessibility, interoperability, reuse) principles in analytical data and promote reuse of data for future scientific projects (details in SI section S2).^43^

**Figure 2:**
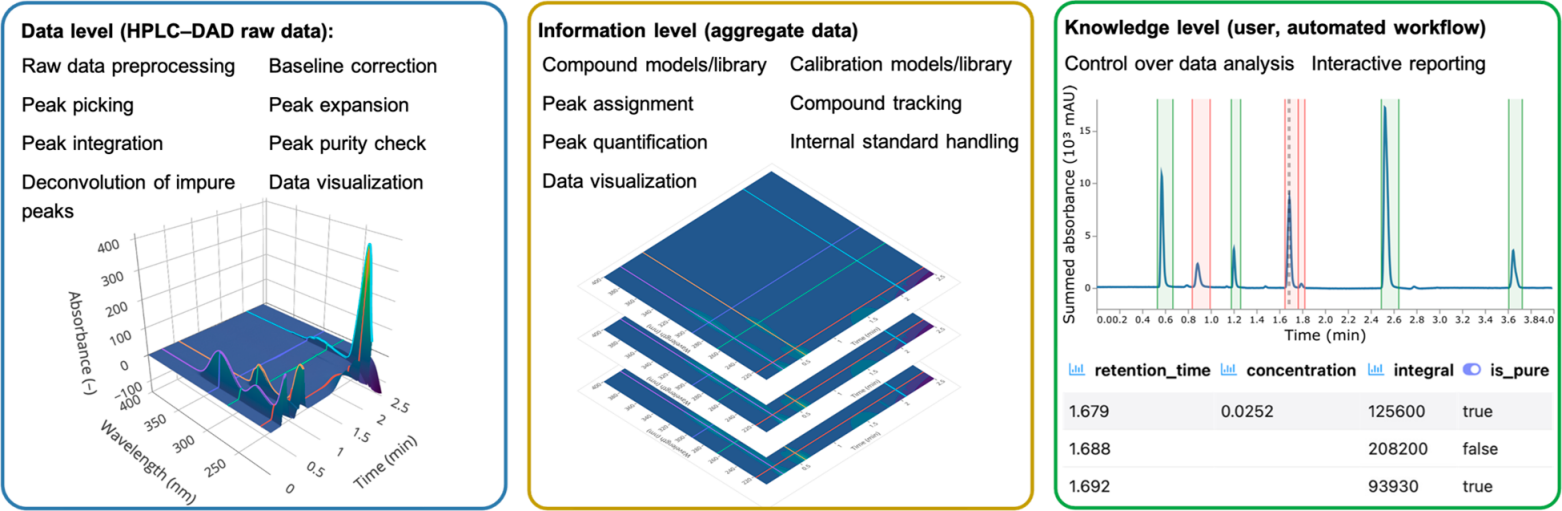
Summary of the data analysis features implemented in MOCCA.

A summary of the single data analysis features of the MOCCA package is presented in Figure 2 (details in SI sections S3 and S4). The features are assigned to three hierarchy levels: the raw data level, the aggregate data level, and the user or automated workflow interaction level. On the raw data level, most features are known from common vendor software and include raw data preprocessing with baseline correction as well as peak picking and integration. Other features like the algorithms for automated peak purity checking and peak deconvolution can complement vendor software capabilities. These two features are discussed and validated in detail in the following sections. On the aggregate data level, information is created by analyzing data sets in context to each other. By mimicking and automating routine steps a scientist would perform in the lab, compound and calibration libraries are created to allow for peak assignment and peak quantification. Moreover, MOCCA allows for the automated handling of internal standards for retention time correction as well as for relative quantification. Finally, interaction with the tool takes place on the highest hierarchy level, which provides control over certain settings of the data analysis and provides interactive reports on the analysis. The reports include the most crucial information for the user, such as chromatogram visualizations and peak tables (examples in chromatogram report in HTML SI files).

## 3 Results and Discussion

### 3.1 Validation of Data Analysis Features in Simulated Chromatograms

Collecting large-scale experimental HPLC–DAD data for validation is time-consuming and inefficient. Moreover, experimental data sets with overlapping signals do not provide a ground truth against which deconvolution results can be quantitatively compared. To solve this problem, we turned to the Chromatography Analysis and Design Toolkit (CADET), a tool that simulates retention processes on LC separation columns.^44^ With CADET, a wide variety of elution profiles can be simulated (including nongaussian shapes) while taking into account nonlinear retention effects of coeluting analytes.

To imitate a real situation and systematically explore the limitations of MOCCA’s peak deconvolution feature, chromatograms with two compounds (a known main compound and an unknown impurity) were generated in-silico. The resulting retention profiles were enriched with compound-specific UV–Vis spectra to obtain synthetic HPLC–DAD data sets. The similarity of UV–Vis spectra of the main compound and the impurity were varied in three levels of correlation coefficients *r*, high (*r* ≈ 0.86), medium (*r* ≈ 0.47), and low (*r* ≈ – 0.06). To obtain statistically relevant results, we simulated 1000 different chromatograms and enriched them with UV–Vis spectra of each similarity level resulting in 3000 HPLC–DAD data sets (details in SI section S7). These synthetic raw data were fed into MOCCA for data analysis. This allowed for the testing and validation of all other features shown in Figure 2. We provide the simulated data sets in the Supporting Information as a benchmark for future developments.

The obtained results were assigned to four possible categories: (i) separate peaks where the simulated pair of retention profiles is baseline-separated and the peak purity checker labels the two peaks as pure; (ii) successful deconvolution of an overlapping signal where the main compound is correctly assigned and quantified; (iii) unsuccessful deconvolution where the peak is labeled as impure but the deconvolution feature is not able to assign any deconvoluted component to the main compound; (iv) no trigger of the peak deconvolution feature due to the peak purity check returning a false positive result. In general, cases (i) and (ii) are considered as desired outcomes, while cases (iii) and (iv) are considered as misinterpretations (examples in SI section S7). Table 1 summarizes the obtained results highlighting that a vast majority of the simulated cases were processed correctly while almost all of the failing cases are attributed to category (iv).

**Table 1:** Results of the MOCCA Analysis of Synthetic HPLC–DAD Data Assigned to the Following Categories: (i) Retention Profiles of Main Compound and Impurity Were Baseline-Separated and Analyzed Correctly; (ii) Overlapping Retention Profiles Where the Peak Deconvolution Feature Was Triggered and the Main Compound Was Identified and Quantified; (iii) Peak Deconvolution Feature Was Triggered for the Overlapping Signal but the Main Compound Could Not Be Identified; (iv) the Signals Were Overlapping but Were Not Labelled As Impure by the Peak Purity Checker

	UV–Vis spectral similarity		
Result category	High	Medium	Low
(i)	86	86	86
(ii)	794	868	890
(iii)	2	0	0
(iv)	118	46	24

Cases of the category (iv) are not attributed to a failure of the peak deconvolution feature, but rather to a permissive peak purity checker returning false positive outcomes on strongly coeluting signals. These cases cannot be solved analytically, and the only solution would be the development of an HPLC method with higher chromatographic resolution to separate (at least partially) the elution profiles. MOCCA enables a shift toward shorter gradient times and faster sample processing, but the category (iv) failure rate shows that the user is still required to have expertise in HPLC method development to balance method time vs chromatographic resolution.^45^

For a quantitative investigation of the devonvolution results, we looked at the results assigned to category (ii) and compared them to the ground truth. For all three levels of spectral similarity, the median quantification error was smaller than 2%, while the third quartile error ranged around 6% (examples and details in SI section S7). The results obtained validate that MOCCA’s deconvolution feature works robustly enough for typical lab screenings, but should be treated with caution for regulated environments and process development scenarios where lower margins of error are required.

### 3.2 Kinetics Study of Knoevenagel Condensation Reactions

**Scheme 1:**
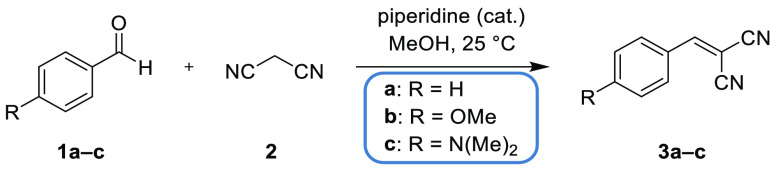
Knoevenagel Condensation Reactions of Benzaldehyde (**1a**), 4-Methoxybenzaldehyde (**1b**) and 4-(Dimethylamino)benzaldehyde (**1c**) with Malononitrile (**2**) in Methanol (MeOH) to Yield Benzylidenemalononitriles **3a**–**c**

A reaction kinetics study of a Knoevenagel condensation, a well-established test reaction,^46,47^ was conducted to highlight the potential of MOCCA’s peak deconvolution feature. Benzaldehydes (**1a**–**c**) were simultaneously reacted with malononitrile (**2**) to their corresponding benzylidenemalonitriles (**3a**–**c**) in the same reaction mixture (Scheme 1).

Reactions were performed in an HPLC vial in a temperature-controlled (25 °C) autosampler, and reaction progress was followed via reversed-phase HPLC with different gradient lengths. Five different HPLC methods were developed with gradient lengths of 0.5, 0.75, 1.0, 1.5, and 2.5 min (water/acetonitrile 95:5 → 0:100 v/v) to induce different degrees of overlap between the substrate signals. For quantification, calibration curves were recorded for all substrates with all HPLC methods. These measurements were used to validate the quantification features of MOCCA in the case of pure and baseline-separated signals against traditional manual data analysis. The results of both analysis methods correlated very precisely (details in SI section S5).

**Figure 3:**
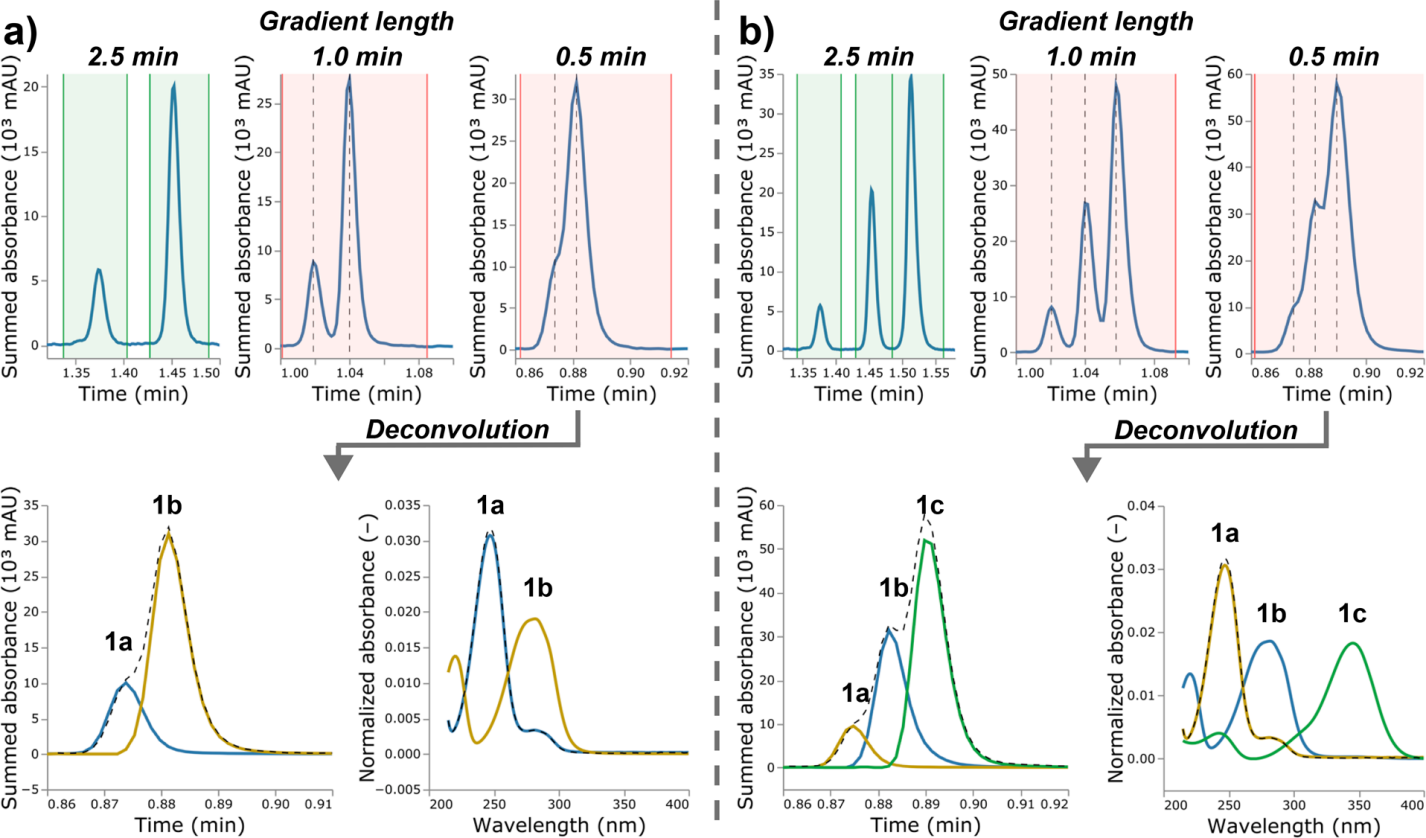
(a) Results of the competition experiment with two benzaldehydes (**1a** and **1b**). (b) Results of the competition experiment with three benzaldehydes (**1a**–**c**). *Top*: Chromatographic signals of the benzaldehydes using different gradient lengths. MOCCA indicates results of purity checks (green passed, red failed) and centers of retention profiles modeled by the deconvolution algorithm (vertical black dashed lines). *Bottom*: Deconvolution results of the overlapping signal recorded with a gradient length of 0.5 min. The modeled retention profiles (left, colored lines) described the retention profile of the impure peak (black dashed line). The modeled UV–Vis traces (right, colored lines) correspond to the UV–Vis spectra of the benzaldehydes as exemplified for **1a** (black dashed line).

Two competition experiments were performed: malononitrile (**2**) was reacted with two (**1a**, **1b**), and with three (**1a**–**c**) benzaldehyde substrates, respectively. For data analysis, benzaldehyde (**1a**) was treated as the main compound, i.e., only its calibration runs were added to MOCCA for quantitative analysis while the functionalized benzaldehydes **1b** and **1c** were treated as “unknown” impurities. Figure 3a and b illustrate results from the two competition experiments. The top panels show the different degrees of signal overlap induced by the gradient variation. Here, the peak purity check feature correctly labeled the peaks as pure for the long gradient (green background area) and correctly labeled the overlapping peaks as impure for the short gradients (red background area). In the latter cases, the peak deconvolution feature was triggered. As a first step of the deconvolution routine, a principal component analysis is performed on the absorbance array of an impure peak to estimate the number of overlapping components. With this number as an input, a newly developed iterative algorithm using parallel factor analysis (PARAFAC)^48^ is employed for deconvolution (details in SI section S4). The bottom panels in Figure 3 show the deconvolution results for the peaks recorded with a gradient length of 0.5 min.

**Figure 4:**
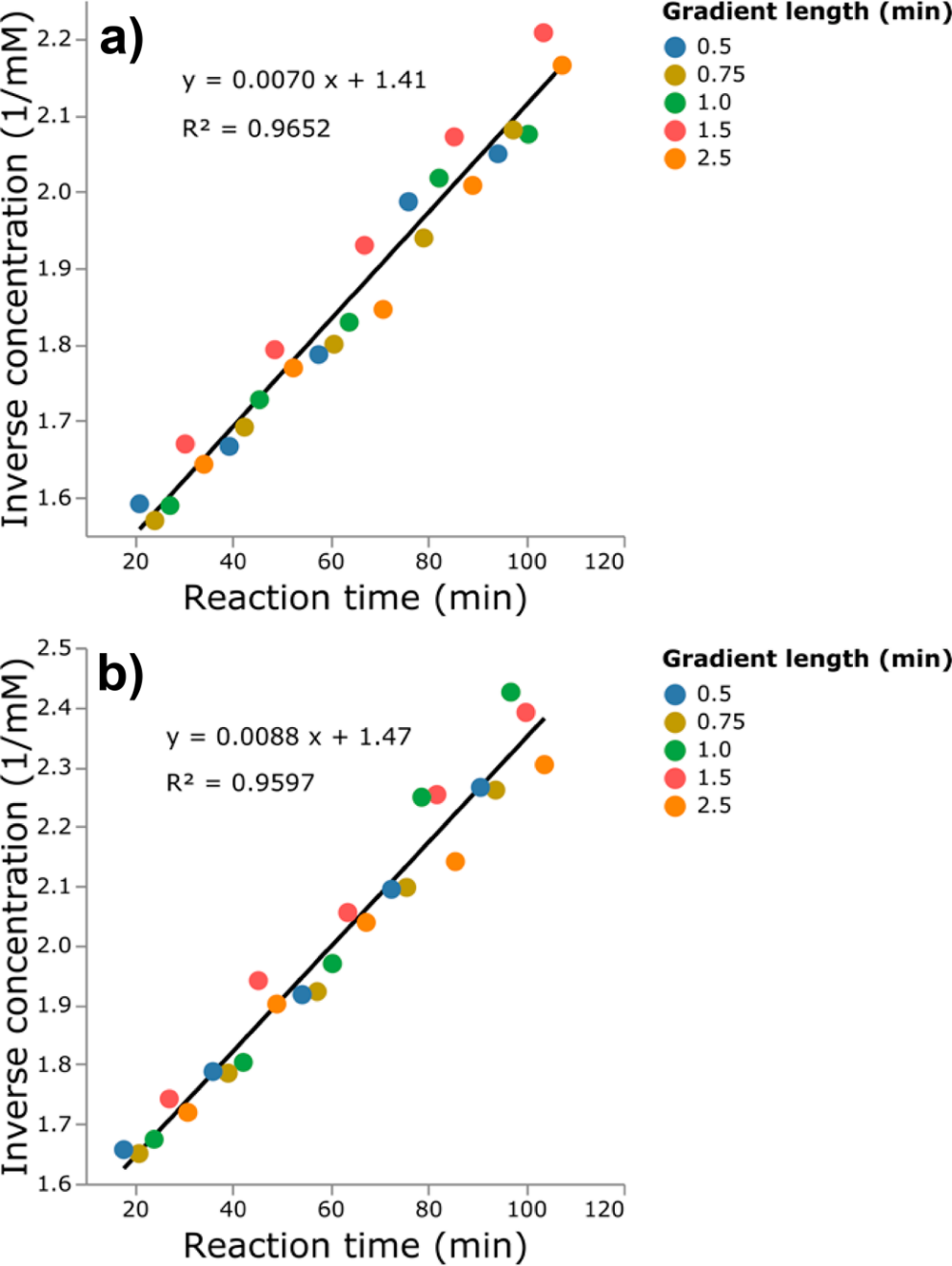
Second-order reaction kinetics plots of benzaldehyde (**1a**) in the Knoevenagel condensation recorded with five different HPLC methods employing varying gradient lengths. (a) Competition experiment with two benzaldehydes (**1a** and **1b**). (b) Competition experiment with with three benzaldehydes (**1a**–**c**).

To investigate the ability of MOCCA to automatically recognize impure peaks and decompose a known signal from coeluting impurities, reaction progress was followed by sampling out of the same reaction vessel repeating each of the five HPLC methods iteratively (details in SI section S6). The resulting reaction kinetics plots of the main compound benzaldehyde (**1a**) are shown in Figure 4 and exhibit the expected second-order kinetics.^46,49^ As expected from the simulation study, the results of chromatograms with baseline-separated signals agree with the results of chromatograms where signals were heavily overlapping. The deconvolution feature successfully identified the benzaldehyde (**1a**) signal in all given impure peaks and returned modeled peaks for quantification.

### 3.3 Closed-Loop Optimization of the Alkylation of 2-Pyridone

**Figure 5:**
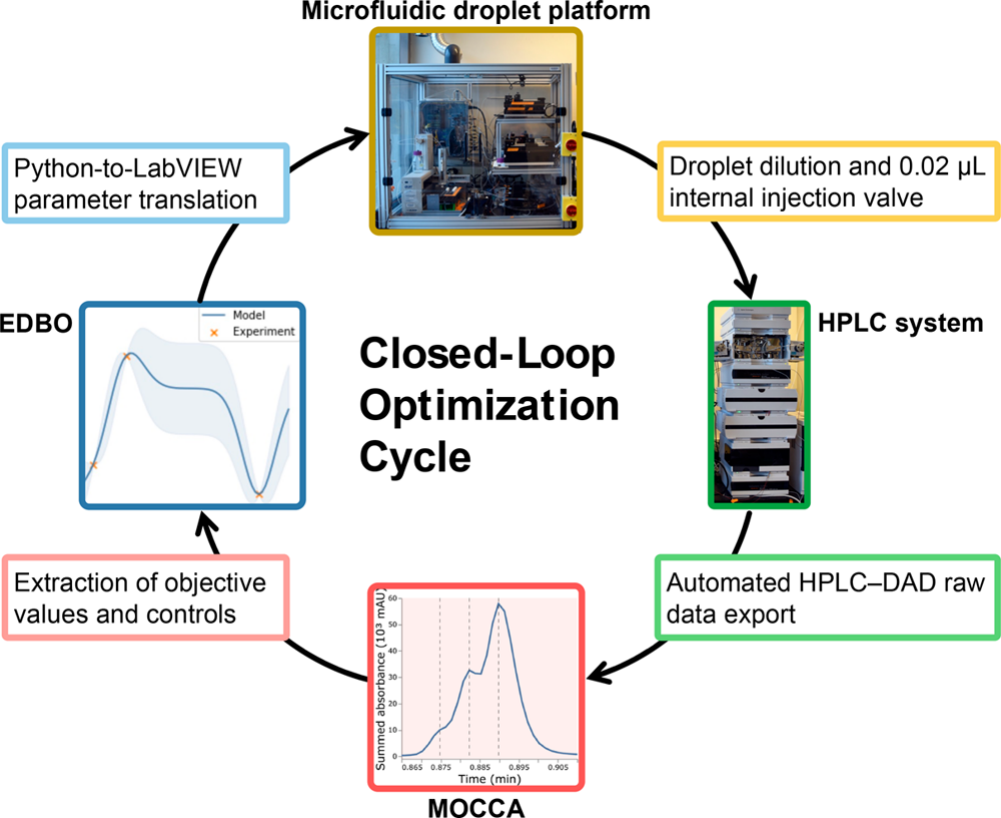
Closed-loop optimization cycle employed in this work. *Blue*: Experimental Design via Bayesian Optimization (EDBO) Python package from the Doyle group^55^ and translation of the suggested parameters to a LabVIEW experimental protocol. *Yellow*: Experimental execution by a microfluidic reactor platform employing an oscillatory droplet reactor design. 0.02 μL HPLC samples are taken out of the droplet after diltution with acetonitrile. *Green*: HPLC system with a photodiode array detector (DAD) and an automated HPLC–DAD raw data export routine. *Red*: Data analysis by the MOCCA tool and a project-specific script for the extraction of objective values and process control values.

Closed-loop optimization studies have gained tremendous attention in recent years due to their relevance for chemical discovery as well as process optimization.^50−54^ In such closed-loop processes, the optimization platform runs without human intervention and control of HPLC data analysis. Here, the peak purity check and peak deconvolution feature of MOCCA are of particular interest. Overlapping peaks or inaccurate integration routines (examples in SI section S5) lead to wrong analytical results fed back to the experimental design algorithm. The setup of the closed-loop optimization platform of this study is shown in Figure 5. For the experimental design, we employed a Python package called Experimental Design via Bayesian Optimization (EDBO) published by Doyle and co-workers.^55^ The suggested optimization parameter values were fed into a LabVIEW program controlling a microfluidic droplet platform, which was developed in the Jensen group for the simultaneous screening of both categorical and continuous reaction parameters.^56−60^ After reaction completion in an oscillatory droplet reactor, the droplet was diluted with acetonitrile and moved to an internal injection valve (0.02 μL injection volume) to inject a sample directly on a reversed-phase separation column of the HPLC system. The HPLC system automatically exported HPLC–DAD raw data for MOCCA data analysis after each run. The optimization objective, as well as process control parameters were extracted from the MOCCA analysis results via a project-specific script.

**Scheme 2:**
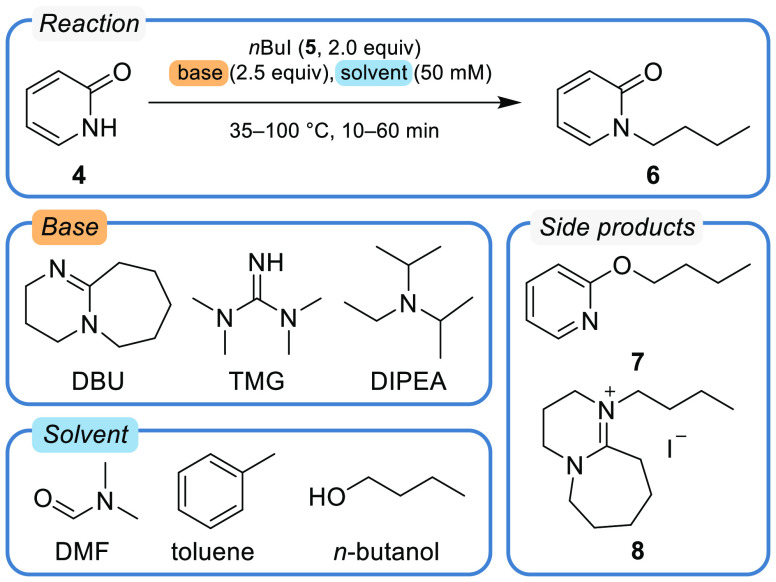
Optimization Campaign on the Alkylation of 2-Pyridone (**4**) with 1-Iodobutane (**5**) Yielding 1-Butylpyridone (**6**) The domain space of the optimization campaign spans over two continuous variables, reaction time (low boundary: 10 min, high boundary: 60 min), and temperature (low boundary: 35 °C, high boundary: 100 °C), as well as two categorical variables, identity of base (DBU, TMG, DIPEA) and solvent (DMF, toluene, *n*-butanol). The objective value of the optimization is the yield of **6**. Two side products were identified with 2-butoxypyridine (**7**) and butylated DBU (**8**).

As a test reaction, we examined the alkylation of 2-pyridone (**4**) using 1-iodobutane (**5**) to yield the two regioisomers 1-butylpyridone (**6**) and 2-butoxypyridine (**7**). As shown in Scheme 2, the optimization was performed on two continuous variables, the reaction time (10–60 min) and temperature (35–100 °C). Additionally, two categorical optimization parameters were screened: the base (1,8-diazabicyclo[5.4.0]undec-7-ene (DBU), 1,1,3,3-tetramethylguanidine (TMG), *N*,*N*-diisopropylethylamine (DIPEA)), and the solvent (*n*-butanol, *N*,*N*-dimethylformamide (DMF), toluene). The maximization of the yield of 1-butylpyridone (**6**) served as the objective function for the optimization. For quantification, the desired product **6** was calibrated relative to an internal standard in an automatic fashion by the platform (details in SI section S8).

**Figure 6:**
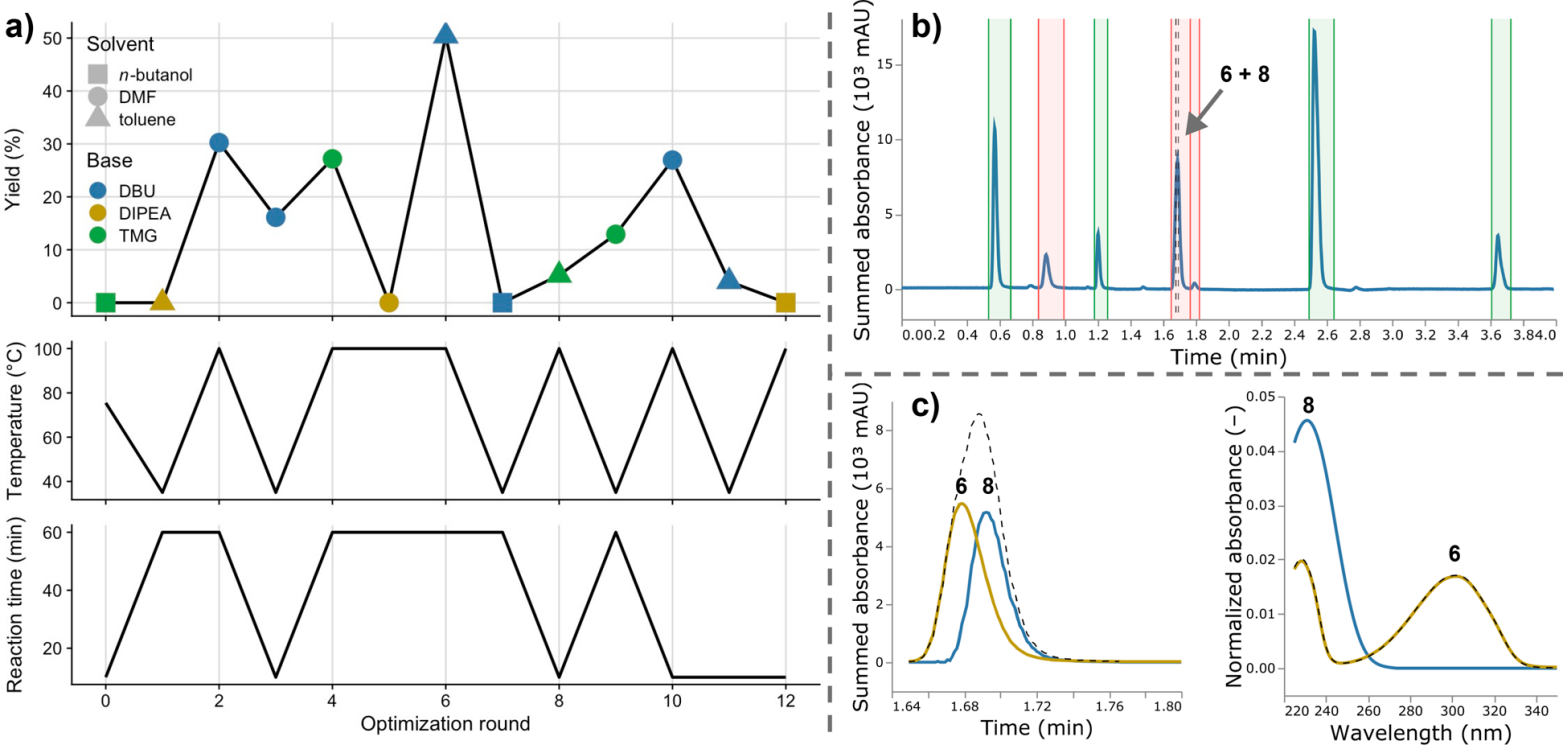
Results of the closed-loop optimization on the alkylation of 2-pyridone (**4**). (a) Objective values as a function of optimizer choices in each round. *Top*: Objective value (yield of **6**) with marker shape indicating the chosen solvent and marker color indicating the chosen base; *middle*: Chosen reaction temperature; *bottom*: Chosen reaction time. (b) Chromatogram of the reaction under optimal conditions with an impure product peak (∼1.7 min). (c) Modeled retention profiles (dashed line: impure peak) and UV–Vis spectra (dashed line: reference UV–Vis spectrum of (**6**) of the product **6** (yellow) and the unexpected impurity **8** (blue).

The optimization cycle was run with a batch size of one, i.e., the feedback loop was closed after each experiment. At any point during the optimization campaign, the user was able to extract MOCCA reports to follow the optimization process. Figure 6a summarizes the results of the optimization campaign. The optimal conditions found for the reaction were DBU in toluene for 60 min at 100 °C. We validated the obtained optimization results with batch reactions that screened all categorical parameter combinations at 35 and 100 °C (details in SI section S8). For all reactions with DBU, the HPLC signal of an unexpected side product, butylated DBU (**8**), started overlapping with the signal of the calibrated product **6**, whose yield served as the objective value for the optimization. Figure 6b shows the chromatogram of the reaction at optimal conditions with the impure peak at ∼1.7 min resulting from an overlap of signals from **6** and **8**. As shown in Figure 6c, MOCCA was able to deconvolute this impure peak in an automated fashion and to feed back corrected yields to the design algorithm EDBO. This highlights MOCCA’s ability to keep closed-loop cycles running even when unexpected coelution of calibrated signals occurs in the HPLC analysis.

### 3.4 Palladium-Catalyzed Cyanation of Aryl Halides

With this study, we highlight MOCCA’s application for the analysis of HPLC–DAD data originating from a novel palladium-catalyzed cyanation of aryl halides, where side products were unknown. Palladium-catalyzed cyanation reactions are a prominent and well-investigated reaction class.^61−63^ They proceed via oxidative addition of an aryl halide to a Pd(0)/ligand complex, subsequent halide/cyanide exchange, followed by a reductive elimination which closes the catalytic cycle.^64^ A particular challenge with this reaction class resides in the rapid deactivation of the catalytically active palladium species in the presence of excess amounts of cyanide.^65,66^ To overcome this issue, many procedures were developed with the aim of keeping a low effective concentration of cyanide in solution. Common strategies include the use of hardly soluble metal salts,^67−69^ employing cyanide transfer agents,^70^ and the slow addition of trimethylsilyl cyanide^71^ or acetone cyanohydrin.^72,73^ The use of butyronitrile in combination with a nickel catalyst allows for cyanide release through a reverse hydrocyanation reaction.^74^

**Scheme 3:**
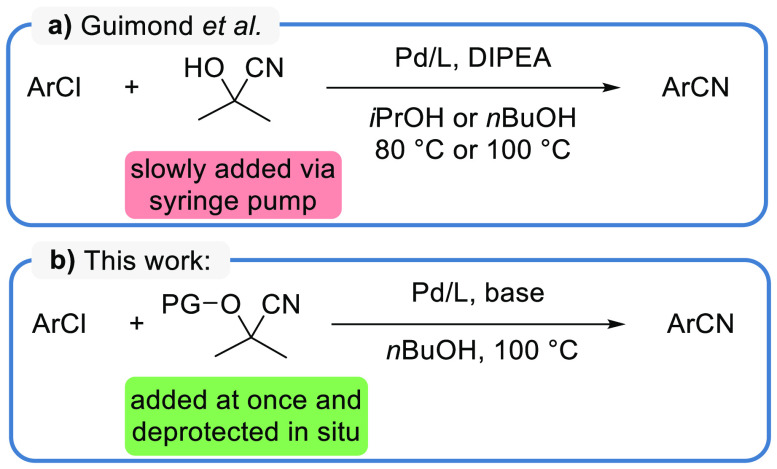
(a) Palladium-Catalyzed Cyanation of Aryl Chlorides Developed by Guimond et al. Based on the Slow Addition of Acetone Cyanohydrin via Syringe Pump.^73^ (b) Newly Developed Cyanation Method Using Protected Cyanohydrins (PG: protecting group) for in Situ Release of Cyanide

Giumond et al.^73^ developed a protocol for palladium and nickel catalyzed cyanation reactions to overcome upscaling issues associated with the use of metal cyanides under heterogeneous conditions.^68,75−77^ A homogeneous reaction is obtained by adding acetone cyanohydrin via syringe pump to a solution of the substrate, a palladium catalyst, a ligand, and *N*,*N*-diisopropylethylamine (DIPEA) in isopropyl alcohol or *n*-butanol (Scheme 3a).^73^ Based on these results, we envisioned to make use of *O*-protected cyanohydrins as cyanation reagents which release cyanide in situ upon deprotection (Scheme 3b). This approach maintains a fully homogeneous liquid system but the need for slow reagent addition is avoided.

**Figure 7:**
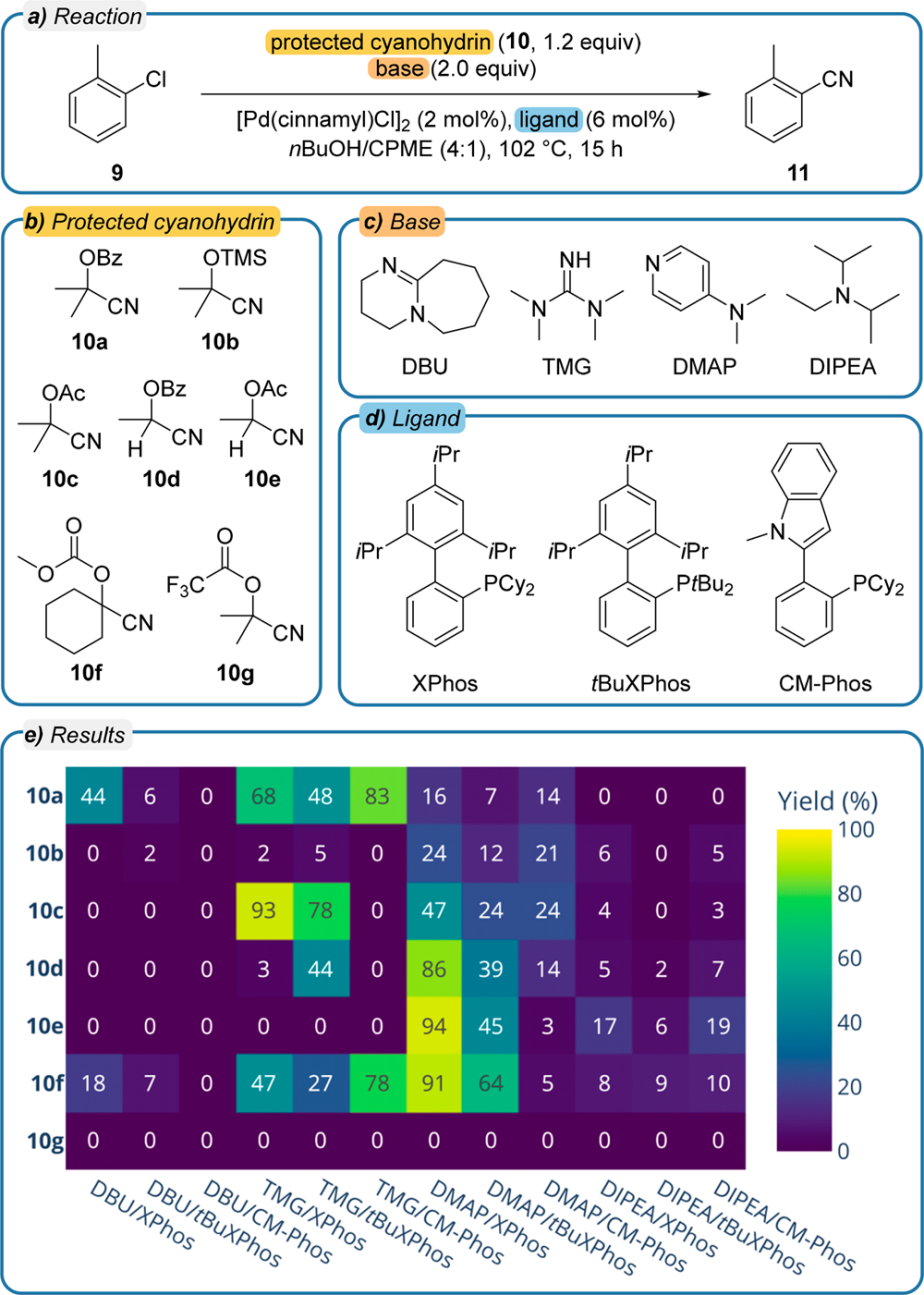
(a) Reaction conditions for the well plate screening of the cyanation of 2-chlorotoluene (**9**) yielding *o*-tolunitrile (**11**) using palladium(π-cinnamyl) chloride dimer as the catalyst precursor. (b) Screened *O*-protected cyanohydrins. (c) Screened bases. (d) Screened ligands. (e) Yield of *o*-tolunitrile (**11**) in dependency on the employed protected cyanide-releasing agent **10** as well as the chosen ligand and base. CPME: cyclopentyl methyl ether; Bz: benzoyl group; Ac: acetyl group; TMS: trimethylsilyl group.

To investigate our proposed synthetic strategy, we prepared a number of protected cyanide-releasing agents **10a**–**10g** (Figure 7b). In situ deprotection by transesterification or TMS cleavage yields acetone cyanohydrin or lactonitrile which rapidly eliminate the cyanide required for cross-coupling. We screened suitable reaction conditions for the conversion of 2-chlorotoluene (**9**) to *o*-tolunitrile (**11**) in a 96 well plate (Figure 7a) by combining these reagents with one of three different ligands (Figure 7d), XPhos, *t*BuXPhos, or CM-Phos, and one of four different bases (Figure 7c), DBU, TMG, 4-(dimethylamino)pyridine (DMAP), or DIPEA (details in SI section S9). The choice of ligands and [Pd(cinnamyl)Cl]_2_ as the catalyst precursor was based on previous literature reports.^73,78−80^

After the reaction was run under the given conditions and subsequent internal standard addition, samples of the reactions were subjected to HPLC analysis. The HPLC–DAD raw data were exported as text files and parsed for subsequent MOCCA analysis, which took ∼10 min on a standard personal laptop for the whole data set including 42 deconvolutions of impure peaks. The MOCCA analysis results enabled following product and substrate concentrations and, importantly, unknown signals over the data sets, thus supporting the identification of side products and impurities (example in SI section S9). These data were used for heatmap visualization in Python using standard toolkits (Plotly^81^). For example, the obtained yields of *o*-tolunitrile (**11**) are visualized based on their location in the well plate (Figure 7e). This again highlights the potential of moving HPLC–DAD data analysis to Python with its powerful package library for data analysis and visualization.

As discussed above, a successful reaction requires the release of cyanide anions to proceed at a rate that is sufficient to be productive but not outpace the catalyst turnover. This rate is controlled through the rates of deprotection of the cyanide-releasing agents **10**, which were examined experimentally for a better understanding of our results (details in SI section S9). The screening provided three parameter combinations with yields >90% indicating a good harmonization between cyanide release and turnover, the TMG/XPhos base–ligand combination with the precursor **10c**, DMAP/XPhos with **10e**, and DMAP/XPhos with **10f**. The outcome of these experiments together with a selection of the other experiments were verified by repeating the reactions in standard reaction flasks (details in SI section S9). For other parameter combinations, e.g., when using trifluoroacetylated cyanohydrin **10g**, the release of cyanide is too fast, leading to a quick catalyst deactivation. In contrast, a slow release of cyanide is observed with the use of DIPEA, a weak base, leading to low conversions (detailed mechanistic discussion in SI section S9).

HPLC analysis represents a typical bottleneck in well plate-based screenings. Typically, HPLC methods are developed to be as short as possible for maximum throughput while resolving all known compounds. When screening categorical variables like ligands or bases, unexpected side products often overlap with known signals in the chromatogram. This also happened in the described screening campaign, but MOCCA reliably deconvoluted these overlapping peaks and enabled an efficient data analysis without the need for HPLC method optimization or resorting to multiplexing techniques (examples and details in SI section S9).^82,83^

## 4 Conclusions

In this work, we have presented MOCCA, an open-source Python project, for the comprehensive analysis of HPLC–DAD raw data. Compared to typical data analysis methodologies on one signal wavelength, the analysis of the full time–wavelength absorbance array gives multiple advantages. These include robust peak assignment and quantification, as well as peak purity checks and the deconvolution of overlapping peaks. We investigated MOCCA in four case studies, (i) a simulation study, (ii) a reaction kinetics study, (iii) a closed-loop optimization, (iv) a well plate screening and demonstrated MOCCA’s broad applicability and the benefit of moving chromatographic data analysis to an open environment like Python.

In this spirit, we envision MOCCA becoming a community project with a significant user base eager to adapt, curate, and further advance the tool. With community support, MOCCA can overcome limitations of vendor software especially with regard to FAIR data principles and implementation in automated workflows. The development of additional data analysis features such as the implementation of a mass spectrometry module could extend the scope of the tool by adding orthogonal analysis dimensions. Another interesting development could be a connection MOCCA to chemical structure representations, or even to chemical reaction entries in electronic lab notebooks. This would make synthetic chemistry data and the corresponding analytical data directly accessible for machine learning in data science applications.

To enable new users to implement MOCCA easily in their laboratories, we packaged MOCCA and published it in the Python Package Index (PyPI). For a quick start, example JupyterLab notebooks together with the corresponding HPLC–DAD data sets are provided in the notebooks folder of the package’s GitHub repository.^84^ This includes a tutorial as well as the complete data analysis of the well plate screening presented in this manuscript.

## Data Availability

The full Python base code is available through GitHub at https://github.com/HaasCP/mocca. The MOCCA package is available on the PyPI server (https://pypi.org/project/mocca/) and MOCCA can be installed following the documentation at https://mocca.readthedocs.io/en/latest/readme.html. Simulated HPLC–DAD data sets saved as MOCCA campaigns, which were used for validation and benchmarking, are available at 10.5281/zenodo.7406829.

## Supporting Information Available

Additional details to all presented case studies, description how to extract HPLC–DAD raw data from vendor control software of major vendors, technical details to MOCCA’s data analysis features, NMR spectra of *O*-protected cyanohydrins (PDF)
MOCCA reports for the data analysis of the well plate screening (cyanation of aryl halides) (ZIP)
