# Supplementary material for: Open-Source Chromatographic Data Analysis for Reaction Optimization and Screening
Source: ACS Cent Sci. 2023 Feb 9;9(2):307–17. doi: 10.1021/acscentsci.2c01042 (PMC9951288; doi:10.1021/acscentsci.2c01042)
Supplement: Supplementary file 1 — oc2c01042_si_001.pdf [file oc2c01042_si_001.pdf]

Supplementary Information

Open-Source Chromatographic Data Analysis for Reaction Optimization and Screening

Christian P. Haas,[a,b] Maximilian Lübbesmeyer,[a,b] Edward H. Jin,[a] Matthew A. McDonald,[a] Brent A. Koscher,[a] Nicolas Guimond,[c] Laura Di Rocco,[d] Henning Kayser,[c] Samuel Leweke,[e] Sebastian Niedenführ,[f] Rachel Nicholls,[f] Emily Greeves,[b] David M. Barber,[g] Julius Hillenbrand,*[h] Giulio Volpin,*[b] and Klavs F. Jensen*[a]

*Correspondence: julius.hillenbrand@bayer.com (Julius Hillenbrand), giulio.volpin@bayer.com (Giulio Volpin), kfjensen@mit.edu (Klavs F. Jensen)

[a] Department of Chemical Engineering
Massachusetts Institute of Technology, 77 Massachusetts Avenue, Cambridge, Massachusetts 02139, United States.

[b] Research and Development, Small Molecules Technologies
Bayer AG, Crop Science Division, Industriepark Höchst, 65926 Frankfurt am Main, Germany.

[c] Research and Development, Small Molecules Technologies
Bayer AG, Crop Science Division, Alfred-Nobel-Straße 50, 40789 Monheim am Rhein, Germany.

[d] Chemical & Pharmaceutical Development
Bayer AG, Pharmaceuticals Division, Müllerstraße 178, 13353 Berlin, Germany.

[e] Applied Mathematics
Bayer AG, Enabling Functions Division, Kaiser-Wilhelm-Allee 1, 51368 Leverkusen, Germany.

[f] Research and Development, Computational Life Science
Bayer AG, Crop Science Division, Alfred-Nobel-Straße 50, 40789 Monheim am Rhein, Germany.

[g] Research and Development, Weed Control Chemistry
Bayer AG, Crop Science Division, Industriepark Höchst, 65926 Frankfurt am Main, Germany.

[h] Chemical & Pharmaceutical Development
Bayer AG, Pharmaceuticals Division, Friedrich-Ebert-Straße 475, 42117 Wuppertal, Germany.

S1) General experimental information 3

S2) How to export HPLC–DAD raw data files from HPLC control software 4

S3) HPLC best practices using MOCCA 7

S4) Technical description of implemented data analysis features 9

S5) Validation of quantification feature 23

S6) Kinetics study of Knoevenagel condensation reactions 26

S7) Validation of data analysis features in simulated chromatograms 28

S8) Closed-loop optimization of the alkylation of 2-pyridone 37

S9) Palladium-catalyzed cyanation of aryl halides 43

S10) NMR spectra *O*-protected cyanohydrins 58

S1) General experimental information

If not stated differently, all chemicals were purchased from commercial suppliers and were used as received. Anhydrous solvents were bought in sealed bottles and withdrawn from these using common Schlenk technique.

^{1}H NMR and ^{13}C NMR spectra were recorded on a Bruker Avance-III HD Nanobay spectrometer operating at 400.09 MHz, which was equipped with a 5 mm liquid-nitrogen cooled Prodigy broad band observe BBO cryoprobe, a Bruker Avance Neo spectrometer operating at 400.17 MHz, which was equipped with a 5 mm BBFO SmartProbe, or a Bruker Avance Neo spectrometer operating at 500.34 MHz, which was equipped with a 5 mm liquid-nitrogen cooled Prodigy BBO cryoprobe. The spectra were referenced to the residual monoprotic solvent peak: $CDCl_3$: δ = 7.26 ppm for ^{1}H NMR and δ = 77.16 ppm for ^{13}C NMR; DMSO-d6: δ = 2.50 ppm for ^{1}H NMR and δ = 39.52 ppm for ^{13}C NMR. Coupling constants (*J*) are specified in brackets. ^{1}H NMR multiplicities are described using the following abbreviations: s = singlet, d = doublet, t = triplet, q = quartet, m = multiplet.

Further study-specific information will be given in the sections below.

S2) How to export HPLC–DAD raw data files from HPLC control software

To highlight MOCCA's ability to standardize data analysis over a range of different vendor software, we developed parsers for the exported raw data formats from three commonly used HPLC systems running corresponding control software, i.e., Agilent LC systems using OpenLab CDS ChemStation Edition (or ChemStation) software, Waters LC systems using Empower software and Shimadzu LC systems using LabSolutions software. Moreover, we developed a parser for data in the Allotrope Data Format (adf), a standardized scientific data format developed by a pre-competitive industrial consortium that enables the storage of HPLC–DAD raw data together with its metadata.[1] Finally, MOCCA allows the input of data which are already accessible in the memory of a Python environment. This allows to analyze data which are, for example, obtained by simulation of chromatographic processes. Since most HPLC vendors make it difficult to export raw data out of the HPLC intrument control softwares, this process is described in the following for each of the mentioned vendors. In future, parsers for more HPLC systems can be added if required. Alternatively, existing parsing frameworks like Entab[2] could be implemented in MOCCA.

Agilent ChemStation

For the export of HPLC–DAD raw data out of OpenLab CDS ChemStation Edition, a post-method macro is used which is triggered after each HPLC run. On one of ChemStation's installation CDs, a number of different macros are collected, which were developed and maintained by Agilent, including the macro named "Export3D.mac". Documentation on the installation procedure of the macro as well as on how to employ the macro both manually and automatically is presented in the accompanying readme file. More information on macro development can be found in the HP ChemStation Macro Programming Guide.[3] We slightly modified the Export3D macro to export values with less decimal places than in its original form to decrease the post-method export time as well as the size of the resulting csv file.

Waters Empower

Unfortunately, we could not find an automated way of exporting HPLC–DAD raw data out of Waters Empower software. Instead, a manual way of exporting the data as ASCII files is possible (file extension .arw). Documentation on that process can be found in the Waters Knowledgebase.[4]

Shimadzu LabSolutions

HPLC-DAD data can be automatically exported as a text file (.txt) during batch operation in Shimadzu LabSolutions. The export option should be selected in the "ASCII Conversion" pane of the batch file (.lcb) settings and the desired data are checked; exportable data include raw HPLC-DAD data, peak tables, single wavelength chromatograms, and others. The text files are generated into the same folder as LabSolutions data files (.lcd) and are either overwritten on each run or auto incremented with an appended counter (e.g. SampleData001.txt). HPLC-DAD raw data sampling rate and sampling wavelengths can be adjusted in the LabSolutions method files (.lcm) to optimize the resulting text file output.

Allotrope Data Format

The Allotrope Data Format (ADF) is one physical representation (amongst others as the JSON-based Allotrope Simple Model) of an industry-agnostic data standard for analytical and scientific data. ADF addresses data integrity, compliance and access needs and is developed and maintained by an active Pharma/BioTech-led industry consortium,[5] the "Allotrope Foundation (AF). In the ADF, the Allotrope Data Model (ADM) is built on a binary HDF5 container as an efficient and truncable data storage.[6] The HPLC ADM provides a "data description layer", which is a triple store for semantic meta data annotations.[7] For MOCCA, we target the "data cube layer" of the HPLC ADM which stores chromatographic raw data (up to TB scale), including multi-dimensional HPLC–DAD data. From the employed HPLC instruments in this study, only Agilent offered the possibility to export raw HPLC–DAD data in the ADF.[8] ADF export capabilities exist for other major chromatography data systems as well, but are currently feature-limited to single wavelength channel exports.[9–11]

To parse ADF files in Python, we employed the h5py Python package to read the data cube layer containing the HPLC–DAD raw data.[12] With the h5ld package, the data

description layer could be read as an rdflib graph object and Allotrope ontology terms were used to query the semantic triples.

In a future development, MOCCA could be extended with a module which allows to write data analysis results in ADF files. Moreover, the standardization of chromatographic data formats using ADF entails the idea of standardizing data analysis as well, e.g., by using open-source tools such as MOCCA.

S3) HPLC best practices using MOCCA

MOCCA is not designed to solve all analysis tasks and problems on the side of an automated data analysis tool. Instead, the user is required to have adequate expertise in HPLC techniques. Recording the correct HPLC runs allows avoiding some major obstacles in the analysis of HPLC–DAD data. This hybrid (experimental–computational) design of MOCCA requires the user to follow certain rules for the HPLC runs in the lab to enable the tool's full potential in computer-based data analysis.

In general, MOCCA is only capable of analyzing datasets in context to each other, if all datasets have been recorded on the same HPLC system, with the same HPLC column, using the same HPLC method. It cannot account for systematic drifts induced by a change of the setup, such as changes in the system's dead volume, the change to another HPLC column, or the change of the HPLC gradient. If not done properly, even the exchange of the mobile phase can lead to such disturbing drifts. Hence, filling up eluent bottles before starting long sequences of HPLC runs is recommended as the HPLC runs should be analyzed with MOCCA in context with each other.

All HPLC runs planned to be analyzed by MOCCA should be performed in a reproducible fashion to avoid analysis errors. This means that the user should follow typical HPLC best practices.[13] Most importantly, it is not recommended to record analysis-relevant datasets directly after HPLC system startup, but instead give the system some equilibration time and precede relevant HPLC runs with at least one blank injection running through a full gradient of the chosen HPLC method.

Importantly, MOCCA requires the user to provide HPLC runs with blank injections for baseline correction. This blank injection run must be recorded in a highly reproducible fashion since it will be subtracted from all subsequent runs. Therefore, it should not be recorded directly after system start-up and should be prceded by at least one more blank injection run. More information on which runs are required for which data analysis features can be found below (section S4).

In summary, the following rules should be followed for successful MOCCA analysis: All HPLC sequences should be preceded by two blank injection runs (or gradient runs without injections). Moreover, pure standard HPLC runs must be performed for all compounds which should be assigned and tracked and for which the peak deconvolution feature should be activated.

Finally, the peak deconvolution feature for overlapping peaks is designed to cover the case when a known analyte is overlapped by an unknown and unexpected impurity. HPLC method development should be performed to a point that all known analytes are baseline-separated.

S4) Technical description of implemented data analysis features

In this section, the data analysis features of MOCCA are described in detail. Corresponding to the order in its processing routine, we will start by detailing MOCCA's data analysis capabilities first on the raw data level, then on the aggregate data level, and finally on the user level, i.e., how a user interacts with the tool using JupyterLab Notebooks to set up the analysis and obtaining stand-alone html reports as output. The data analysis features are summarized in Fig. 2 of the main text, with each of its features assigned to the corresponding level in the data–information–knowledge pyramid.

In the following sections, two types of HPLC runs are distinguished, which are called compound runs and analysis runs. For compound runs, the user exactly knows what is in the sample (e.g., a pure analyte with a given concentration) while samples of unknown composition should be analyzed by MOCCA in analysis runs. A set of HPLC–DAD datasets which are analyzed in context to each other is subsequently called a MOCCA campaign.

Raw data preprocessing

The raw data preprocessing routine is adapted from vendor software which typically provides the two signal settings "bandwidth" and "reference" in the settings panel of diode array detectors (DAD). The bandwidth option is a smoothing filter in the wavelength dimension which averages the signal of the selected wavelength with all signals within the given bandwidth (e.g., for a signal at the wavelength 250 nm and a bandwidth of 20 nm, the signal is recorded as the mean of the raw signals in the wavelength region 240–260 nm).[14] The reference option allows to record – additionally to the given signal wavelength – the signal on a reference wavelength (with a given reference bandwidth), which is subtracted from the respective main signal.[14] This accounts for detector drifts during an HPLC run.

For the implementation in MOCCA, we adapted these settings to the full absorbance data array. The bandwidth configuration is set to a default value of 2 nm and is implemented as a rolling average over the whole wavelength dimension of the

absorbance array (basically generating a bandwidth of 2 nm for each wavelength in the array). The reference option is implemented by averaging the signals of the highest five wavelengths and subtracting the resulting signal from the signal of each wavelength in the array.

Please note that the UV-Vis range of the DAD detector has to be set to an upper limit of greater than 10 nm higher than any absorbance band of any analyte. If this is not given, the reference signal setting can – as it does in vendor software – cause negative signals on certain wavelengths.

Baseline correction

As in other parts of MOCCA's design, the baseline correction feature uses a mixture of required experiments in the lab together with computational data analysis algorithms. The user is required to give a corresponding blank injection run (called gradient run) for each HPLC–DAD dataset in which we expect signals from analytes (called compound runs). The same gradient run can be used for multiple compound runs but should be renewed once the HPLC instrument was shut down or stopped.

We found that the best practice around these blank gradient runs is to record two blank gradient runs before injecting real samples on the HPLC system. After system startup or long down time at low or without flow, the first gradient is often disturbed and does not represent a reproducible dataset which can be used for baseline correction. In contrast, the second blank injection run typically provides a satisfactory gradient which can be subtracted from the following runs. For long HPLC sequences over multiple hours it is recommended to repeat a blank injection run after reasonable amount of sample injections to adapt for slow drifts and changes in the sytems signal output over time.

MOCCA reads and processes gradient runs with the preprocessing routine described above. For each wavelength of the resulting HPLC–DAD data array, a modified baseline correction algorithm based on asymmetric least squares smoothing is applied on the corresponding chromatogram.[15,16] This levels out minor carryover or impurity signals as well as noise and other artifacts like injection peaks. The resulting data array is subtracted from the corresponding compound runs. Fig. S1 illustrates the

summed absorbance of a gradient run before and after the application of the baseline correction algorithm.

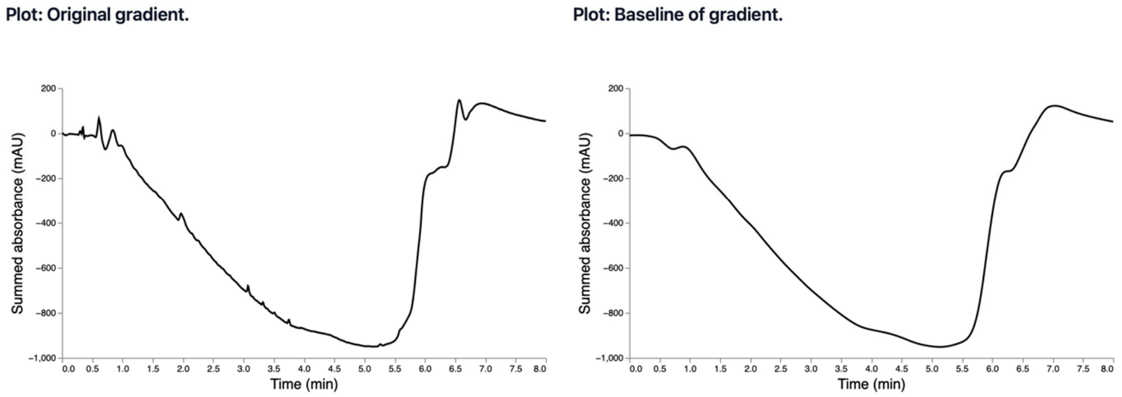

Figure S1. Screenshot out of the gradient report of the MOCCA package. On the left, the summed absorbance chromatogram of the blank gradient run is shown. On the right, the resulting gradient data after baseline correction is shown.

Peak picking

Chromatographically separated signals of different analytes are picked in chromatograms along the retention time dimension. To pick these peaks, MOCCA reduces the dimension of the raw data absorbance array (time–wavelength) by summing up the signals on all wavelengths for each time point. Additionally, the user is given a configuration setting called absorbance threshold, which is a number of summed absorbance. For the remainder of the peak picking routine, all absorbance values lower than the absorbance threshold are set to zero so that a peak maximum has to surpass the absorbance threshold value to be picked as a peak.

This processed summed absorbance chromatogram (time vs. absorbance) is used to pick chromatographic peaks using the Python package SciPy's signal module.[17] This module also provides a function to calculate peak widths, allowing MOCCA to check for peak overlaps. Overlapping peaks are merged to one peak model. It is important to note that with this procedure, overlapping peaks are only merged to one peak if the minimum summed absorbance of the overlap exceeds the absorbance threshold setting.

Finally, the user has control over two additional configuration settings, which are a peaks high pass and a peaks low pass filter. This allows to restrict peak picking in the time dimension of the chromatogram to exclude artifact peaks in the beginning and in the end of the chromatogram from subsequent data analysis (this could be, e.g., the injection peak at the start of the run or signals originating from the re-equilibration of the HPLC column at the end of the run).

Peak expansion

Since all absorbance values below the absorbance threshold were set to zero for peak picking and merging, peak models subsequently have to be expanded to represent the full peaks. For that, a rolling average filter with a window size of 5 is applied to the summed absorbance chromatogram (including the values lower than the absorbance threshold). Left and right boundaries of the peak models are then extended until the smoothed signal falls below 1/20 of the absorbance threshold (considered as baseline) or starts increasing.

Peak purity check

MOCCA checks peak purity via a stepwise testing routine, which is mainly based on the similarity of the UV-Vis spectra across all timepoints between the boundaries of the peak. In theory, a pure peak should have identical UV-Vis spectra across all timepoints, while an impure peak's UV-Vis spectra would generally differ across timepoints. Thus, the correlation coefficients between the UV-Vis spectra at all timepoints to that at the peak apex are computed. The resulting vector of correlation coefficients is then interpreted to predict the peak purity as follows:

1. The first test is based on the unimodality of the correlation coefficient as we range through all timepoints of the peak. We found that pure peaks' correlation coefficients were always unimodal (first increasing, then decreasing) across timepoints, while impure peaks would often not be unimodal. We implemented this test by checking the unimodality of a rolling average (of size 3) of the correlation coefficients, and ignoring any coefficient with value > 0.999 (as there is some small variation in high correlation coefficient regions). If the test fails, then we immediately label the peak as impure. Otherwise, we continue on to the second test.

2. The second test is based on the peak purity checker implemented by Agilent.[18] The correlation coefficient at each timepoint *t* to the apex of the peak is compared with a computed threshold value *thresh*, defined as

$$thresh_{\mathrm{t}} = \left(\max\left(0, 1 - 2.5 \cdot Var_{\mathrm{noise}} \cdot \left(\frac{1}{Var_{\mathrm{t}}} + \frac{1}{Var_{\mathrm{apex}}}\right)\right)\right)^2$$

   where Var_{noise} is the variance of the UV-Vis spectra at a time when no compound is present, Var_{t} is the variance of the UV-Vis spectra at the specific timepoint t, and Var_{apex} is the variance of the UV-Vis spectra at the apex. If 90% or more of the timepoints' correlation coefficients are above their calculated thresholds, the peak is classified as pure. Otherwise, we continue on to the third test.

   This test differs from the original Agilent peak purity checker, in that we use the coefficient 2.5 instead of 0.5, and that the test passes with 90% of good points rather than 100%, making the test more permissive, as the Agilent peak purity checker gave many false negatives in our testing.

3. The third test is based on a Principal Component Analysis (PCA), which attempts to decompose the peak into the sum of components, while maximizing the variance explained by each of the components. If the variance explained by the first component is > 0.995, this means that one component is able to account for much of the elution profile, and so we label the peak as pure. Otherwise, we continue on to the last test.

4. The last test is based on the values of the actual correlation coefficients. If any correlation coefficient is lower than 0.9, then this implies that the peak is most likely impure somewhere, and hence we label it as impure. Otherwise, if the correlation coefficients are all greater than 0.95, or the average coefficient is greater than 0.98, then the correlation throughout the entire spectrum is high and we label the peak as pure.

If none of the above tests returns a conclusion about the peak being pure or impure, the peak is labelled as impure.

Peak integration

One of the advantages of working with the full HPLC–DAD raw data is that UV-Vis active substances cannot "hide" in summed chromatograms – analytes exhibiting significant absorbance at 210 nm can be invisible in a chromatogram recorded at 250 nm. For example, Waters uses this advantage in their Empower software by providing UV-max plots that shows a synthetic chromatogram in which every peak found is drawn at the maximum absorption wavelength.[19]

MOCCA represents this advantage in its integration routine. Integral values are obtained by summation of all absorbance values for each recorded wavelength and for each timepoint in the peak borders. Before doing so, all absorbance values are subtracted by the lowest value in the peak's absorbance array representing a horizontally straight integration baseline in a chromatogram.

Deconvolution of overlapping peaks

Following the textbooks and literature on the deconvolution of overlapping peaks in HPLC–DAD data, an HPLC–DAD absorbance array is bilinear data, and multiple such datasets can be stacked on top of each other to obtain a multiway data tensor which ideally is trilinear.[20] However, the retention time dimension is considered as a trilinearity-breaking mode since elution profiles are not perfectly reproducible over multiple runs (in contrast to the spectral mode where UV-Vis spectra of given analytes will not change between runs). In theory, this disables the use of classical deconvolution algorithms for trilinear data like the PARAllel FACtor analysis (PARAFAC).[21] A schematic principle of the PARAFAC algorithm is shown in Fig. S2.[22] Here, a multiway data tensor is built out of k samples containing an overlapping peak of two components. The PARAFAC algorithm deconvolutes the data tensor providing results for each of the two components in form of an elution profile, a UV-Vis trace as well as the concentration in each sample.

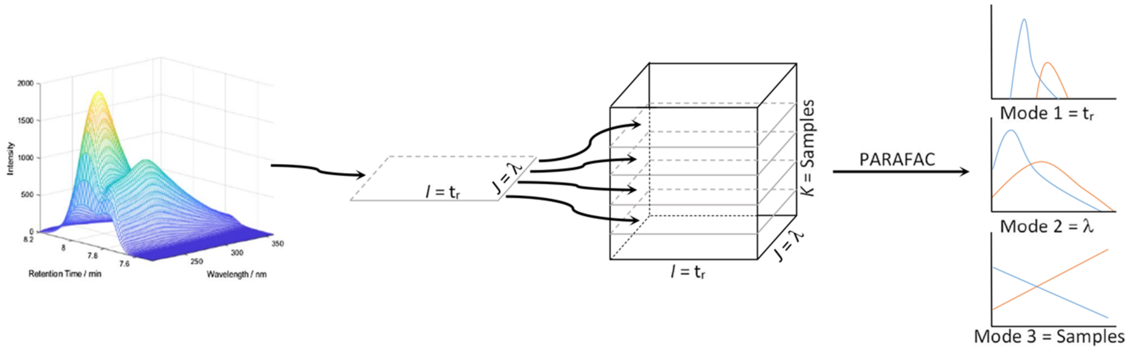

Figure S2. Principle of the PARAFAC algorithm applied to HPLC–DAD data deconvoluting an overlapping peak of two components. Reproduced with permission from O'Hanlon et al.[22]

As discussed above, perfect trilinearity in the data tensor cannot be achieved with real HPLC–DAD data due to the sample-dependent elution profile. In literature, this issue was approached in multiple ways from the algorithmic side, developing more sophisticated algorithms which take the non-trinlinearity into account.[23–25] These algorithms, however, either show generalization issues in real life or are not publicly available for implementation. Considering the use cases for which MOCCA was designed, i.e., the contextual analysis of HPLC–DAD datasets on the same system and column with the same method, a novel and pragmatic approach was developed in this work tackling the problem from the side of how to build the data tensor.

First of all, we decided that not all overlapping peaks in a chromatogram have to be deconvoluted. Typically, we are interested in deconvoluting peaks where an unknown side component unexpectedly overlaps with a known component which was calibrated and which should be quantified. Therefore, MOCCA's deconvolution routine is only triggered for peaks which did not pass the purity check (peak is labled as impure) and which overlap with a known compound model in the library (see below for details in the section compound models/library). For these peaks, data tensors are generated with the following empirically developed routine which allows the usage of the classical PARAFAC algorithm, in spite of the trilinearity-breaking retention time mode.

A data tensor is generated from three slices (absorbance arrays), of which the first two slices contain absorbance arrays of pure compound peaks, while the last contains the absorbance array of the overlapping peak. The two pure compound peaks are aligned to the compound maximum in the compound library (see below in section compound

models/library for details). Moreover, these two peaks are normalized to half the peak maximum height of the impure peak. Deconvolution of the resulting data tensors using PARAFAC requires an additional parameter, which is an estimate of the number of components present in the overlapping peak. To do so, a classical principal component analysis (PCA) is performed. The estimated number of components is the minimum number of principal components needed to surpass a given cumulative explained variance threshold (default value of 0.995).

To address the trilinearity-breaking retention time mode, we made the assumption that the peak shape does not significantly change over the runs and only the peak location varies from slice to slice. An iterative PARAFAC routine was developed, where data tensors are built such that the aligned pure signals remain in place, while the impure signal is shifted time point by time point from the left to the right. PARAFAC deconvolution is performed with the PCA estimated number of components on each of the resulting data tensors. For implementation in Python, the function "non_negative_parafac_hals " from the package TensorLy was selected which has a non-negativity constraint implemented (values in the resulting elution profiles, UV-Vis spectra and concentration must not be below zero).[26] The resulting factors are translated into PARAFAC-modelled peaks, which are summed up to receive a modelled equivalent of the original ovelapping peak. Between this modelled peak and the raw data of the original peak, the (point-by-point) mean squared error (MSE) is calculated for each iteration. The iteration with the lowest MSE describes the impure peak best and the corresponding modelled peaks are fed back to the chromatogram's peak list. With this iterative PARAFAC routine, the peak deconvolution algorithm is able to adapt for offsets in the retention time over runs.

Data visualization (raw data)

Automated visualization of raw data is implemented in the MOCCA package of which examples can be found in the example reports in the HTML SI files. For example, summed absorbance chromatograms are shown in the chromatogram reports with the peaks highlighted in different colors indicating if the peak purity checker returned true (green) or false (red). In the deconvolution reports, details about peak deconvolutions can be found, including the results of the optimization function of the iterative PARAFAC approach.

Compound models/library

Compound models are generated by MOCCA using runs which contain pure compounds (and optionally internal standards), which will be called compound runs in the remainder of this section. Compound runs can be employed by the user to “train” the tool on known compounds, which allows for tracking of this compound as well as deconvolution of impure peaks containing this compound. Together with the HPLC–DAD raw data of the compound run, the user gives information about the sample, i.e., which compound is expected in the data (compound indentifier as a string literal), in which concentration it is present (see below in section calibration models/library) and if internal standards (in a certain concentration) are present. It then assigns the highest non-internal standard peak to the given compound identifier. All other peaks in the chromatogram are labelled as impurities connected to the given compound identifier. This information is stored in the compound models together with the compound’s retention time and UV-Vis spectrum, which will be used to assign peaks in analysis runs.

The main attributes of the compound models are the UV-Vis spectrum and the retention time of the compound. The UV-Vis spectrum of the compound is stored as the average spectrum over all compound runs of the respective compound. Since UV-Vis spectra do not vary over runs, there is no need to “dilute” this information with UV-Vis spectra of later analysis runs which might be affected by minor impurity overlaps. In contrast, the retention time attribute of the compound model is averaged over all occurrences in the campaign. With that, minor systematic retention time drifts over multiple runs can be accounted for. Optionally, filter functions can be implemented, so that, e.g., the retention time attribute of the model is generated only from the last five occurrences of peaks which were assigned to the respective compound.

Compound models are also generated for pure peaks which are not assigned with a compound identifier via compound runs. Unknown peaks in compound runs are labelled as compound impurities. Unknown pure signals in analysis runs are labelled as unknowns with an iterative counter. This allows tracking impurities over all chromatograms of the current campaign although the user did not provide a compound run of the isolated analyte.

All generated compound models are stored in a library and used as the basis for peak assignment in analysis runs (see below in the section peak assignment for details).

Calibration models/library

Calibration models can only be generated when the user adds compound runs with concentration information to the MOCCA campaign. Calibration models for each of the given compounds are built automatically, where the absorption maximum in the UV-Vis spectrum of the corresponding compound model is selected (remember that this signal already has implemented the bandwidth setting, see above in section raw data preprocessing). On this signal wavelength, the absorbance values between the peak borders are summed up to obtain an integral value. All concentration–integral pairs are used to fit a linear model ($y = mx$). The calibration factors m and the coefficients of determination (R^2 values) are stored for each compound. Calibration relative to internal standard signals is also implemented and described in detail below (see section internal standard handling).

The generated calibration models are stored in a library and used to quantify peaks (translate integrals to concentrations) which were assigned with the matching compound ID in analysis runs.

Internal standard handling

Handling internal standard signals in chromatograms is a time-consuming task in manual analysis since it requires extra integration and calculation steps. In MOCCA, internal standard handling is automated in the context of two tasks, i.e., retention time correction and relative quantification.

For the retention time correction task, the internal standard signal is found in the chromatogram before peak assignment and deconvolution of impure peaks, where only pure peaks with matching UV-Vis spectrum and retention time are considered. In the case that there is an impure peak in the internal standard's retention time region, this peak will be deconvoluted using the pure internal standard signal to build the data tensor for the PARAFAC algorithm. If the internal standard peak was found, the maximum of its retention profile is compared to the library entry of the internal standard (see above in the section compound models/library) and the difference is saved as

offset in all peak objects of this chromatogram. For subsequent peak assignment, the corrected retention times are compared against the entries in the compound library.

For the relative quantification task, MOCCA allows for fully automated generation of calibration models with respect to internal standard peaks. If a known internal standard signal is present in datasets of calibration runs, the concentration of the given compound c_A is plotted against the intergral of the signal corresponding to the compound I_A multiplied by the given concentration of the internal standard c_{ISTD} and divided by the integral of the internal standard signal I_{ISTD}. A linear fit ($y = mx$) gives a calibration curve with a slope m.

$$c_{\mathrm{A}} = m\,\frac{I_{\mathrm{A}} c_{\mathrm{ISTD}}}{I_{\mathrm{ISTD}}}$$

If an internal standard is in use, MOCCA always generates both a classical absolute calibration model (integral vs. concentration) as well as the above-described relative model. For the quantification of peaks in analysis runs, the relative quantification is used as default option.

Peak assignment

The peak assignment in MOCCA follows a two-step process. First, each pure peak is assigned to possible matches. Then, peaks are assigned to compound identifiers in the context of all peaks with matches in the chromatogram to avoid duplicate assignments.

Possible matches of a peak are obtained using the compound library (see above in the section compound models/library). Two threshold settings given by the user define if a peak matches with a compound. The first threshold is a relative retention time threshold which describes a value of time points (relative to the total number of time points in the chromatogram) which a peak is allowed to be shifted in order to be considered as a possible library compound. As an example, in an HPLC method with a length of 10 min (600 s) and a given relative retention time threshold of 0.01, a peak with a retention time of 5 min (300 s) requires a library compound with a retention time of 294–306 s to be considered as a possible match, i.e., the retention time of a peak

observed within an analysis run lies in the borders of ±0.01 · 600 s of the library compound's retention time.

The second threshold is a UV-Vis spectrum correlation threshold. Here, only peaks are considered as possible matches to a library compound if the correlation coefficient between the average UV-Vis spectrum over all time points of the peak and the library spectrum exceeds the given threshold. Matches are assigned to the peak only if **both** conditions are satified, i.e., the peak resembles the library compound sufficiently in retention time and UV-Vis spectrum.

In the second step, peaks with possible matches are assigned to compound identifiers. For that, the peak which has the highest UV-Vis correlation coefficient over all peaks and all matches in the chromatogram is assigned to the respective compound identifier. This compound identifier is deleted from all possible matches of the other peaks. This process is repeated until all peaks are assigned or peaks are left with no matches. Pure peaks without matches are labelled as unknowns or impurities and are added to the compound library.

Compound tracking

Since all peaks in all chromatograms of a MOCCA campaign are assigned to library compounds if possible, corresponding data such as integrals and concentration can be easily tracked over runs. Even if a compound was only found by peak deconvolution, the corresponding peak is added to the peak list of a chromatogram and is therefore trackable.

Peak quantification

Once a peak is assigned to a compound identifier, MOCCA checks if this compound identifier is also present in the library of calibration models. If so, the concentration of the compound is quantified using the summed absorbance at the calibration wavelength and the calibration factors of the linear models (for details see section calibration models/library). If internal standard was added, the peak is quantified in relation to the integral of the internal standard signal and the given concentration (for details see section internal standard handling).

Data visualization (aggregate data)

Examples for automated visualization of aggregate data can be found in the example reports in the HTML SI files. For example, integrals and concentrations of each compound can be tracked visually over all chromatograms in the compound tracking report. Calibration curves and UV-Vis spectra of the compounds are automatically plotted in the calibration library and compound library reports, respectively.

User control over data analysis

The user has control over a number of settings to influence the data analysis features of MOCCA. The high pass and low pass filters for retention time as well as the absorbance threshold are discussed in sections peak picking and peak expansion (see above). The relative retention time threshold and spectral correlation coefficient threshold are discussed in the section peak assignment (see above). Additionally, the user can restrict the wavelength dimension which MOCCA employs for data analysis using the wavelength high pass and low pass filter (high pass should be set to >200 nm typically). With the detector limit setting, the user can give an absorbance value which should not be exceeded by the detector at any given time point and wavelength. If this absorbance value is exceeded in a peak, the peak is labelled as saturated and the user should check if it is affected from saturation effects.

As discussed in chapter S2, raw data files are exported in a number of different file formats depending on the instrument's control software or the export framework. For each of the above-described data formats, MOCCA provides a parser, i.e., a routine how to read these data in a Python environment. The user gives MOCCA the information about the file format using the labels 'chemstation' for data retrieved from Agilent's Chemstation with the 3DExport macro, 'labsolutions' for data exported from Shimadzu's Labsolutions software, 'empower' for data from Waters' Empower software and 'allotrope' for adf files following the LC–UV Allotrope Data Model. Finally, the user can add simulated or otherwise retrieved data to MOCCA by using the 'custom' tag. For details, see the documentation of the code.

Adding user input to MOCCA for analysis requires a certain semantic which is described in detail in the code documentation as well as in the accompanying JupyterLab notebooks (for first steps see tutorial.ipynb in the GitHub repository).[27]

Results reporting

MOCCA feeds back automatically generated reports about the data analysis to the user. As an html framework, we employed the Python package Datapane[28] which allows to create interactive reports as stand-alone html files. Examples reports can be found in the HTML SI files or can be generated using the notebooks and example data in the GitHub repository.[27] To avoid file sizes becoming too large and reports becoming unresponsive, we split the reporting about a campaign in logical components:

1. hplc_input.html: All HPLC input as given by the user is listed sorted in the order as MOCCA processes the input.
2. gradient.html: All given gradients are plotted together with the smoothed version which is used for baseline correction.
3. chromatograms.html: All chromatograms are plotted with picked peaks, indicated peak purity and (if triggered) centers of deconvoluted components. Moreover, a peak table is provided including the main data analysis results for this chromatogram.
4. bad_chromatograms.html: All chromatograms where MOCCA could not proceed with data analysis due to inconsistencies between user input and the actual data. For example, if the user provides a compound indicating a compound run and the highest peak in the chromatogram is impure, the chromatogram is put in the bad data container and reported in this report.
5. deconvolution.html: For impure peaks, for which the deconvolution feature was triggered, details about the peak deconvolution process are provided. Most importantly, the modelled retention profiles UV-Vis traces are plotted.
6. peak_library.html: All peaks found in all chromatograms of the campaign are put in one large interactive dataframe.
7.compound_library.html: All compound models, knowns and unknowns, of the campaign are shown (both knowns and unknowns) including their UV-Vis spectra and retention times.
8. calibration_library.html: If calibration runs were provided by the user, this report shows details about the calibration models including a calibration plot with linear fit.
9. compound_tracking.html: For each compound of the compound library, the summed peak absorbance, area percent, and (if applicable) concentration is plotted against the chromatogram number in the campaign. This allows to visually follow the presence of a compound over all given runs.

S5) Validation of quantification feature

The quantification of signals with the MOCCA approach was validated against the typical manual approach following HPLC best practices. For that, the three substrates of the Knoevenagel study (see main text) were calibrated using all five employed HPLC methods with varying gradient lengths and data were analyzed with both approaches. Each calibration procedure was repeated once to increase the statistical power of the analysis by duplicate experiments.

Notably, we faced typical failed integration cases during the manual control of the integration routine. Fig. S3 shows screenshots from the Chemstation reports in which the integration algorithm cannot recognize the difference between the gradient and real analyte signals.

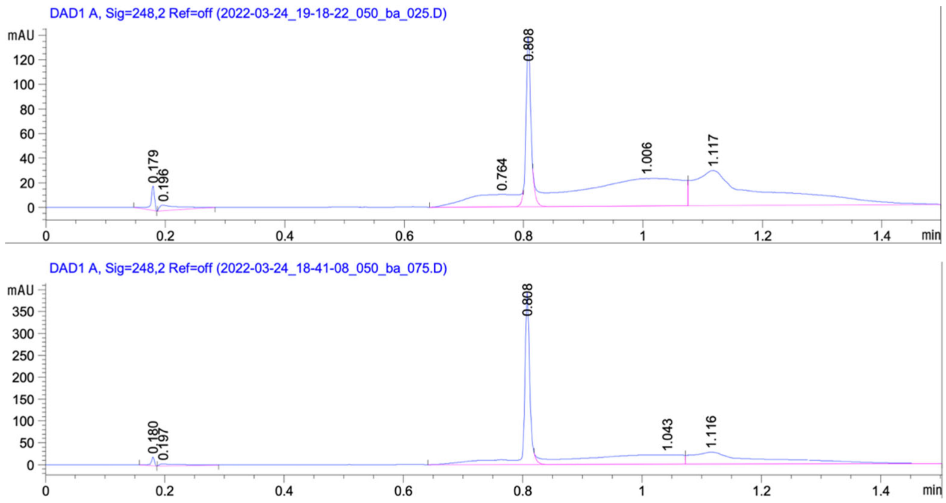

Figure S3. Typical failed integrations by the vendor software's automated integration routine.

Calibration curves derived from MOCCA and manual analysis for all three benzaldehyde analytes and all five HPLC methods with varying gradient lengths (see main text for details) are shown in Fig. S4. Note the difference in the y-axis labels as MOCCA sums up absorbance values while the Chemstation software provides peaks areas as read-out. It can be clearly observed that both quantification methods lead to very comparable results. Not only are the calibration curves in the correct orientation relative to each other, but the single calibration points relative to their calibration curve also show a very similar pattern for both methods.

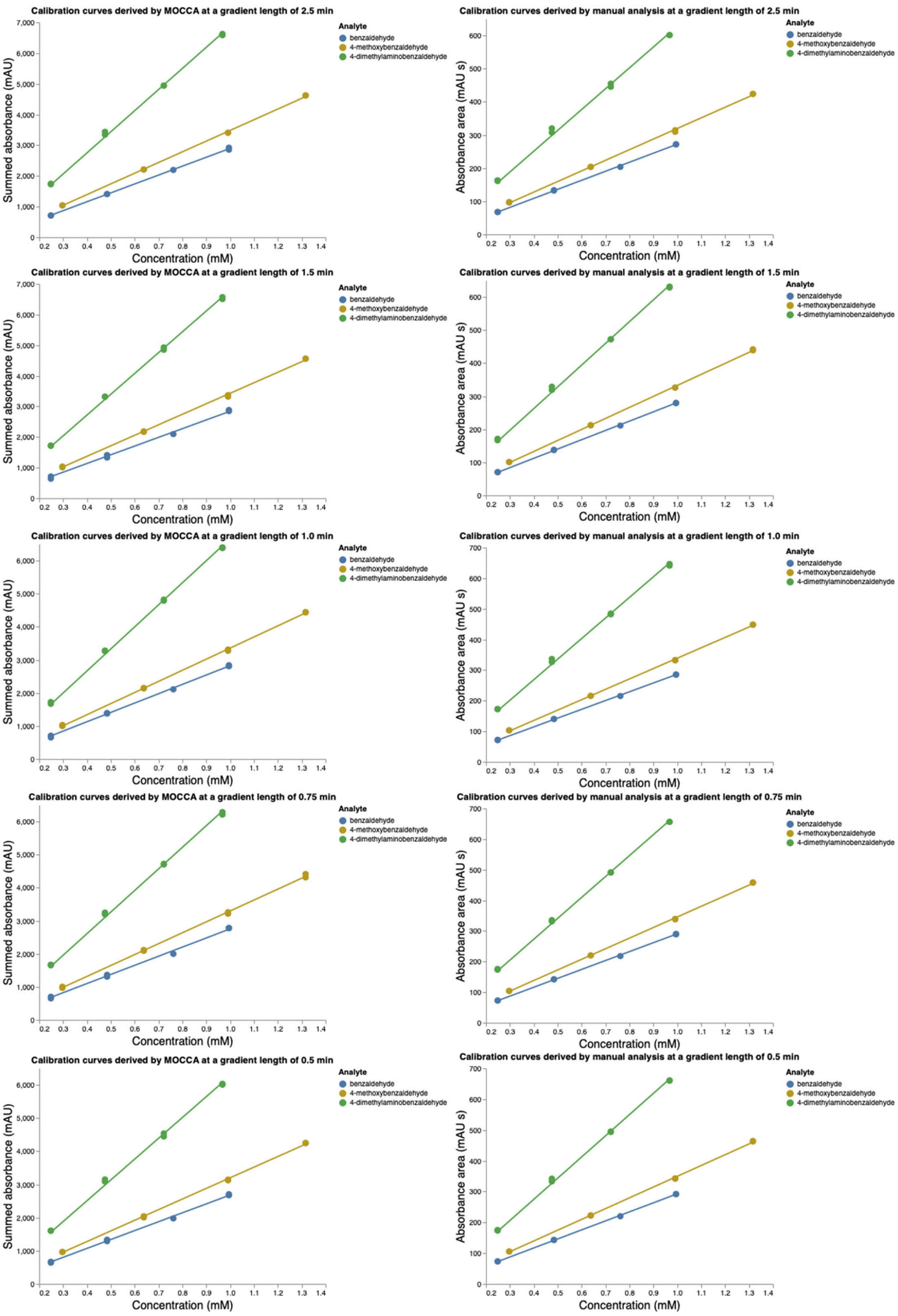

Figure S4. Calibration curves of benzaldehyde, 4-methoxybenzaldehyde, and 4-(dimethylamino)benzaldehyde at different gradient lengths in HPLC methods (see plot labels) derived by MOCCA (left column) and by manual analysis (right column).

To verify MOCCA's quantification accuracy in the case of baseline-separated pure peaks, value-by-value correlations between both analysis methods are plotted in Fig. S5 for each gradient length. The almost perfect correlation between both methods validates that the quantification feature of MOCCA delivers precise quantification results.

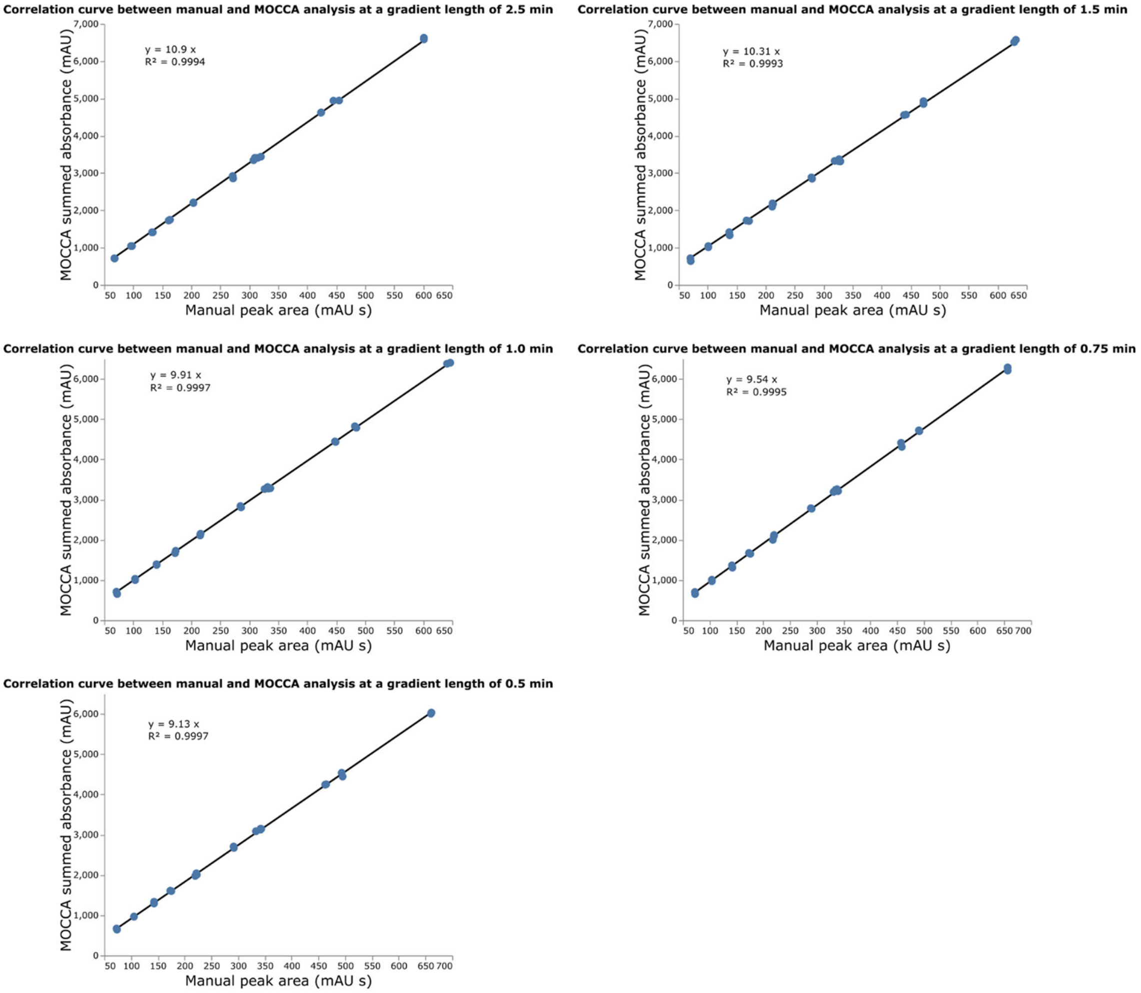

Figure S5. Correlation plots of values derived by manual and by MOCCA analysis for each gradient length.

S6) Kinetics study of Knoevenagel condensation reactions

HPLC instrumentation

For this study, we employed an Agilent LC system including a degasser (G1379B), a binary pump (G1312B), an autosampler (G1329B) with thermostat (G1330B), a thermostatted column compartment (G1316B), and a diode array detector (G1315B). As a reversed-phase separation column, we used a 50 x 3 mm Kinetex UPLC column (00B-4462-Y0) with 2.6 µm core-shell particles (C18 with TMS endcapping). We used water deionized by a Milli-Q gradient system and acetonitrile (HPLC grade) as eluents and added to both 0.05% (v/v) trifluoroacetic acid (HPLC grade).

HPLC methods

We developed five HPLC methods with different gradient lengths (between 2.5 min and 0.5 min) to induce different grades of overlapping signals of the analytes of this study. For all HPLC methods, the autosampler was temperature-controlled at 25 °C and took sample sizes of 1 µL out of the reaction vessels. The column compartment was temperature-controlled at 25 °C. The diode array detector was set up to record a full UV-Vis spectrum at every time point of the chromatogram (200–550 nm, step size of 1 nm). For classical calibration analysis, single-wavelength signals were recorded on the wavelengths of absorbance maxima of the analytes (248 nm for benzaldehyde, 283 nm for 4-methoxybenzaldehyde, and 347 nm for 4-(dimethylamino)benzaldehyde).

The mobile phase flow rate was set to 1.5 mL min^{-1} for all five HPLC methods. Methods started with a hold of 0.1 min on 95:5 water/acetonitrile (v/v). Then, five different linear gradient lengths from 95:5 water/acetonitrile (v/v) to 0:100 water/acetonitrile (v/v) were programmed (0.5 min, 0.75 min, 1.0 min, 1.5 min, and 2.5 min). After the gradient, a hold of 0.1 min on 0:100 water/acetonitrile (v/v) followed before a steep gradient of 0.1 min back to 95:5 water/acetonitrile (v/v) started the re-equilibration process of the column. HPLC–DAD raw data were exported automatically after each HPLC using the Export3D macro or the Allotrope adapter (see section S2).

Kinetics studies

For reaction kinetics studies, reactions were set up in HPLC vials to be run directly in the thermostatted autosampler of the HPLC system. This allowed the following of reaction progress in the same vial with all five employed HPLC methods iteratively. Reactions were set up gravimetrically with a substrate concentration of 1.0 mmol L^{-1} (1 mL volume) using stock solutions of the substrates. Both the two-substrate and the three-substrate reactions were run at concentrations of benzaldehydes and malononitrile of 1.0 mmol L^{-1}. Reactions were started by injecting pure piperidine (0.02 mL, 0.2 mmol) into the solution.

Data analysis

For the data analysis described in the main text, benzaldehyde was added to the MOCCA campaign as main compound while the other two benzaldehydes were treated as “unknown” impurities. The corresponding signals were labelled as designated unknowns by MOCCA and could be tracked over the runs in the MOCCA reports. The deconvolution tool was able to return retention profiles and UV-Vis traces of the impurities which allowed MOCCA to assign the correct unknown label to the modelled peak in all instances (two- and three-substrate cases).

S7) Validation of data analysis features in simulated chromatograms

As described in the main text, we employed the Chromatography Analysis and Design Toolkit (CADET) to simulate a variety of different retention profiles to investigate MOCCA's peak deconvolution feature on a statistically significant scale.[29] To increase the variety of peak shapes, we turned to the simulation of ion-exchange chromatographic separation of two analytes employing a gradient with linearly increasing salt concentration. We configured CADET to solve the transport-dispersive model of liquid column chromatography, in which all mass transfer resistances are lumped into a single film diffusion parameter. The model was completed by the nonlinear steric mass action model, which describes competitive adsorption including steric shielding effects.

For generating test datasets, we randomly sample the isotherm parameters (equilibrium constant, characteristic charge, steric shielding constant) while keeping the operating conditions (e.g., salt profile, loading concentrations, flow rates) fixed. CADET produces a fully resolved chromatogram, i.e., the concentration of each compound at the column outlet is returned at each second.

To investigate MOCCA's peak deconvolution feature, CADET was used to simulate three retention profiles for one MOCCA campaign, i.e, a pure compound run of the main compound (given to MOCCA as a calibration run with a relative concentration of 1), a pure compound run of the impurity (given to MOCCA as an analysis run, so that it is added to the compound library as an unknown) and an analysis run with both compounds present (given to MOCCA as an analysis run). As a result, we expect MOCCA to label the main compound peak as "comp1" (given by the user) and the impurity peak as "unknown_1" (given by MOCCA). In all simulations, both compounds are added with a relative concentration of 1.

In a second step, the obtained retention profiles were augmented with pairs of UV-Vis spectra as shown in Fig. S6. For each CADET-simulated set of three retention profiles (two pure, one overlapping), the retention profiles of the main compound were

augmented with the UV-Vis spectrum of 4-chlorobenzaldehyde (blue spectra in the spectrum panel of Fig. S6) while the retention profiles of the impurities were augmented with the UV-Vis spectra of benzaldehyde (yellow), 4-methoxybenzaldehyde (green) or 4-(dimethylamino)benzaldehyde (red). The resulting HPLC–DAD datasets are packed together to MOCCA campaigns as shown in Fig. S6.

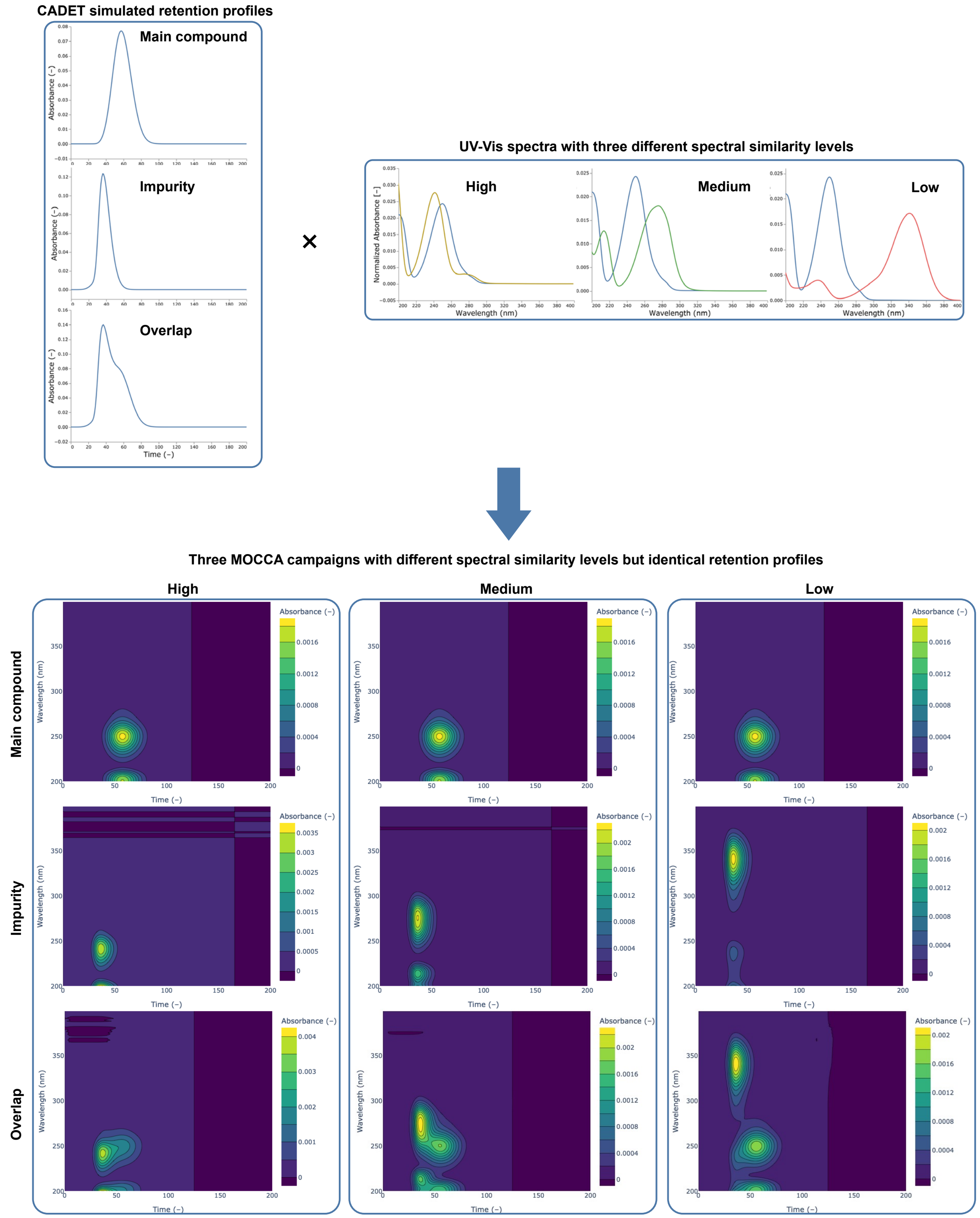

Figure S6. Workflow of in-silico generation of HPLC–DAD datasets used as MOCCA campaigns. First, three chromatograms are simulated using CADET, i.e., first a pure run of the main compound, second a pure run of the impurity and third a chromatogram containing both the main compound and the impurity. The retention profiles are augmented with three pairs of UV-Vis spectra resembling three levels of spectral similarity. The resulting nine HPLC–DAD datasets are packed together to MOCCA campaigns in sets of three and were directly used for data analysis.

These campaigns are subsequently analyzed by MOCCA and the results are fed back to the user by reports. In Fig. S7, typical visualizations of these reports are shown as screenshots (analysis results of the data shwon Fig. S6). Reports are generated automatically by MOCCA upon data analysis. For each spectral similarity level, the overlapping retention profiles are shown in the upper panes together with the resulting peak table. MOCCA finds the presented peak to be impure (indicated by the red-colored area behind the peak), triggers the peak deconvolution feature and finds two components whose retention maxima are indicated by the vertical dashed lines. The peak table correspondingly lists the impure peak (peak_id 1) together with the two modelled component peaks (peak_ids -1 to indicate that their origin is the peak with the peak_id 1). In this case, MOCCA assigns both traces correctly, i.e., the main compound is listed as “comp1” and the impurity is listed as “unknown_1”. Much more information is included in the table (see additional ESI material for example reports) including the assigned compound name, the integral, and the quantified concentration of the main compound.

In the lower panes of Fig. S7 the modelled retention profiles and UV-Vis spectra for the two components are shown as results of the peak deconvolution feature. The modelled retention profiles and UV-Vis traces nicely resemble the given input as shown in Fig. S7.

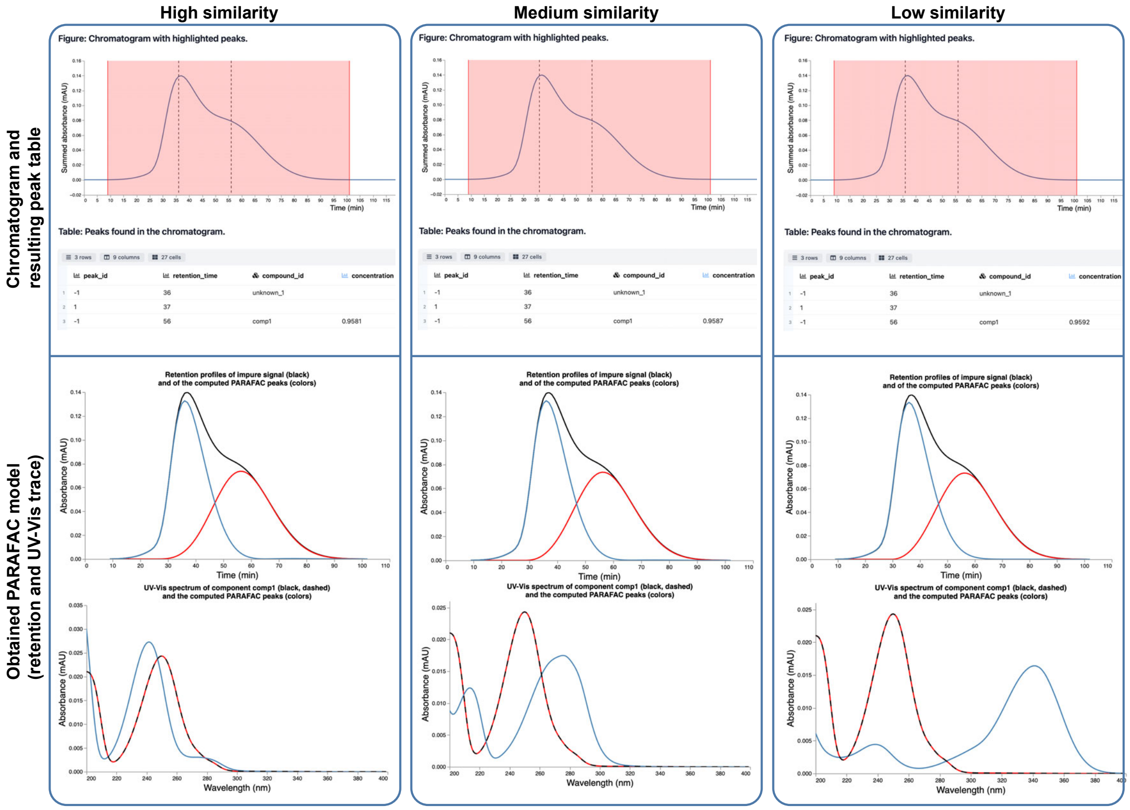

Figure S7 Typical result for the analysis of in-silico generated HPLC–DAD data. For each spectral similarity level, the upper panes show the chromatograms (all identical since the same retention profiles were used) together with the resulting peak table. The peak in the chromatogram is highlighted red to indicate that it is impure and the vertical dashed lines indicate the retention maxima of the two modelled components. The lower panes show the corresponding retention and UV-Vis traces of the two modelled components.

Each result of the 3000 MOCCA campaigns is assigned to one of four results categories (see main text for details) based on the resulting peak table of the two-component chromatogram. For each of the categories, a typical example is shown in Fig. S8. Each example comprises the two simulated retention profiles of the pure main compound and of the pure impurity as well as the two-component chromatogram. Please note that the pure impurity run solely serves to control if MOCCA is able to resolve the UV-Vis trace of the impurity correctly (discussed below in more detail). Results of the category (iii) are very rare and both cases were found to be resulting from highly co-eluting retention profiles of compounds which, on their own, generate bimodal retention profiles in their pure compound runs as seen in Fig. S8c. This nicely highlights that, if the peak deconvolution feature is triggered, the qualitative analysis (finding the correct UV-Vis trace for peak assignment) is highly reliable.

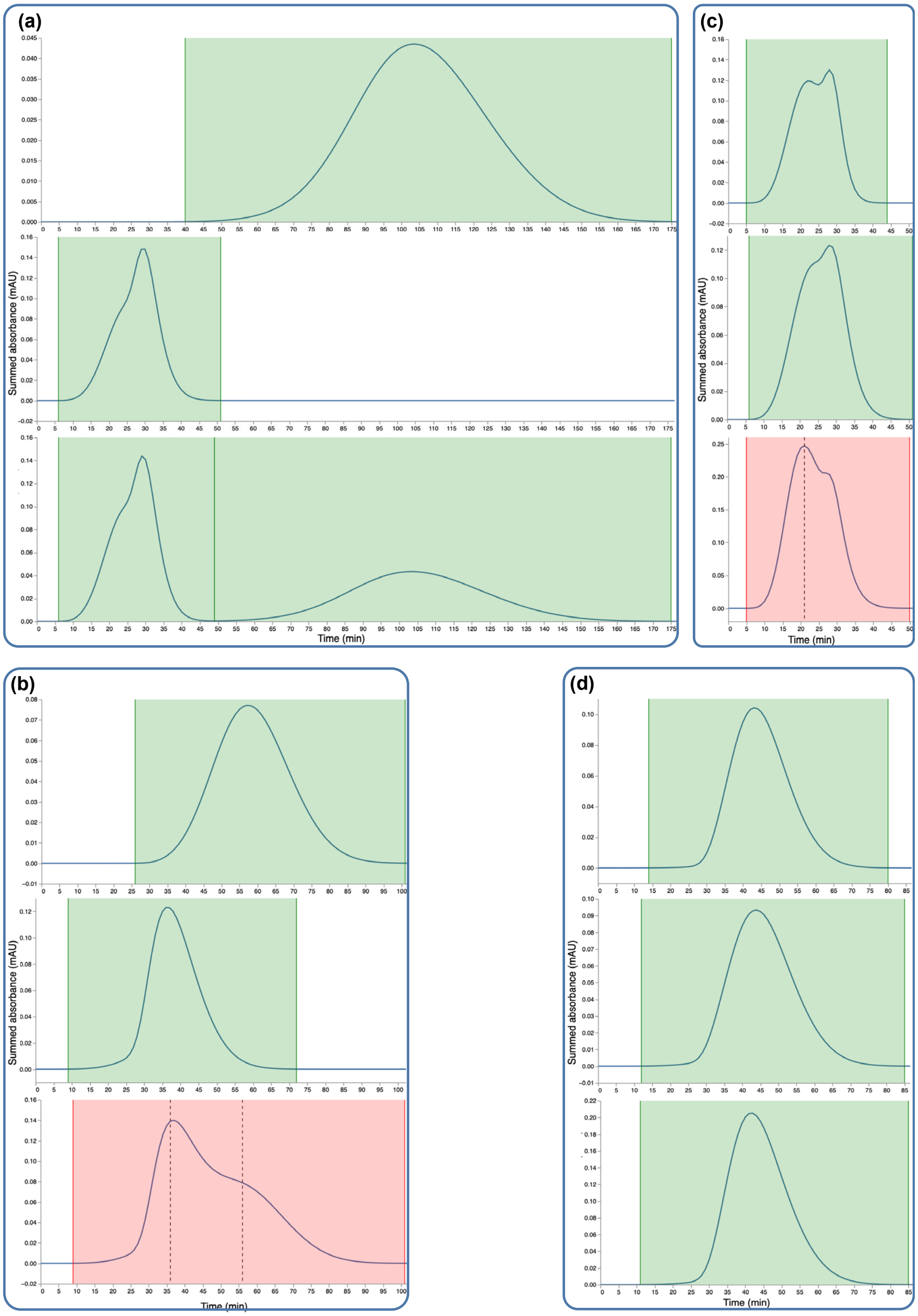

Figure S8. Example MOCCA campaigns (two pure compound runs, one two-compound run) for each of the four results categories **a)** (i): Peaks are baseline-separated, **b)** (ii): signals are overlapping, MOCCA labels the overlapping peak as impure (indicated by the red area), the deconvolution feature is triggered and found two components with the retention maxima indicated by the dashed vertical lines, **c)** (iii): signals are overlapping, MOCCA labels the overlapping peak as impure but is not able to resolve the main compound's identity for the high spectral similarity level, **d)** (iv): Signals are co-eluting almost perfectly and the peak is therefore labelled as pure by the peak purity checking feature.

For results of category (ii), a quantitive investigation of the error range followed. Fig. S9 shows the quantitative error of the category (ii) datasets for each spectral similarity level as boxplots with the datapoints jittered in the background and outliers not removed. Additionally, the jittered points are colored green, when the peak deconvolution tool not only found the correct UV-Vis trace of the main compound but also of the impurity while they are labelled red when only the UV-Vis trace of the main compound could be extracted correctly. This nicely highlights that a bad quantification result often comes hand in hand with the tool not finding the correct UV-Vis trace of the impurity. These cases appear more often in the datasets of the medium and high spectral similarity levels since they originate from very complex deconvolution problems, i.e., highly co-eluting retention profiles of the two compounds. In the case of high spectral similarity, these strongly co-eluting signals are often not even detected as impure by the peak purity checker and are therefore assigned as category (iv) result (see Table 1 in the main text for more details).

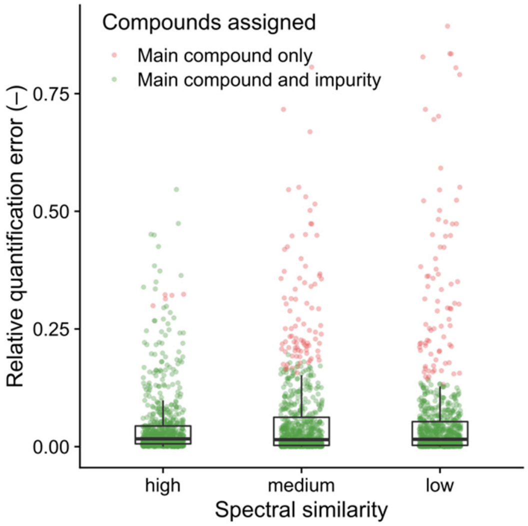

Figure S9. Boxplots and jittered data points of the mean squared error (MSE) between the quantification value obtained by deconvolution and the ground truth for results assigned to category (ii). The color of the jittered points indicates if MOCCA's peak deconvolution feature was only able to obtain the main compound's UV-Vis trace (red) or it could also assign the impurity correctly (green). For the high spectral similarity level (dataset size of 794 points), the median MSE is 1.6% and the third quartile MSE is 4.4%. For the medium spectral similarity level (dataset size of 868 points), the median MSE is 1.5% and the third quartile MSE is 6.2%. For the low spectral similarity level (dataset size of 890 points), the median MSE is 1.6% and the third quartile MSE is 5.3%.

In the following, we present in Fig. S10–S12 for each similarity level an example which is in the range of the third quartile of quantification error. This should give the reader a feeling on how complex the deconvolution problems already are in these regions. As shown in the figures, peaks with a quantification error close to the third quartile are already heavily overlapping and would probably be considered only as one peak by a human eye (see Fig. S12). For those strong co-eluting cases, we consider quantification errors in the range of 5% as completely acceptable.

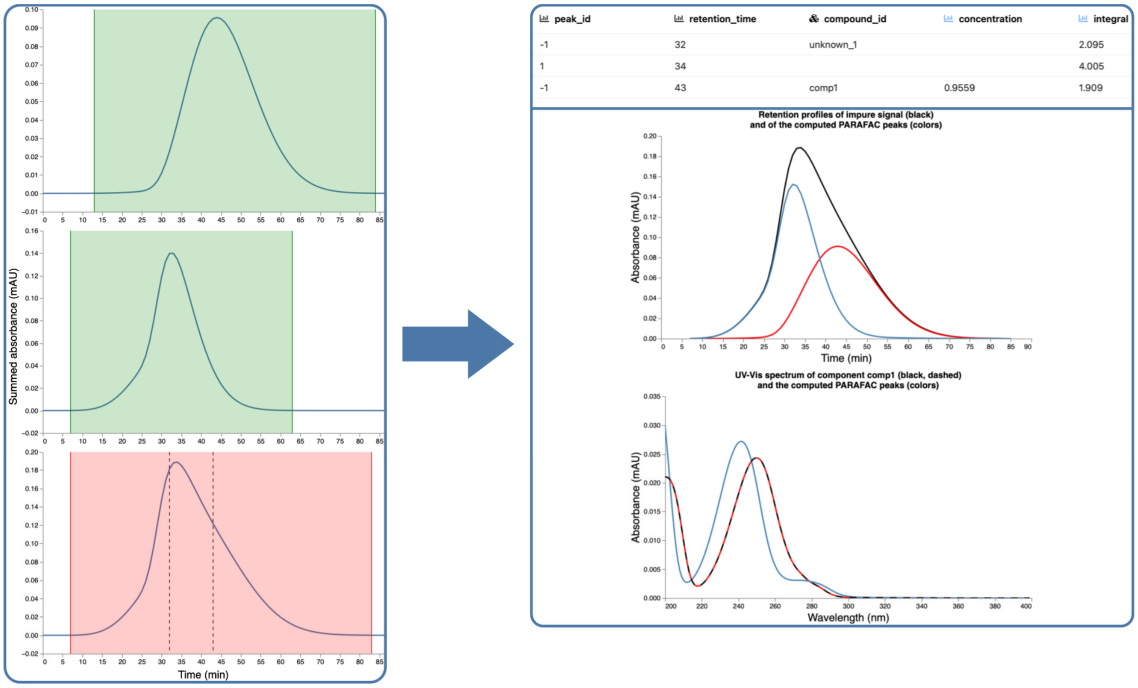

Figure S10. Example of a deconvolution problem near the third quartile quantification error (4.4%) for the high spectral similarity level.

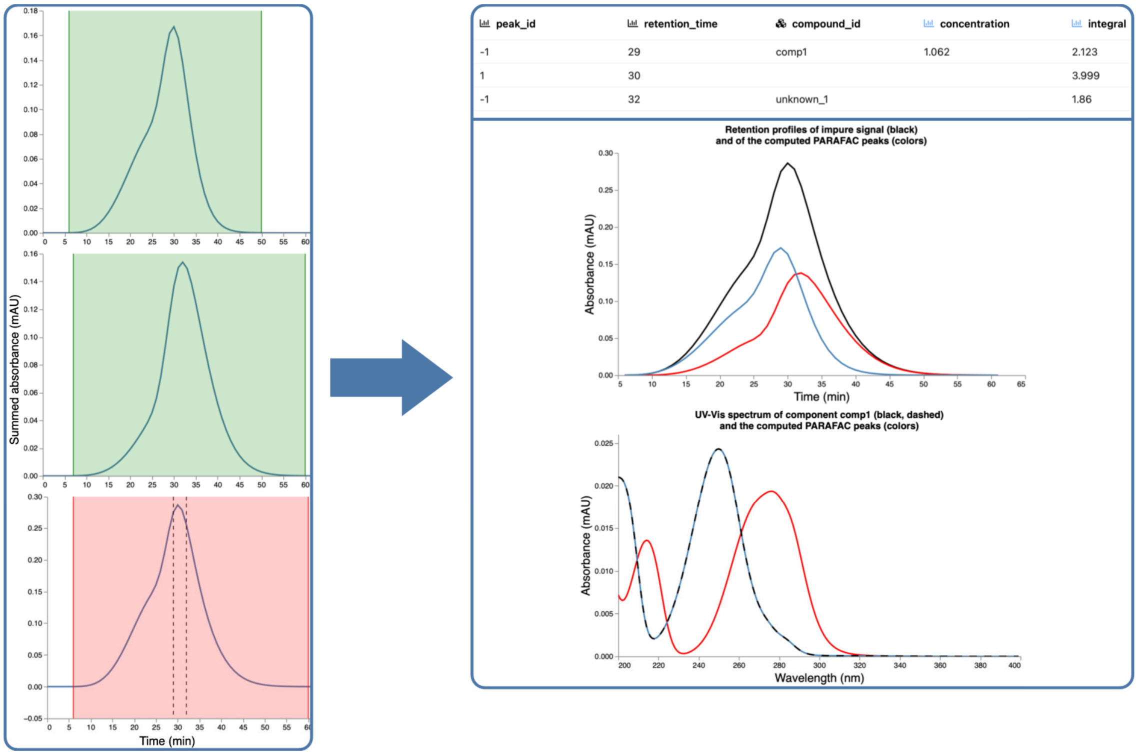

Figure S11. Example of a deconvolution problem near the third quartile quantification error (6.2%) for the medium spectral similarity level.

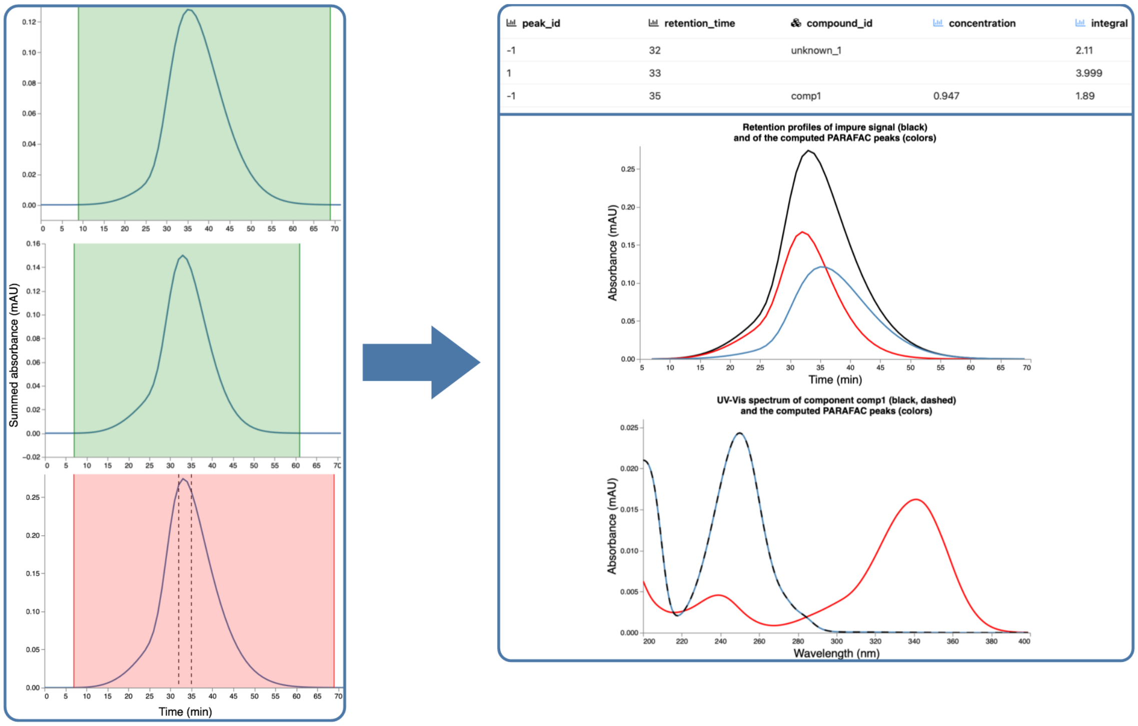

Figure S12. Example of a deconvolution problem near the third quartile quantification error (5.3%) for the low spectral similarity level.

S8) Closed-loop optimization of the alkylation of 2-pyridone

General workflow

We built the closed-loop optimization platform on the microfluidic droplet platform as previously reported by our group elsewhere.[30] Here, we discuss only the changes made on the reported platform which were required for this study.

Firstly, we exchanged the optimizer from the MATLAB based mixed-integer nonlinear program (MINLP) to the Python-based EDBO[31] and also moved the analytical data processing from MATLAB (automatic read-out of vendor software reports) to Python. We established communication protocols with a MATLAB object called “slug” as the central control instance of the optimization platform. Fig. S13 outlines the data workflow of the platform. The slug object communicated with EDBO via csv files (which EDBO supports as an export module). Communication with the microfluidic droplet platform took place via MATLAB script nodes in the accompanying LabVIEW program. Since direct communication with the HPLC control software (OpenLab ChemStation Edition) was challenging, we opted to send electric signals to the remote port of the HPLC instrument to trigger the start of a pre-loaded HPLC method. After the HPLC run, HPLC–DAD data were exported automatically and the path to the corresponding raw data file was written in the slug object. Communication with MOCCA took place via json strings and analytical results were also written in the slug object.

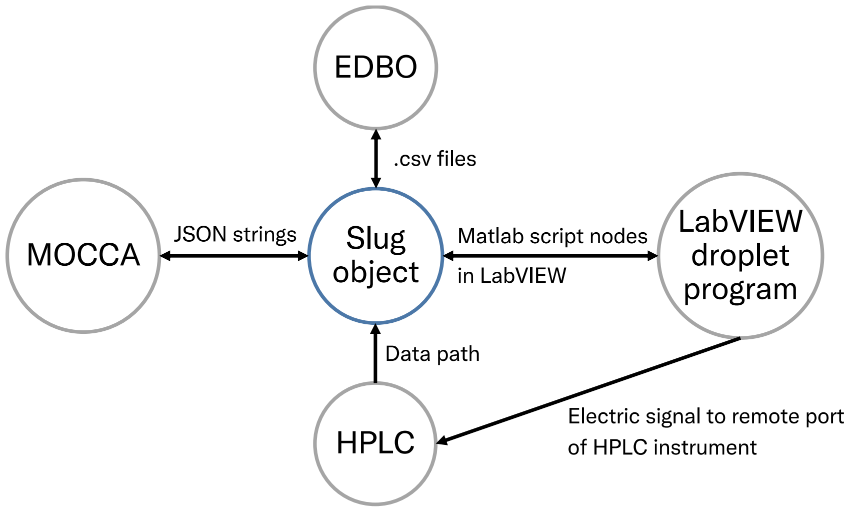

Figure S13. Data workflow of the close-loop optimization platform with the MATLAB slug object as the central process control.

Experimental design via Bayesian optimization

We replaced the previously implemented MINLP optimizer with a Bayesian optimizer developed by the Doyle group, named EDBO.[31] The categorical variables, i.e., the base and solvent, were represented by one-hot encoding. Within EDBO, continuous variables are typically entered in a discretized form. We, thus, linearly mapped the inverse of the temperature ($\frac{1}{T+273.15K}$) and the logarithm of the reaction time in ten equidistant steps within the given domain spaces. The obtained values were transferred back into the respective temperature and time values to be used as input for the optimizer. For initialization, a random experiment was chosen out of all possible variable combinations. The batch size was set to one, meaning that after every single experiment the optimizer was rerun suggesting one new experiment that was then performed. As an acquisition function, we used the default expected improvement setting. The campaign's objective was to maximize the yield of *N*-butyl-2-pyridone (**6**).

Microfluidic droplet platform

The hardware of the microfluidic droplet platform remained mainly untouched compared to the state we reported on previously.[30] A minor change was made in the oscillatory reactor design by moving inlet and outlet of the horseshoe-shaped reactor closer to each other. This improved the robustness of the droplet control on the reactor, i.e., droplets of different organic solvents could be robustly detected on the inlet and outlet of the reactor with the narrower light path. Fig. S14 shows a photo of the new reactor design.

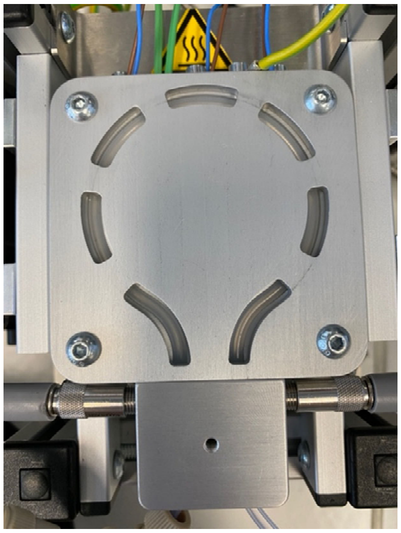

Figure S14. Photograph of the optimized reactor design. The horseshoe-shaped reactor was closed a bit more to move reactor inlet and outlet closer together. This allowed for more light of the photodetector (optic fiber cables attached to the reactor perpendicular to the reactor tubing) passing through the reactor inlet and outlet.

Another significant change was made to the HPLC injection valve. Here we opted for an internal injection valve with exchangeable rotors for sub-microliter injection volumes of 20 nL, 50 nL, or 100 nL (Vici, C84H-1574-.02EUHF). In the presented study, we chose the rotor with 20 nL injection volume.

HPLC analysis

The same HPLC system was used as in the Knoevenagel study (details in section S6). We changed the flow path of the HPLC system so that the autosampler was bypassed and the internal injection valve was instead implemented as the injection source.

HPLC self-calibration of the platform

We developed a method for the platform to calibrate the analytical signals in the HPLC itself. We put stock solutions of the internal standard tetralin and the product *N*-butyl-2-pyridone in the liquid handler of the microfluidic droplet platform and replaced the EDBO optimizer by a hard-coded calibration procedure. The system prepared calibration droplets and sent them through the reactor. After the reactor, they were diluted with acetonitrile exactly like the subsequent reaction droplets would be. Injections took place using the 20 nL internal injection valve. All concentration information was automatically passed to MOCCA using the MATLAB slug object

(Fig. S13). The resulting calibration curves for the product are shown in Fig. S15 as screenshots from the corresponding MOCCA reports. MOCCA also creates calibration models for the absolute signal area, but uses the calibration model relative to the internal standard for subsequent product quantification.

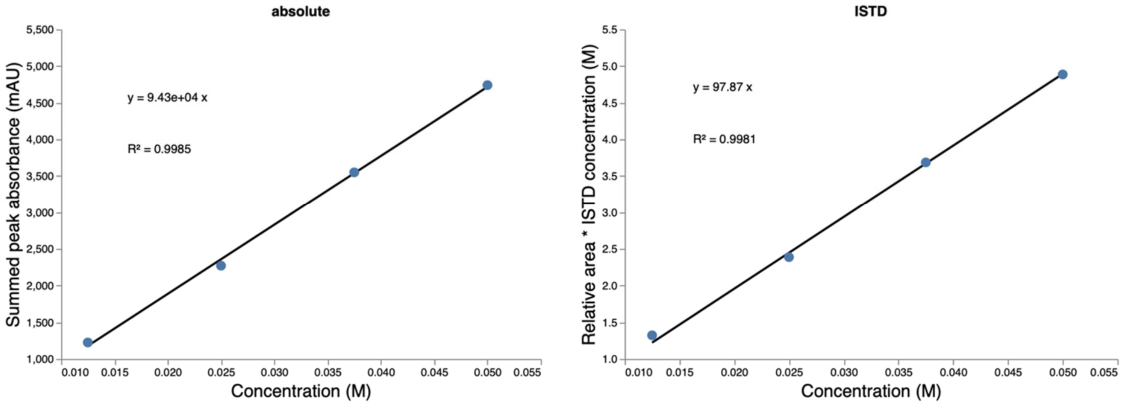

Figure S15. Calibration curves of *N*-butyl-2-pyridone (1-butylpyridone in the main text) derived by self-calibration. *Left*: calibration of absolut signal areas; *right*: calibration of signal areas relative to signal areas of internal standard.

Synthesis of *N*-butyl-2-pyridone

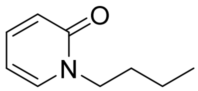

A slurry of 2-hydroxypyridine (0.95 g, 10 mmol, 1.0 equiv.), 1-bromobutane (2.0 mL, 19 mmol, 1.9 equiv.), potassium carbonate (1.38 g, 10.0 mmol, 1.0 equiv.), and potassium iodide (approx. 1 mg) in methanol (10 mL) was heated to reflux for 18 h. After cooling to room temperature and filtration, the solution was diluted with water and dichloromethane. The phases were separated and the aqueous phase was extracted with additional dichloromethane twice. The organic phases were dried over sodium sulfate and concentrated under reduced pressure. *N*-butyl-2-pyridone (1.1 g, 7.3 mmol, 73%) was obtained as a colorless liquid after flash column chromatography (ethyl acetate/ petrol ether; 3:2). Rf in ethyl acetate/ petrol ether (3:2): 0.29.

^{1}H NMR (400 MHz, $CDCl_3$): δ 7.31 – 7.19 (m, 2H), 6.54 – 6.50 (m, 1H), 6.15 – 6.08 (m, 1H), 3.93 – 3.85 (m, 2H), 1.77 – 1.63 (m, 2H), 1.42 – 1.27 (m, 2H), 0.92 (t, *J* = 7.3 Hz, 3H). ^{13}C NMR (101 MHz, $CDCl_3$): δ 162.7, 139.3, 137.6, 121.2, 105.9, 49.7, 31.41, 19.9, 13.8.

The procedure was based on a literature report.[32] The ^{1}H NMR and ^{13}C NMR spectra are in accordance with the data reported therein.

Synthesis of 1-Butyl-2,3,4,6,7,8,9,10-octahydropyrimido[1,2-*a*]azepin-1-ium iodide/triflate

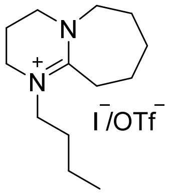

1,8-Diazabicyclo[5.4.0]undec-7-ene (DBU, 0.05 g, 0.3 mmol, 1 equiv.) and 1-iodobutane (0.17 g, 0.90 mmol, 3 equiv.) were dissolved in butanol (3 mL). The reaction mixture was stirred at 100 °C for 12 hours. A noticeable colour change from clear to pale yellow occured. After removal of the solvent under reduced pressure, the crude product was purified by preparative HPLC (Gilson System 2, YMC-Trait Prep C18-S, 12nm S = 5 or 10 µm). 1-Butyl-2,3,4,6,7,8,9,10-octahydropyrimido[1,2-*a*]azepin-1-ium iodide/triflate was obtained as a yellow oil. The trifluoroacetic acid added to the HPLC solvent (partially) replaced the iodide anion.

^{1}H NMR (600 MHz, DMSO-d$_6$) δ 3.61 (m, 2H), 3.45 (m, 6H), 2.84 (m, 2H), 1.96 (m, 2H), 1.68 (m, 2H), 1.62 (m, 4H), 1.51 (m, 2H), 1.29 (m, 2H), 0.90 (t, J = 7.4 Hz, 3H). ^{13}C NMR (151 MHz, DMSO-d$_6$) δ 166.0, 158.4 (q, J = 35 Hz,), 116.1 (q, J = 293 Hz), 54.0, 52.9, 48.5, 46.6, 30.3, 27.8, 27.1, 25.5, 22.8, 19.6, 19.1, 13.8. The spectra were recorded on a Bruker Avance Neo spectrometer operating at 600.34 MHz.

The ^{1}H and 13C NMR spectra are in accordance with literature data for the bromide salt.[33]

Identification of side product

Use of DBU as the base lead to the formation of a side product that was overlapping with the product peak in the HPLC chromatogram. We hypothesized that DBU might react with the butyl iodide, instead of the 2-pyridone substrate. Indeed, the retention time and UV-Vis spectrum of butylated DBU match the characteristics of the observed side product.

Verification of optimization results

We verified the results of the closed-loop optimization campaign by running reactions with every possible combination of discrete variables at 35 °C as well as 100 °C. For

that, a stock solution (1.0 mL) of 2-pyridone (0.05 M), butyl iodide (0.1 M), and 4,4′-di-tert-butyl-biphenyl (0.01 M) in the respective solvent (butanol, toluene, or DMF) was added to a vial filled with the respective base (0.125 M; DBU, TMG, or DIPEA). The solutions were stirred at 35 °C or 100 °C for 60 min. Aliquots of the crude reaction mixture were diluted with acetonitrile and then analyzed by HPLC. The obtained HPLC raw data were exported as .csv files using a post processing plugin tool developed by Angi GmbH (developed for Agilent OpenLab CDS Data Analysis; Version 2.5) and analyzed using MOCCA. The results are shown in Fig. S16 and nicely validate the results obtained during the closed-loop optimization campaign (see Fig. 6a in the main text).

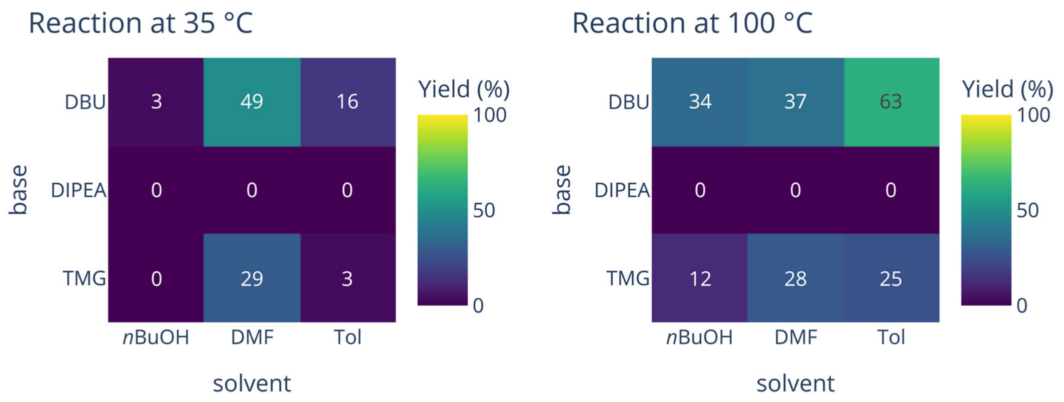

Figure S16. Yields of 1-butylpyridone obtained for the butylation of 2-pyridone with different bases in different solvents at 35 °C and 100 °C after 60 min.

S9) Palladium-catalyzed cyanation of aryl halides

Safety

Cyanohydrins, as well as protected cyanohydrins, are highly toxic and must be handled with great care. The release of HCN must be avoided, e.g., by keeping a basic pH in reaction, work-up, and waste solutions. There may be local regulations that need to be followed. All experiments should be performed in a well-ventilated fume hood and in closed systems, e.g., pressure vessels or septum capped glassware that may be connected to a Schlenk line for pressure compensation.

Chemicals

[(Cinnamyl)PdCl]$_2$ (CAS: 12131-44-1), XPhos (CAS: 564483-18-7), *t*BuXPhos (CAS: 564483-19-8), and CM-Phos (CAS: 1067883-58-2) were purchased from Strem Chemicals. DBU, DMAP, and DIPEA were purchased from Sigma-Aldrich. Dry *n*-butanol (AcroSeal), dry CPME (AcroSeal) and TMG were purchased from Thermo Scientific Chemicals. 2-Chlorotoluene was purchased from TCI America.

HPLC analysis

HPLC analysis was performed on a Nexera series Shimadzu UHPLC with an SPD-40M UV-Vis detector. DAD data was collected from 190 nm to 400 nm with a resolution of 1.3 nm in a temperature-controlled flow cell at 40 °C. A Waters Acquity 2.1 x 50 mm UPLC column was used for reversed-phase separation with C18 functionalized 1.7 µm BEH particles.

GC analysis

Gas chromatographic analyses were perfomed on an Agilent Technologies 7890B GC system that was connected to an FID and an Agilent Technologies 5977B MSD.

Mass Spectrometry

High-resolution ESI-MS spectra were recorded on an Agilent 6545 mass spectrometer coupled to an Agilent Infinity 1260 LC system. High resolution DART-MS spectra were recorded on an EOL AccuTOF 4G LC-plus equipped with an ionSense DART (Direct Analysis in Real Time) source.

Chromatography

Thin-layer chromatograms were obtained with glass-backed silica gel plates (60G, F_{254}, Merck) and visualized by either UV fluorescence (254 nm) or by standard $KMnO_4$ stain followed by heating. For flash chromatography, Silicycle SiliaFlash P60 silica was used.

Synthesis of *O*-protected cyanohydrins

All reactions were performed in dried glassware with anhydrous solvents under nitrogen atmosphere.

2-Cyanopropan-2-yl benzoate (**10a**)

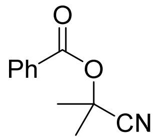

4-Dimethylaminopyridine hydrochloride (79 mg, 0.50 mmol, 5.0 mol%) was added to a mixture of acetone cyanohydrin (0.91 mL, 10 mmol, 1.0 equiv.) and benzoyl chloride (1.3 mL, 11 mmol, 1.1 equiv.). The mixture was stirred at 110 °C for 4 h. After cooling down to room temperature and addition of petrol ether the resulting suspension was filtered. The resulting solution was filtered a second time through a silica plug and eluted with a mixture of petrol ether and diethyl ether (5:1; +0.3 vol% NEt_3). The solvent was removed under reduced pressure and 2-cyanopropan-2-yl benzoate (0.71 g, 3.8 mmol, 38%) was subsequently obtained after flash column chromatography (petrol ether/ diethyl ether; 50:1 to 10:1) as a colorless liquid. Rf in petrol ether/ diethyl ether (3:1): 0.38.

^{1}H NMR (400 MHz, $CDCl_3$): δ 8.04 – 7.99 (m, 2H), 7.63 – 7.56 (m, 1H), 7.49 – 7.42 (m, 2H), 1.90 (s, 6H). ^{13}C NMR (101 MHz, $CDCl_3$): δ 164.6, 133.8, 129.9, 129.2, 128.7, 119.5, 68.9, 27.1.

The ^{1}H NMR and ^{13}C NMR spectra are in accordance with literature data.[34]

2-Methyl-2-((trimethylsilyl)oxy)propanenitrile (**10b**)

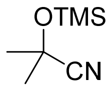

A solution of trimethylsilyl chloride (3.1 mL, 24 mmol, 1.2 equiv.) in tetrahydrofuran (5.0 mL) was added dropwise to a stirred solution of acetone cyanohydrine (1.8 mL, 20 mmol, 1.0 equiv.), and triethyl amine (3.4 mL, 24 mmol, 1.2 equiv.) in tetrahydrofuran (15 mL) at 0 °C. After stirring at room temperature overnight, the solution was poured into diethyl ether (80 mL) and washed with water

(3 × 5 mL), aqueous $NaHCO_3$ (saturated), and brine (5 mL). The solution was then dried over magnesium sulfate and concentrated under reduced pressure (200 mbar, water bath temperature: 40 °C). 2-Methyl-2-((trimethylsilyl)oxy)propanenitrile (0.81 g, 5.1 mmol, 26%) was obtained as a pale brown liquid after distillation at reduced pressure (100 mbar).

^{1}H NMR (400 MHz, $CDCl_3$): δ 1.60 (s, 6H), 0.24 (s, 9H). ^{13}C NMR (101 MHz, $CDCl_3$): δ 122.9, 66.3, 31.0, 1.4.

The procedure was based on a literature report.[35] The ^{1}H NMR and ^{13}C NMR spectra are in accordance with literature data.[36]

2-Cyanopropan-2-yl acetate (**10c**)

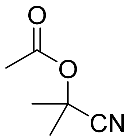

Acetic anhydride (3.5 mL, 37 mmol, 1.5 equiv.) was added dropwise to a solution of acetone cyanohydrin (2.3 mL, 25 mmol, 1.0 equiv.), pyridine (3.0 mL, 37 mmol, 1.5 equiv.), and 4-dimethylaminopyridine (0.30 g, 2.5 mmol, 0.10 equiv.) in dichloromethane (120 mL) at 0 °C. After stirring at room temperature overnight, the reaction was quenched by addition of aqueous Na_2CO_3 (saturated). The phases were separated and the organic phase was then washed with brine twice and concentrated under reduced pressure. 2-Cyanopropan-2-yl acetate (2.1 g, 17 mmol, 66%) was obtained as a colorless liquid after distillation under reduced pressure.

^{1}H NMR (500 MHz, $CDCl_3$): δ 2.08 (s, 3H), 1.74 (s, 6H). ^{13}C NMR (126 MHz, $CDCl_3$): δ 169.0, 119.4, 68.3, 27.0, 21.2. HRMS (ESI): calculated for ([C6H9NO2]+H)$^+$: 128.0706; found: 128.0712.

The ^{1}H NMR spectrum is in accordance with literature data.[37]

1-Cyanoethyl benzoate (**10d**)

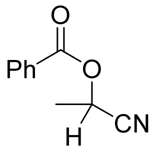

Benzoyl chloride (2.3 mL, 20 mmol, 1.0 equiv.) was added dropwise to a solution of acetone cyanohydrin (1.4 mL, 20 mmol, 1.0 equiv.) in pyridine (7.2 mL) at 0 °C. After stirring for 2 h, brine and diethyl ether were added. The phases were separated and the organic phase was washed with brine twice. The

solution was then dried over magnesium sulfate and the solvent was removed under reduced pressure. 1-Cyanoethyl benzoate (3.2 g, 18 mmol, 91%) was obtained after flash column chromatography (petrol ether/ ethyl acetate; 10:1) as a colorless liquid. Rf in petrol ether/ ethyl acetate (5:1): 0.59.

^{1}H NMR (400 MHz, $CDCl_3$): δ 8.10 – 8.01 (m, 2H), 7.65 – 7.59 (m, 1H), 7.50 – 7.45 (m, 2H), 5.65 (q, *J* = 6.9 Hz, 1H), 1.78 (d, *J* = 6.9 Hz, 3H). ^{13}C NMR (101 MHz, $CDCl_3$): δ 164.8, 134.1, 130.1, 128.7, 128.4, 117.7, 57.9, 19.1.

The ^{1}H NMR and ^{13}C NMR spectra are in accordance with literature data.[38]

1-Cyanoethyl acetate (**10e**)

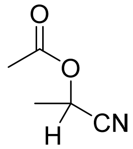

Acetic anhydride (3.1 mL, 33 mmol, 1.1 equiv.) was added dropwise to a mixture of lactonitrile (2.2 mL, 31 mmol, 1.0 equiv.) and 4-dimethyl-aminopyridine hydrochloride (0.24 g, 1.5 mmol, 5.0 mol%) at 0 °C. After stirring overnight, the mixture was diluted with diethyl ether and filtered through a silica plug. The crude mixture was distilled at reduced pressure (150 mbar). The obtained colorless liquid was not sufficiently pure and, therefore, diluted with diethyl ether, washed with aqueous Na_2CO_3 (saturated), and filtered through a silica plug. 1-Cyanoethyl acetate (2.4 g, 21 mmol, 71%) was obtained as a colorless liquid.

^{1}H NMR (400 MHz, $CDCl_3$): δ 5.37 (q, *J* = 7.0 Hz, 1H), 2.12 (s, 3H), 1.62 (d, *J* = 7.0 Hz, 3H). ^{13}C NMR (101 MHz, $CDCl_3$): δ 169.2, 117.6, 57.3, 20.5, 18.9. HRMS (DART): calculated for ([C5H7NO2]+H)+: 114.0550; found: 114.0552.

The ^{1}H NMR spectrum is in accordance with literature data.[37]

1-Cyanocyclohexyl methyl carbonate (**10f**)

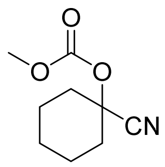

A solution of cyclohexanone (3.1 mL, 30 mmol, 2.0 equiv.), methyl cyanoformate (1.2 mL, 15 mmol, 1.0 equiv.), and 1,8-diazabicyclo[5.4.0]undec-7-ene (DBU, 0.67 mL, 4.5 mmol, 0.30 equiv.) in toluene (15 mL) was stirred at room temperature for 2 h and then filtered through a silica plug. The solvent and residual cyclohexanone was removed *in*

vacuo. 1-Cyanocyclohexyl methyl carbonate (2.4 g, 13 mmol, 87%) was obtained as a pale-yellow liquid.

^{1}H NMR (400 MHz, $CDCl_3$): δ 3.83 (s, 5H), 2.37 – 2.26 (m, 2H), 1.93 – 1.52 (m, 7H), 1.42 – 1.26 (m, 1H). ^{13}C NMR (101 MHz, $CDCl_3$) δ 153.2, 118.3, 75.2, 55.3, 35.1, 24.5, 22.2.

The procedure was based on a literature report.[39] The ^{1}H NMR and ^{13}C NMR spectra are in accordance with the data reported therein.

2-Cyanopropan-2-yl 2,2,2-trifluoroacetate (**10g**)

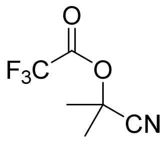

Trifluoroacetic anhydride (1.5 mL, 11 mmol, 1.1 equiv.) was carefully added to a mixture of acetone cyanohydrin (0.91 mL, 10 mmol, 1.0 equiv.) and 4-dimethylaminopyridine hydrochloride (79 mg, 0.50 mmol, 5.0 mol%). After stirring at room temperature overnight, the crude mixture was filtered though a silica plug and eluted with a mixture of pentane and diethyl ether (3:1). The obtained solution was washed with aqueous $NaHCO_3$ (saturated) and brine and then dried over magnesium sulfate. The solvent was removed under reduced pressure and 2-cyanopropan-2-yl 2,2,2-trifluoroacetate (0.99 g, 5.5 mmol, 55%) was obtained as a colorless liquid.

^{1}H NMR (400 MHz, $CDCl_3$): δ 1.88. ^{19}F NMR (376 MHz, $CDCl_3$): δ –75.06. ^{13}C NMR (101 MHz, $CDCl_3$): δ 155.4 (q, J = 43.9 Hz), 117.3, 114.1 (q, J = 286.0 Hz), 72.9, 26.5. HRMS (DART): calculated for ([C6H6F3NO2]+H)$^+$: 182.0423; found: 182.0416.

Well plate screening

Scheme S1 summarizes the conditions for the palladium-catalyzed cyanation of aryl chlorides using protected cyanohydrins.

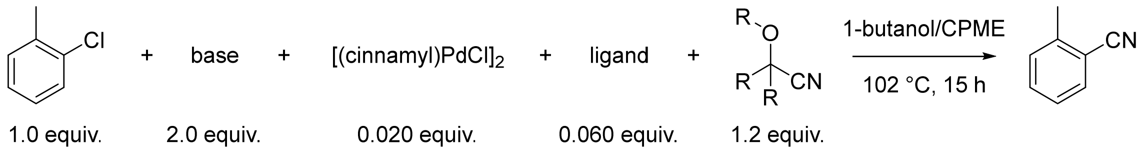

Scheme S1. Reaction conditions for the cyanation of aryl chlorides using [(cinnamyl)PdCl]$_2$ as a catalyst precursor. Bases, ligands, and protected cyanohydrins were screened on the well plate.

For screening different bases and ligands, a 96-well plate was equipped with glass vials and transferred into a glovebox. The vials in rows 1–7 were filled with 0.24 mL of a stock solution containing 2-chlorotoluene (0.25 M) and the respective base (0.50 M) in 1-butanol. Then, 0.060 mL of a stock solution containing [(cinnamyl)PdCl]$_2$ (20 mM) and the respective ligand (60 mM) in cyclopentyl methyl ether were added to these vials. After addition of the *O*-protected cyanohydrin (the addition of the different reagents occurred successively, starting with reagent **10a**, followed by reagents **10b**, **10c**, **10d**, **10e**, **10f**, and lastly **10g**.), the vials were sealed with FEP foil and a layer of rubber septums that were held tight by a metal cover screwed onto the well plate. One of the empty vials in row 8 was filled with DMSO and equipped with a thermocouple. The well plate was placed on a heater shaker unit which was set to a temperature that resulted in an effective temperature of 101–102 °C within the vials. After 15 h, the heating was turned off and the well plate was cooled down to room temperature. Next, the cover was unscrewed in a fume hood and the empty glass vials in row 8 were filled with substrate, product, and blank solvent solutions that were used for calibration. Using a liquid handler, 200 µL of an internal standard stock solution (tetralin, 0.30 M) in acetonitrile were added to each vial. 5 µL of the homogenized samples were placed onto a filter and eluted into a new well plate using acetonitrile. This well plate was placed into the HPLC autosampler of the HPLC system.

Combinations of the following bases, ligands, and cyanide sources were screened.

Bases: 1,8-Diazabicyclo[5.4.0]undec-7-ene (DBU), 1,1,3,3-tetramethylguanidine (TMG), 4-dimethylaminopyridine (DMAP), *N*,*N*-diisopropylethylamine (DIPEA).

Ligands: 2-Dicyclohexylphosphino-2′,4′,6′-triisopropylbiphenyl (XPhos, CAS: 564483-18-7), 2-di-tert-butylphosphino-2′,4′,6′-triisopropylbiphenyl (*t*BuXPhos, CAS: 564483-

19-8), 2-[2-(dicyclohexylphosphino)phenyl]-N-methylindole (CM-Phos, CAS: 1067883-58-2).

O-protected cyanohydrins: 2-Cyanopropan-2-yl benzoate (**10a**), 2-methyl-2-((trimethylsilyl)oxy)propanenitrile (**10b**), 2-cyanopropan-2-yl acetate (**10c**), 1-cyanoethyl benzoate (**10d**), 1-cyanoethyl acetate (**10e**), 1-cyanocyclohexyl methyl carbonate (**10f**), 2-cyanopropan-2-yl 2,2,2-trifluoroacetate (**10g**).

Data analysis

The HPLC–DAD raw data were exported as .txt files and then analyzed using MOCCA. The analysis took 7:50 min on a laptop with Windows operating system using an Intel Xeon W-11955M CPU @2.60 GHz with 64 GB RAM or 10:57 min on a MacBook Pro using a 2,3 GHz 8-Core Intel Core i9 with 16 GB 2400 MHz DDR4. The package was not optimized for performance yet, especially, the iterative PARAFAC algorithm requires significant computation time. Run time of data analysis is therefore strongly dependent on the number of impure peaks which trigger the deconvolution routine (42 in this dataset). The corresponding Jupyter Notebook and .txt files are provided as supplementary data to this manuscript and can be found in the notebooks folder of the GitHub repository.[27] MOCCA allows the extraction of the analysis results in Python and thus enables the automatic generation of customized plots. As an example, a plot of the yield of the reaction in dependence of the location on the well plate is presented in Fig. 7 of the main text. Since MOCCA also tracks the occurrence of unknown compound peaks over several runs, similar visualizations can be generated for side products. In this example, MOCCA identified an unknown side product peak that, based on similarity in retention time and UV-Vis-spectrum, appeared in several chromatograms and was consistently labelled as ‘unknown_3’. Through visualization of its relative peak intensity (Fig. S17), the user can quickly see that this compound only appears in reaction runs in which benzoyl protected cyanohydrins were used. Thus, we hypothesized that the peak can be ascribed to butyl benzoate, the bypyroduct of the transesterification reaction, and confirmed this on basis of the compound’s UV-Vis spectrum.

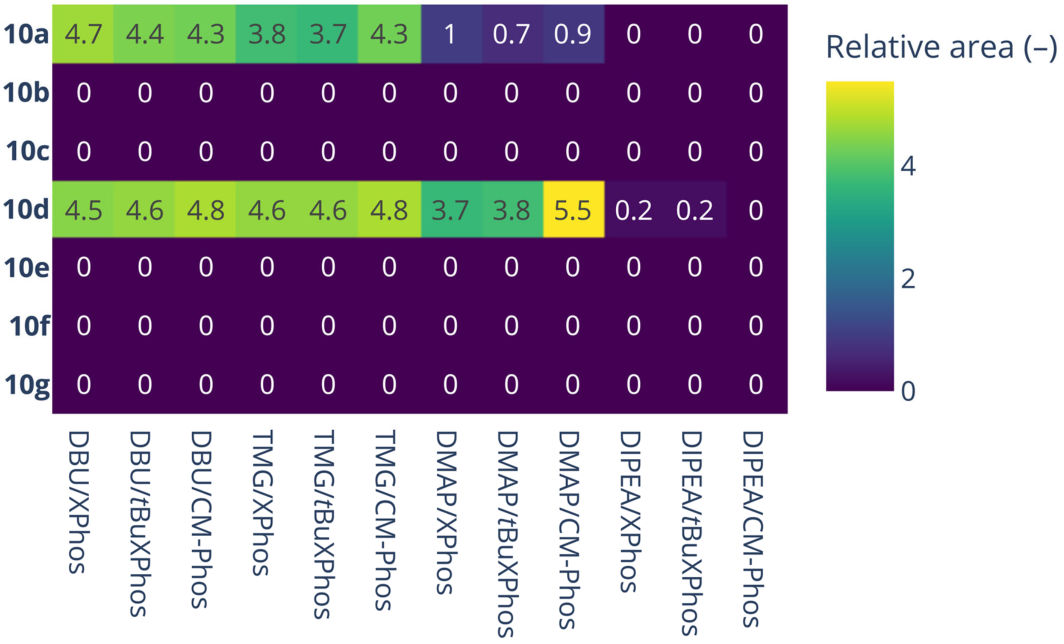

Figure S17. Relative integral plot of an unknown compound ("unknown_3") that MOCCA identified in several reaction runs.

Control experiments

To confirm the results reported in the main text, we repeated some of the well plate experiments according to the conditions shown in Scheme S2.

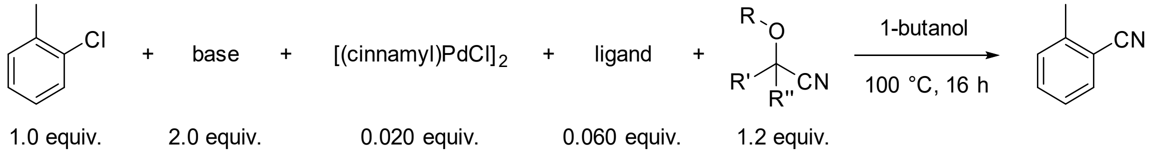

Scheme S2. Reaction conditions for the control experiments of the palladium-catalyzed cyanation of aryl chlorides.

[(Cinnamyl)PdCl]$_2$ (2.1 mg, 4.0 μmol, 0.020 equiv.), the respective ligand (12 μmol, 0.060 equiv.), and the base (if solid, 0.24 mmol, 2.0 equiv.) were weighed into a glass vial and transferred into a glove box. After addition of *n*-butanol (1.0 mL), 2-chlorotoluene (23.4 μL, 0.200 mmol, 1.0 equiv.), base (if liquid, 0.24 mmol, 2.0 equiv.), and the *O*-protected cyanohydrin (0.24 mmol, 1.2 equiv.), the vials were sealed with a septum cap and heated to 100 °C for 16 h outside of the glove box in a metal block. The results are summarized in Table S1.

Table S1. Selected control experiments and corresponding results, which confirm the results from the well plate screening

cyanide source	base	ligand	yield[1]	yield[2]	yield[3]
**10a**	TMG	CM-Phos	69	75	83
**10c**	TMG	XPhos	82	82	93
**10c**	TMG	*t*BuXPhos	64	64	78
**10f**	DMAP	XPhos	99	98	91
**10e**	DMAP	XPhos	98	96	94
**10c**	TMG	CM-Phos	0	0	0

[1]calculated using MOCCA; [2]calculated through manual analysis of calibration and reaction HPLC runs; [3]for comparison: yield of the respective well plate experiment calculated using MOCCA.

Transesterification of *O*-protected cyanohydrins

To examine the rate of deprotection of the differently protected cyanohydrins, we dissolved them in a solution of base and biphenyl (as internal standard) in *n*-butanol and tracked the amount of remaining substrate via GC-FID or HPLC-DAD over time.

In a first study, DMAP was used as a base under the conditions shown in Scheme S3. The protected cyanohydrin (1.0 equiv.) was dissolved in a solution of biphenyl (approx. 0.5 equiv.) and 4-dimethylaminopyridine (2.0 equiv.). After taking a reference sample, the solution was stirred at 100 °C in an oil bath and samples were regularly drawn in order to examine the reaction progress. The samples were worked up by microscale filtration through a pipette filled with little amount of silica.

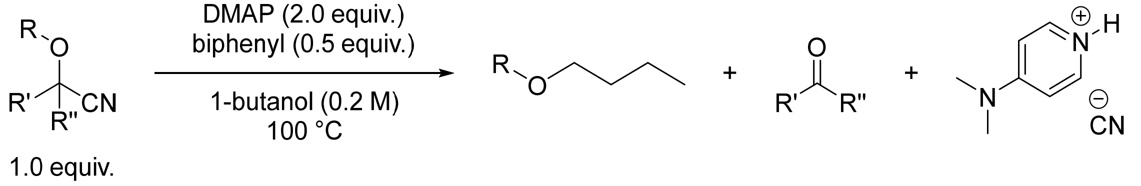

Scheme S3. Transesterification of protected cyanohydrins using DMAP as a base.

The results are plotted in Fig. S18. No signals could be observed for precursor **G**. Considering further observations when performing the reactions, we assume the transesterification to occur rapidly here (note that the results not necessarily indicate an instantaneous decomposition under the described condition as this reagent might

also decompose during workup when exposed to silica). The transesterification for the other substrates occurs over the course of a few hours to several days in the following order: **10f**>**10e**>**10d**>**10c**>**10b**>**10a**.

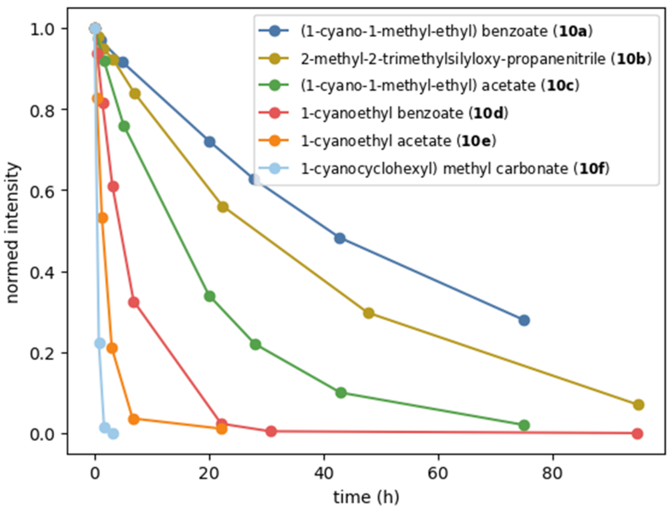

Figure S18. Temporal progression of signal intensities of the protected cyanohydrins when exposed to DMAP in *n*-butanol at 100 °C relative to an internal standard in GC–FID or HPLC–DAD measurements.

In a second study, TMG was used as a base under the conditions shown in Scheme S4. The protected cyanohydrin (1.0 equiv.) was dissolved in a solution of biphenyl (approx. 0.2 equiv.) and 1,1,3,3-tetramethylguanidine (1.67 equiv.). After taking a reference sample, the solution was stirred at 20 °C and samples were regularly drawn to examine the reaction progress. The samples were worked up by dilution with MeCN (for HPLC) or dilution with DCM (for GC).

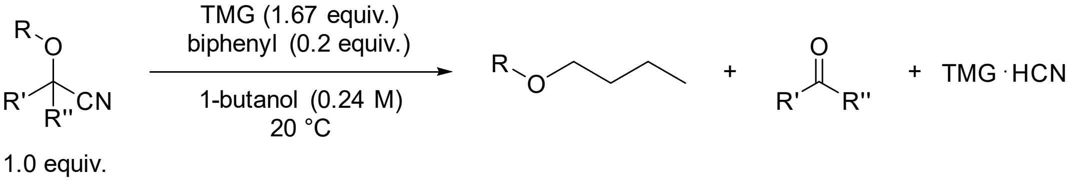

Scheme S4. Transesterification of protected cyanohydrins using TMG as a base.

The results are plotted in Fig. S19. Note that the results shown have been obtained at 20 °C instead of 100 °C. Complete transformation of the proctected cyanohydrins **10e** and **10f** occurs in less than 20 min at 100 °C with TMG as base. Note that the order of reaction rates is different compared to the experiments performed with DMAP:

10e>**10d**>**10f**≈**10c**>**10a**. A possible explanation is a change in reaction mechanisms. While the reaction likely occurs by deprotonation of the *n*-butanol and nucleophilic attack of the *n*-butoxide to the protected cyanohydrin with bases like TMG or DBU, DMAP itself may act as a nucleophile catalyzing the transesterification reaction.

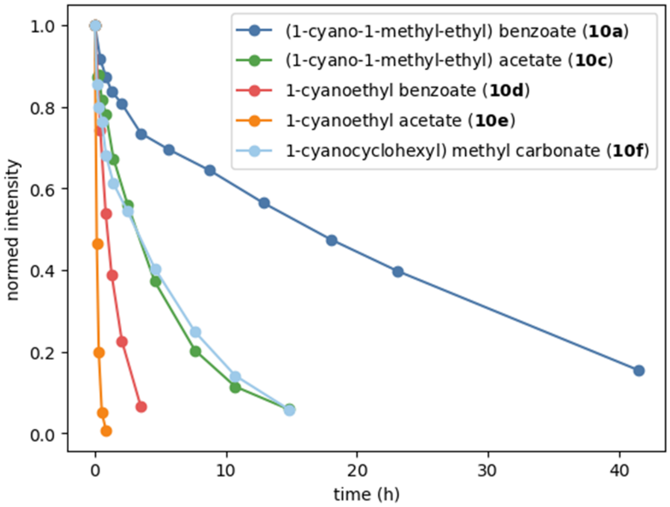

Figure S19. Temporal progression of signal intensities of the protected cyanohydrins when exposed to TMG in *n*-butanol at 20 °C relative to an internal standard in GC–FID or HPLC–DAD measurements.

Detailed interpretation of the well-plate results

As described in the main text, a challenge in palladium-catalyzed cyanation reactions of aryl halides resides in the need for a permanently low concentration of cyanide anions in solution. The rate of cyanide release needs to be adjusted accordingly so that, on the one hand, it is outpaced by the elementary steps of the catalytic cycle, and, on the other hand, it still allows for reasonable productivity. In the following, we discuss the results of the screening shown in Fig. 7e of the main text together with the recorded deprotection rate plots in Figs. S18 and S19.

For trifluoroacetate-protected precursor **10g**, we assume a rapid transesterification to occur under the reaction conditions at high temperatures. Presumably, the release of cyanide is too fast in these cases, leading to a rapid deactivation of the palladium catalyst and, thus, no product formation. It is excluded from further discussions below.

For the different reagents in combination with DMAP, deprotection rates increase from **10a** to **10f**, a trend that is clearly reflected in the reaction outcome of the respective

reactions. The use of DBU as the strongest base tested leads to a rapid cyanide release and, thus, catalyst deactivation resulting in poor reaction outcomes. In contrast, a slow release of cyanide is observed with the use of DIPEA, a weaker base, leading to low productivities. While promising results were observed for *t*BuXPhos in combination with TMG and DMAP, as well as CM-Phos in combination with TMG, the best results under the applied conditions were obtained with the XPhos ligand.

The deprotection of reagents **10d**, **10e**, and **10f** in combination with DMAP occurs at suitable rates under the tested conditions yielding up to 94% of *o*-tolunitrile (**11**). In the presence of TMG, the order of cyanide release rates is different compared to that of DMAP. Specifically, the transesterifications of **10d** and **10e** are faster than that of **10f**. This likely leads to a quick deactivation of the catalyst in these cases while suitable conversion rates for precursors **10a**, **10c**, and **10f** are observed.

When using DBU as a relatively strong base, the transesterification also occurs relatively fast. In further experiments, we could show that the reagents **10e**, **10d**, and **10f** are fully converted within less than 10 min when exposed to DBU in *n*-butanol at 100 °C. The release of cyanide, therefore, likely outpaces the catalyst turnover leading to an inhibition of the desired reaction. Only with the benzoylated acetone cyanohydrin **10a**, which is expected to undergo the transesterification reaction comparably slowly, significant amounts of product were formed. Note that the product formation with reagent **10f** may have occurred for two reasons: Firstly, the rate of transesterification is expected to be lower compared to reagents **10d** and **10e** (see results for rates of transesterification above). Secondly, deactivation of the catalyst may occur at room temperature already and reagent **10f** was the second last to be added to the well plate, meaning that a larger amount of active catalytic palladium species might have been available in this case.

When using TMG, a slightly weaker base compared to DBU, yields of up to 93% are observed with the *O*-protected cyanohydrins **10a**, **10c**, and **10f**. Again, the release of cyanide likely is too fast with reagents **10d** and **10e**.

With DMAP, the reaction yields are in good accordance with the rates of transesterification of the *O*-protected cyanohydrins. Here, faster transesterification

rates lead to a more efficient reaction since they are not outpacing the desired palladium catalyzed reaction steps. The lower yields for reagents **10a**, **10b**, **10c**, and **10d** can be ascribed to an incomplete conversion of the reagent.

The transesterification is too slow if DIPEA is used as a base. In further experiments we could show that hardly any conversion occurs when reagents **10a**, **10b**, **10c**, and **10d** are exposed to DIPEA in *n*-butanol at 100 °C while for reagents **10e** and **10f** only a slow conversion is observed.

As to the ligands, using CM-Phos leads to significant product yields in only a few cases when combined with TMG as a base. Also, the use of *t*BuXPhos did not lead to satisfactory yields. Most promosing results were obtained with XPhos leading to high reaction yields in combination with TMG and DMAP.

Since using TMG already shows significant deprotection rates at room temperature, we expect the DMAP conditions to be more robust experimentally. With TMG, the catalyst may be potentially deactivated in course of the reaction setup.

Peak deconvolution examples

Here, we show two examples of peak deconvolution, which enabled HPLC–DAD data analysis of the well plate screening without the need for the development of an optimized HPLC method. Fig. S20 shows an example where an unknown impurity overlaps with the product peak. MOCCA modelled retention profiles and UV-Vis traces of two compounds, of which one is assigned to the product compound identifier. After peak assignment, MOCCA is able to quantify the modelled peak using the calibration library.

Fig. S21 shows another example where an unknown impurity overlaps with the internal standard peak. MOCCA is able to find the internal standard signal in the overlapping peak and uses the modelled internal standard peak for retention time correction and relative quantification.

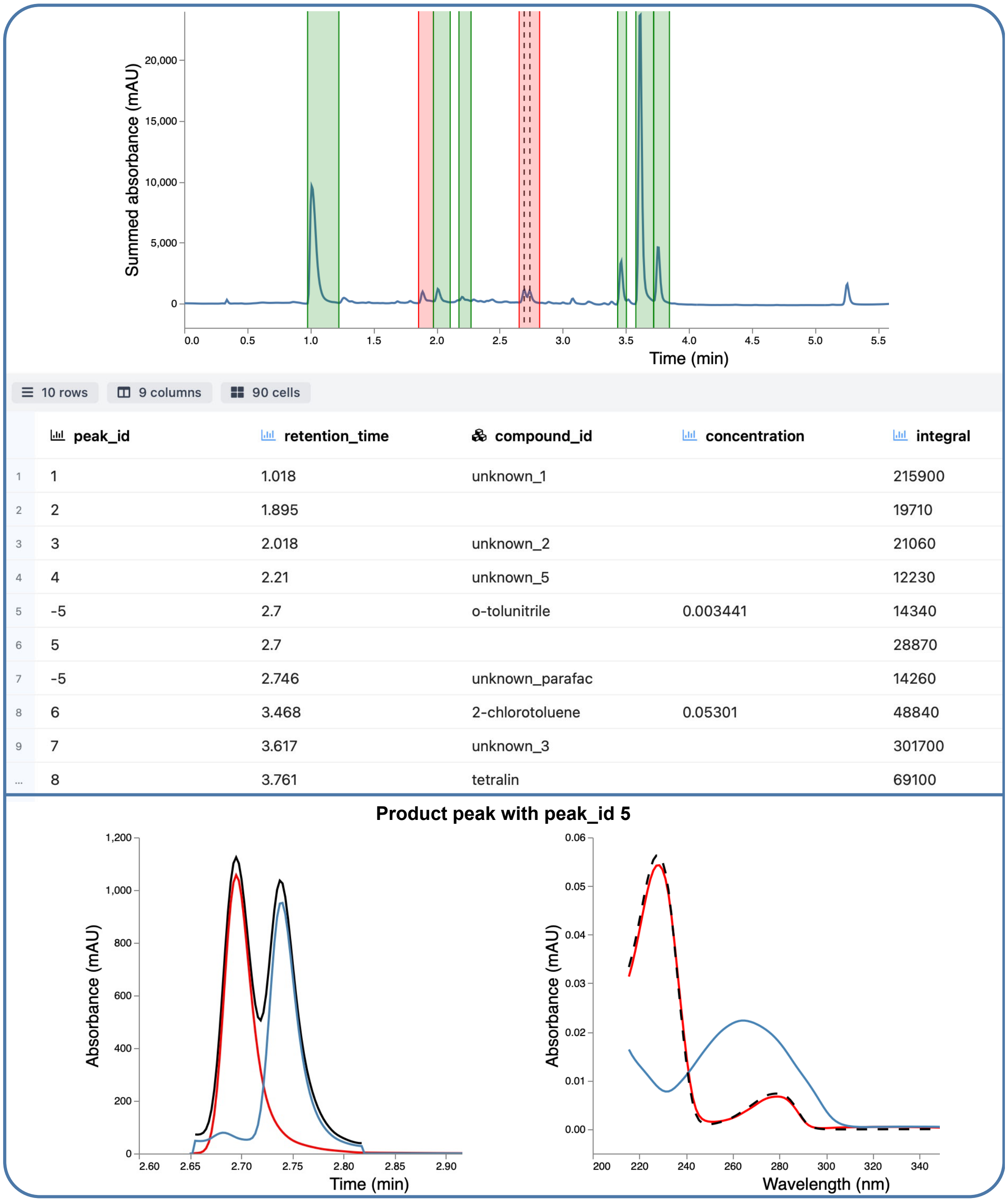

	peak_id	retention_time	compound_id	concentration	integral
1	1	1.018	unknown_1		215900
2	2	1.895			19710
3	3	2.018	unknown_2		21060
4	4	2.21	unknown_5		12230
5	-5	2.7	o-tolunitrile	0.003441	14340
6	5	2.7			28870
7	-5	2.746	unknown_parafac		14260
8	6	3.468	2-chlorotoluene	0.05301	48840
9	7	3.617	unknown_3		301700
...	8	3.761	tetralin		69100

Figure S20. Example of an unexpected impurity overlapping with the calibrated product peak. The top panel shows screenshots out of the MOCCA reports including a plotted chromatogram with picked peaks and indicated purity checks (*green*: passed, *red*: failed) as well as the peak table. Here, the impure peak is assigned to the peak_id 5 and the modelled peaks are assigned to the peak_ids −5 indicating that they are modelled peaks orginating from the peak with peak_id 5. The bottom panel shows the retention profiles and UV-Vis traces of the two modelled components (*red*: product *o*-tolunitrile, *blue*: unknown impurity).

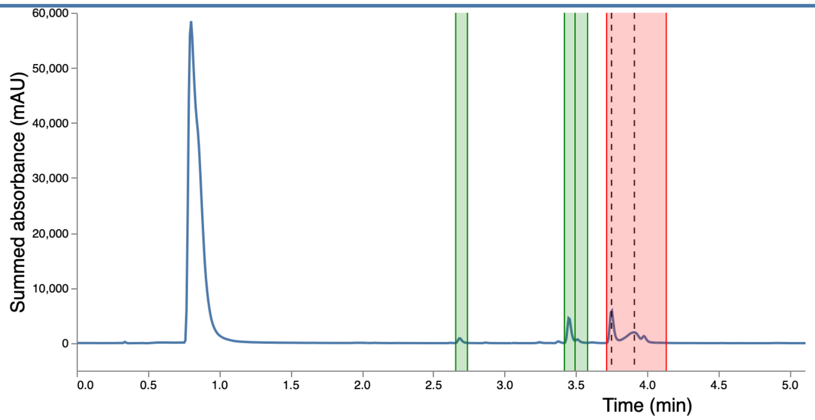

6 rows | 9 columns | 54 cells

	peak_id	retention_time	compound_id	concentration	integral
1	1	2.684	o-tolunitrile	0.001835	11930
2	2	3.452	2-chlorotoluene	0.0498	56560
3	3	3.511	unknown_36		10150
4	-4	3.751	tetralin		78430
5	4	3.751			174000
6	-4	3.911	unknown_parafac		96620

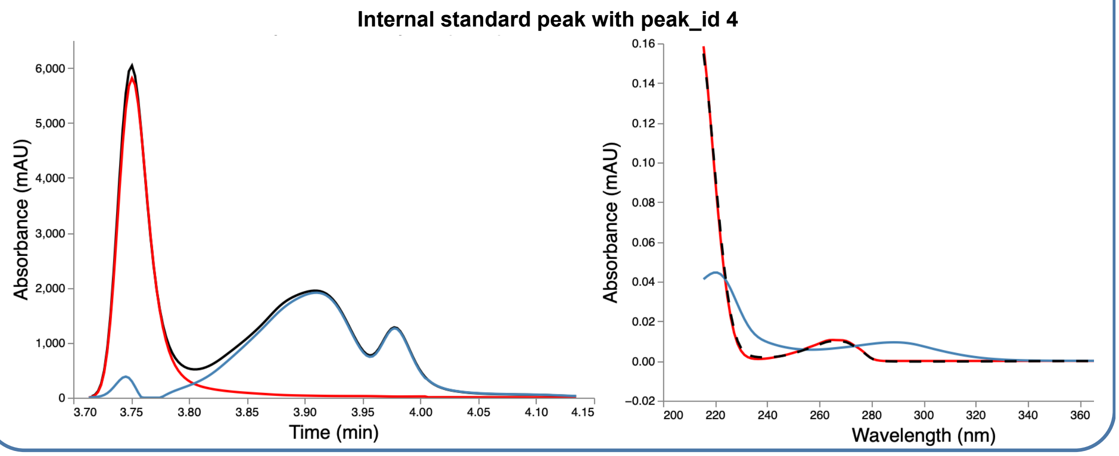

Figure S21. Example of an unexpected impurity overlapping with the internal standard peak. The top panel shows screenshots out of the MOCCA reports including a plotted chromatogram with picked peaks and indicated purity checks (*green*: passed, *red*: failed) as well as the peak table. Here, the impure peak is assigned to the peak_id 4 and the modelled peaks are assigned to the peak_ids −4 indicating that they are modelled peaks orginating from the peak with peak_id 4. The bottom panel shows the retention profiles and UV-Vis traces of the two modelled components (*red*: internal standard tetraline, *blue*: unknown impurity).

S10) NMR spectra *O*-protected cyanohydrins

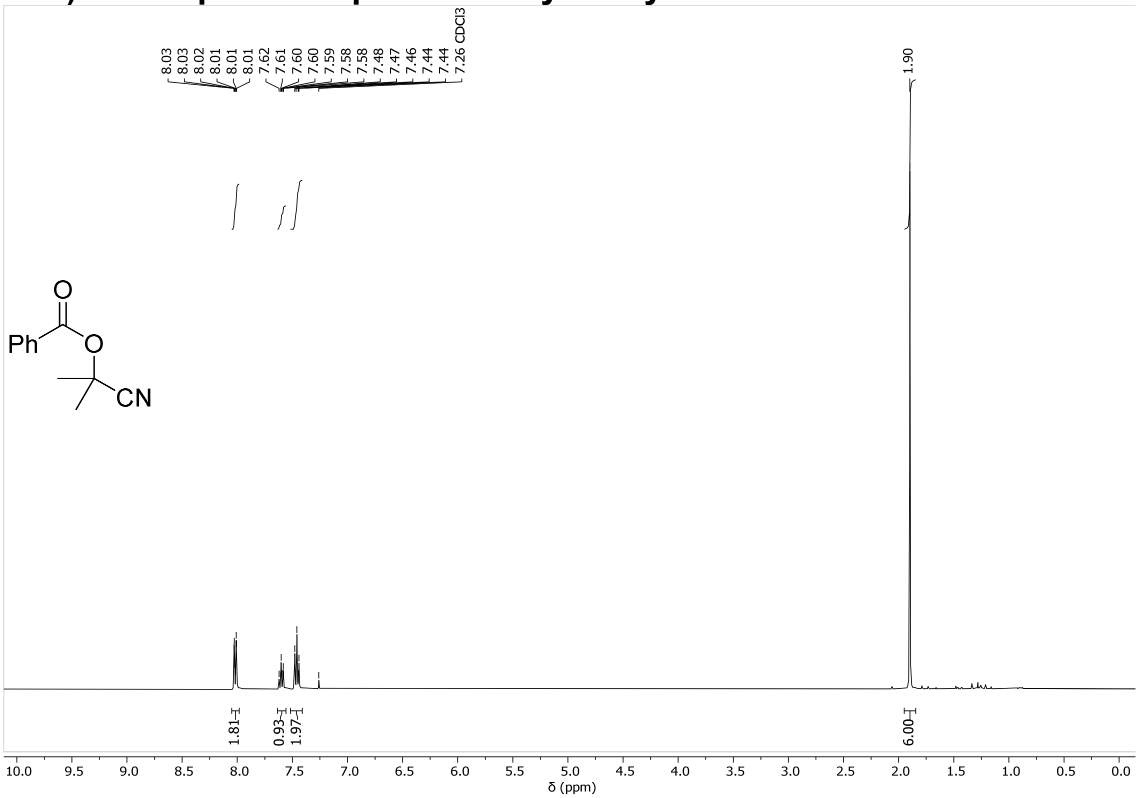

Figure S22. ^{1}H NMR spectrum of 2-Cyanopropan-2-yl benzoate (**10a**) in $CDCl_3$ recorded at 400 MHz.

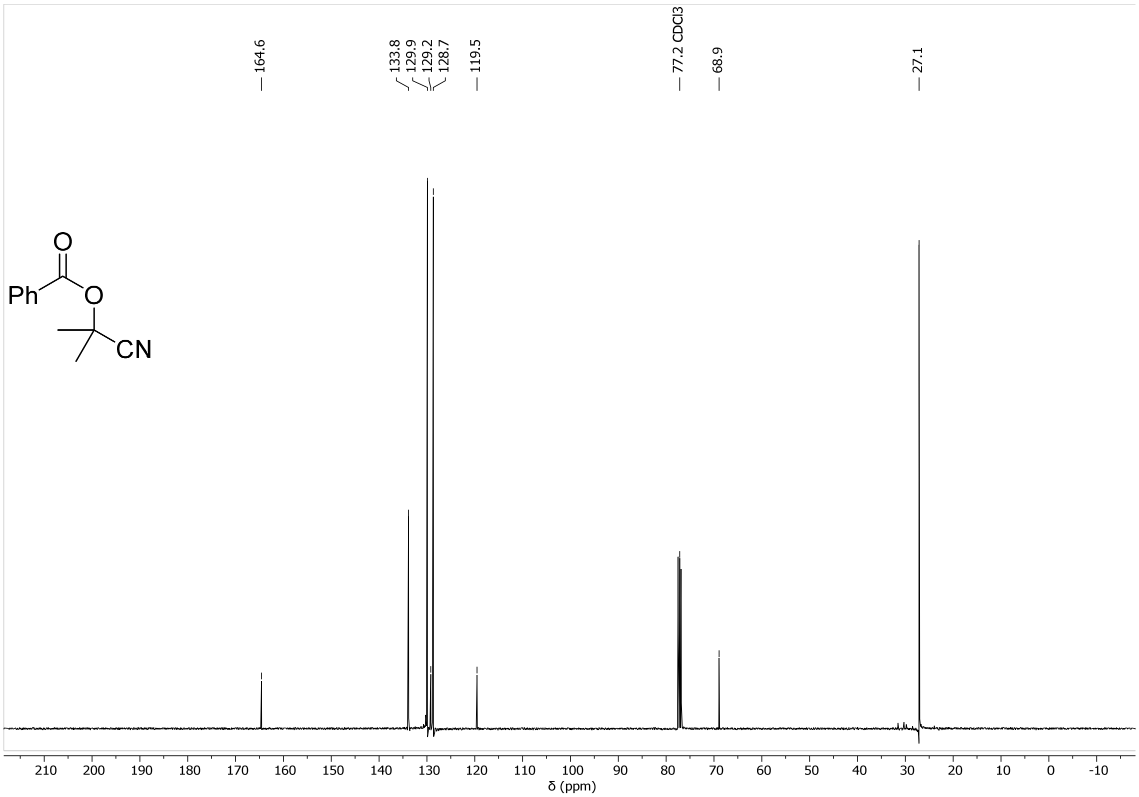

Figure S23. ^{13}C NMR spectrum of 2-Cyanopropan-2-yl benzoate (**10a**) in $CDCl_3$ recorded at 101 MHz.

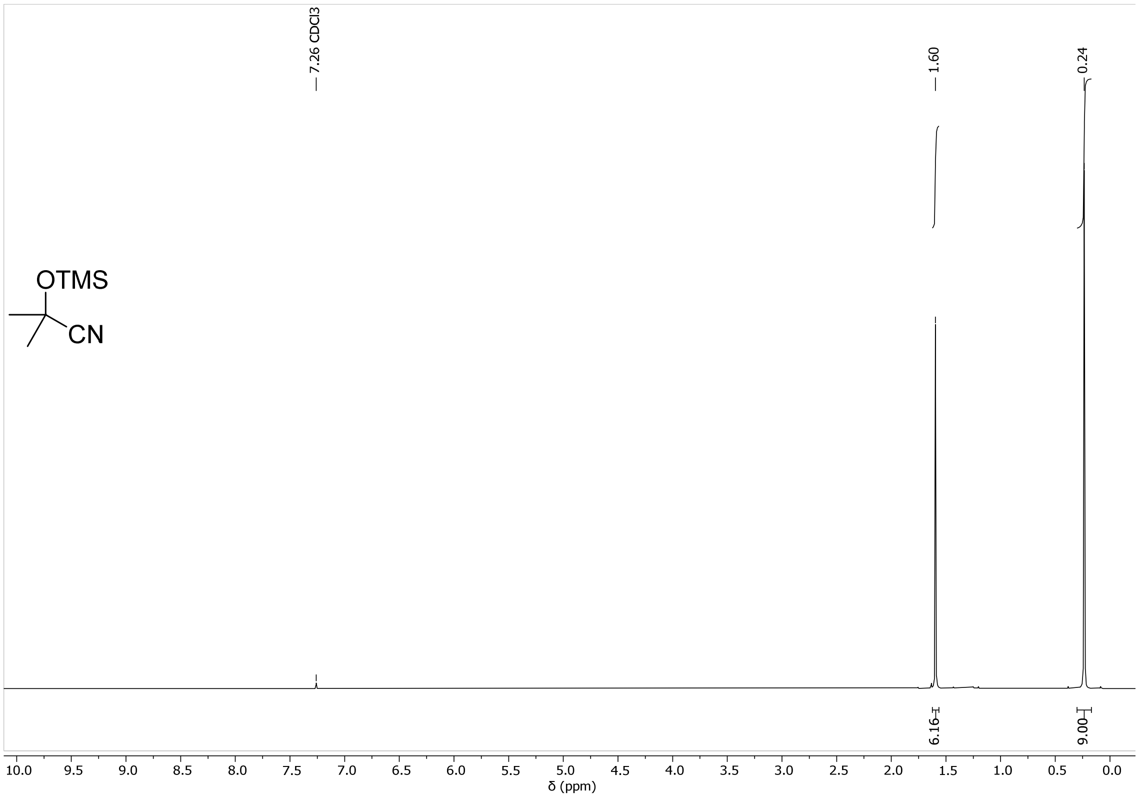

Figure S24. ^{1}H NMR spectrum of 2-Methyl-2-((trimethylsilyl)oxy)propanenitrile (**10b**) in $CDCl_3$ recorded at 400 MHz.

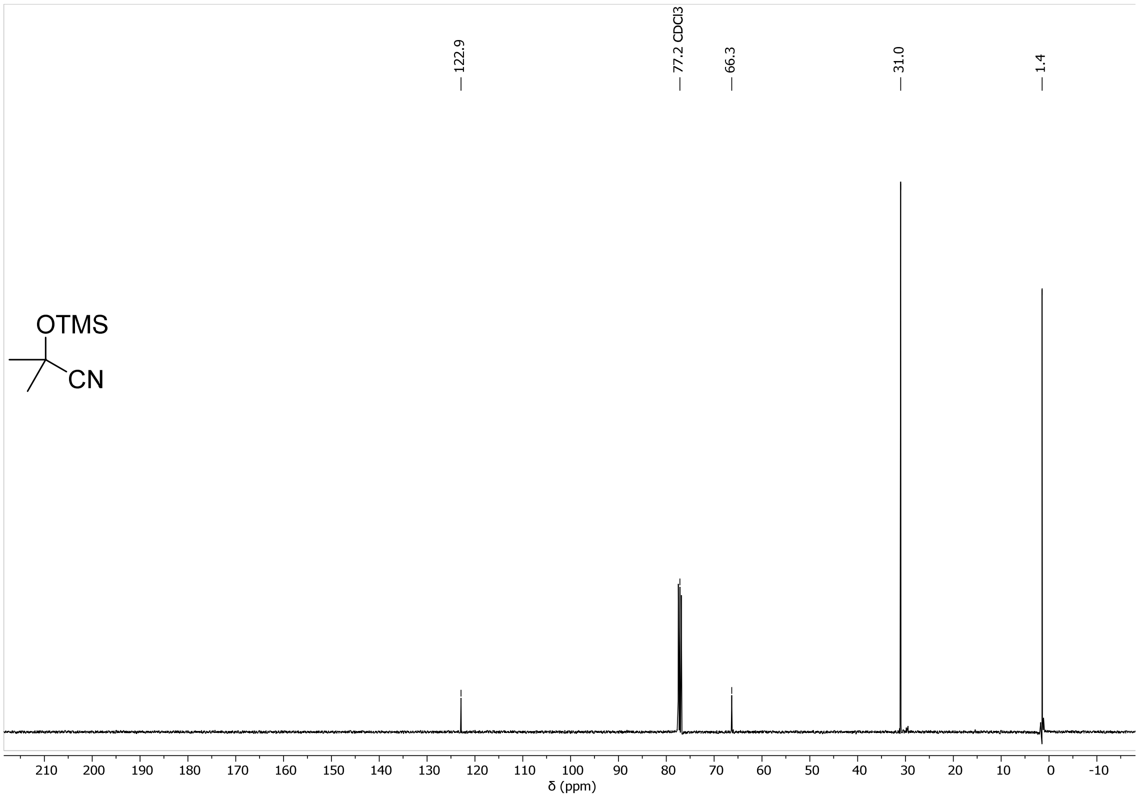

Figure S25. ^{13}C NMR spectrum of 2-Methyl-2-((trimethylsilyl)oxy)propanenitrile (**10b**) in $CDCl_3$ recorded at 101 MHz.

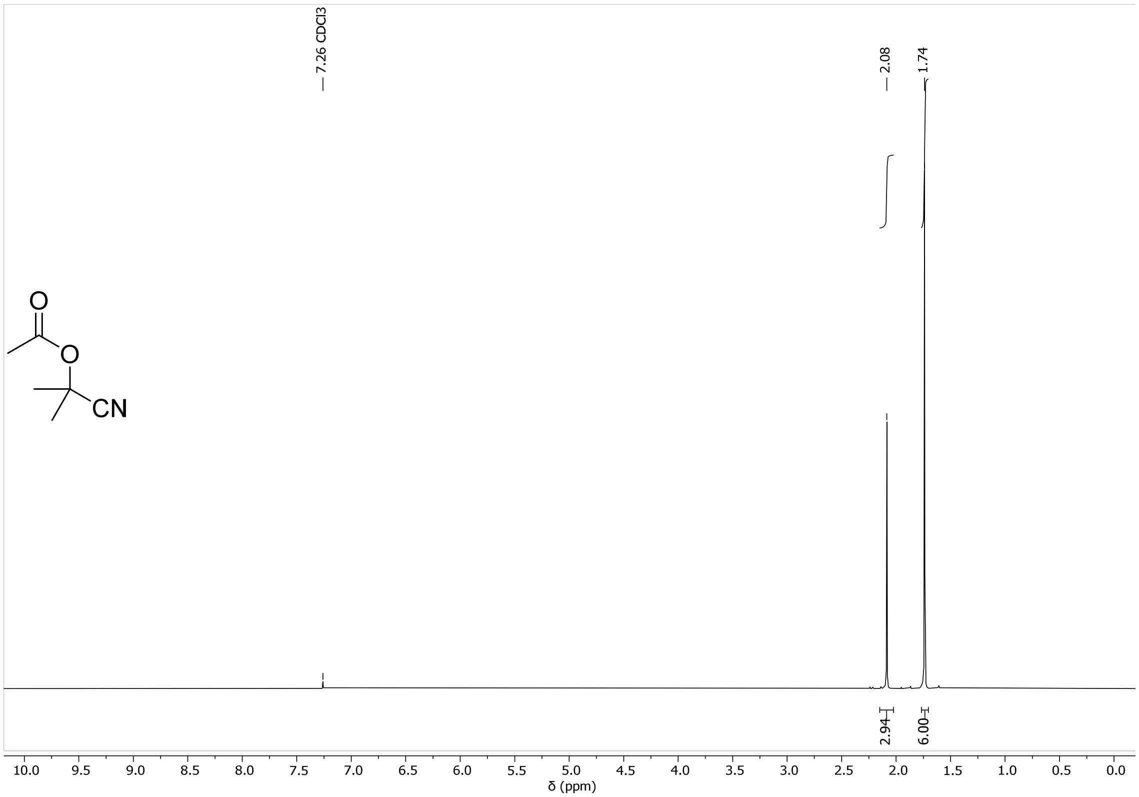

Figure S26. ^{1}H NMR spectrum of 2-Cyanopropan-2-yl acetate (**10c**) in $CDCl_3$ recorded at 500 MHz.

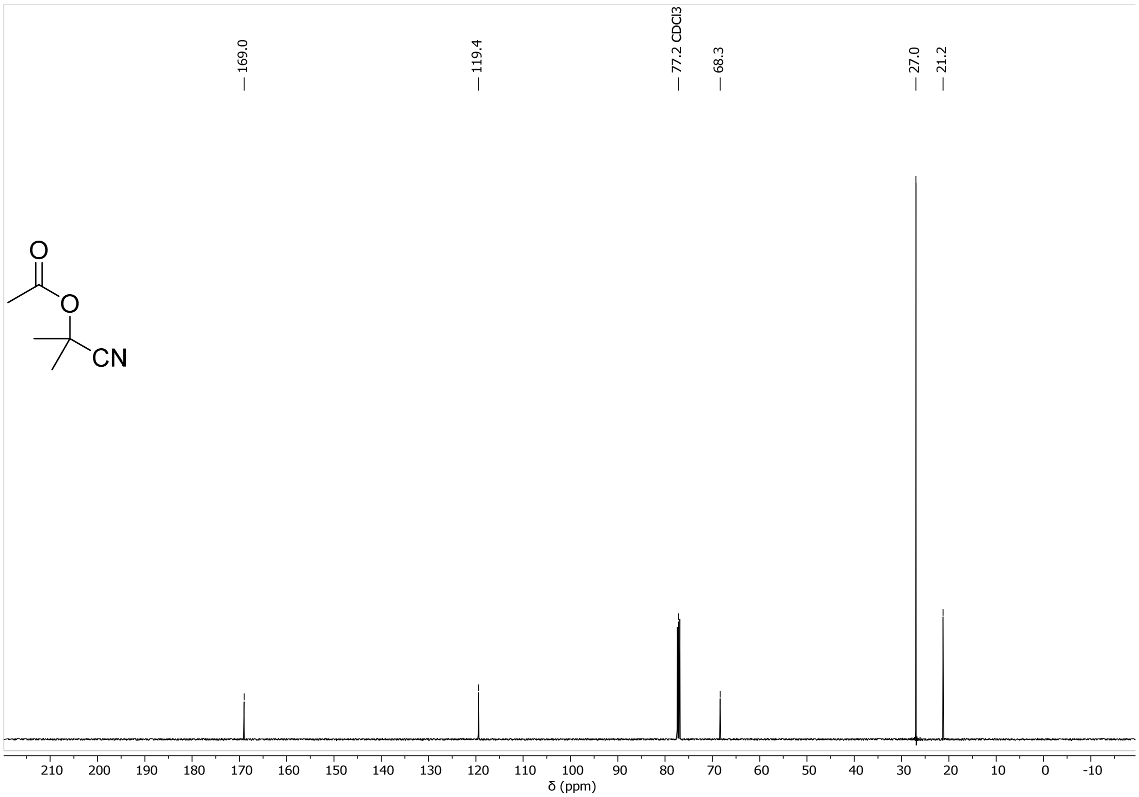

Figure S27. ^{13}C NMR spectrum of 2-Cyanopropan-2-yl acetate (**10c**) in $CDCl_3$ recorded at 126 MHz.

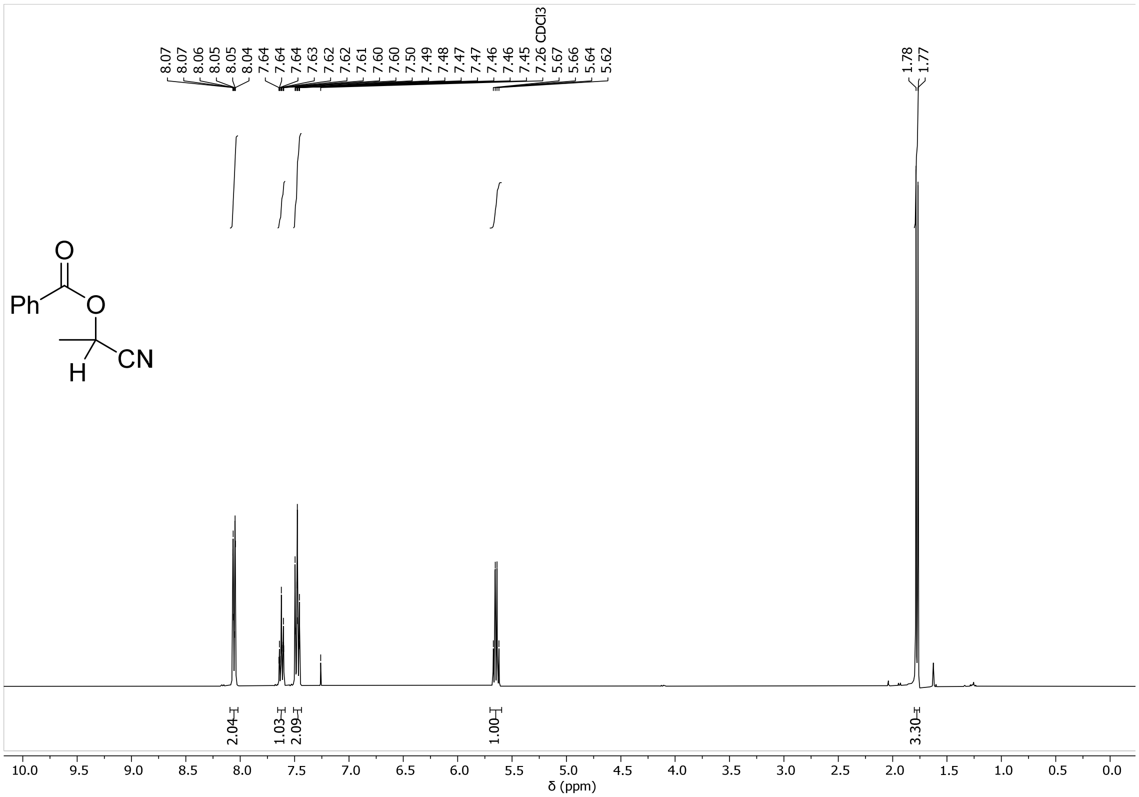

Figure S28. ^{1}H NMR spectrum of 1-Cyanoethyl benzoate (**10d**) in $CDCl_3$ recorded at 400 MHz.

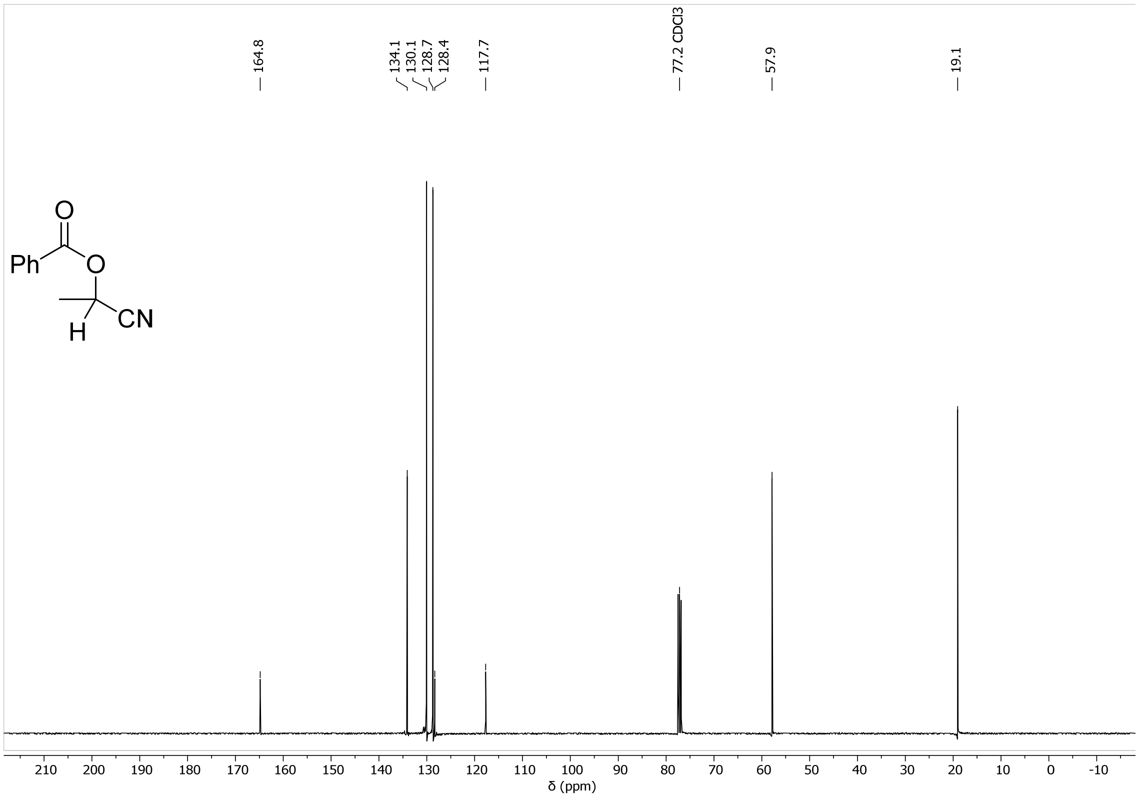

Figure S29. ^{13}C NMR spectrum of 1-Cyanoethyl benzoate (**10d**) in $CDCl_3$ recorded at 101 MHz.

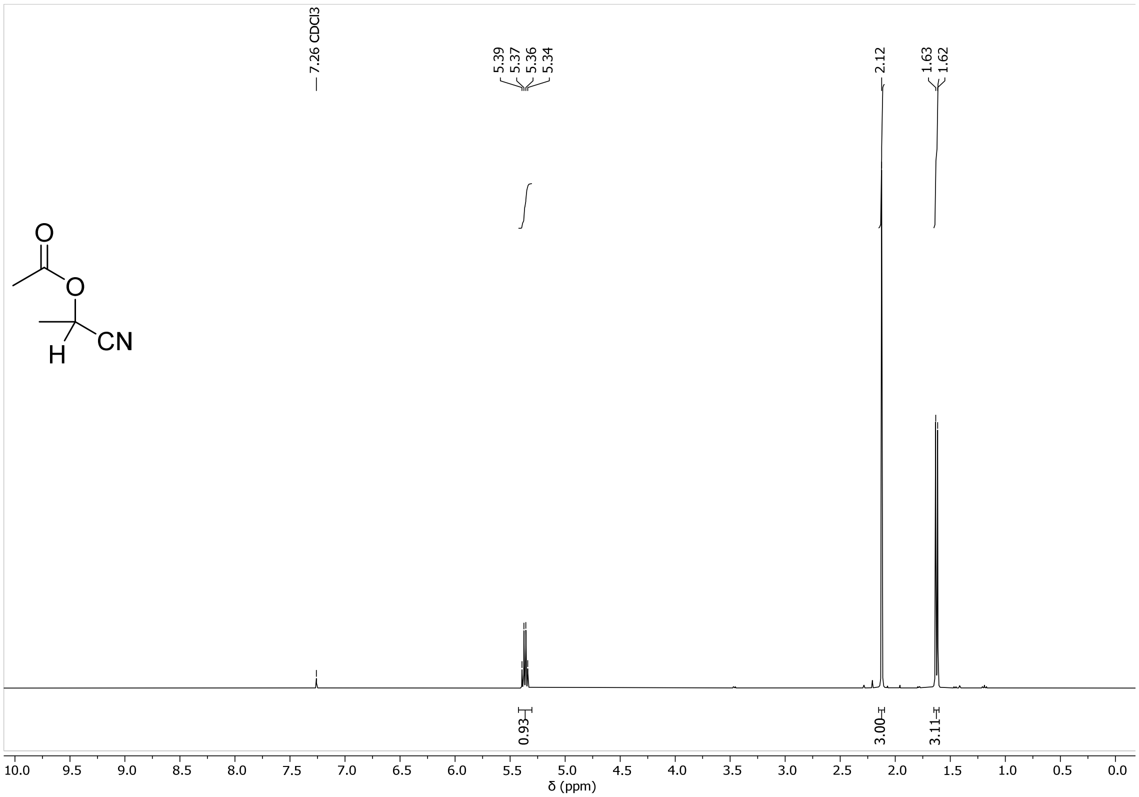

Figure S30. ^{1}H NMR spectrum of 1-Cyanoethyl acetate (**10e**) in $CDCl_3$ recorded at 400 MHz.

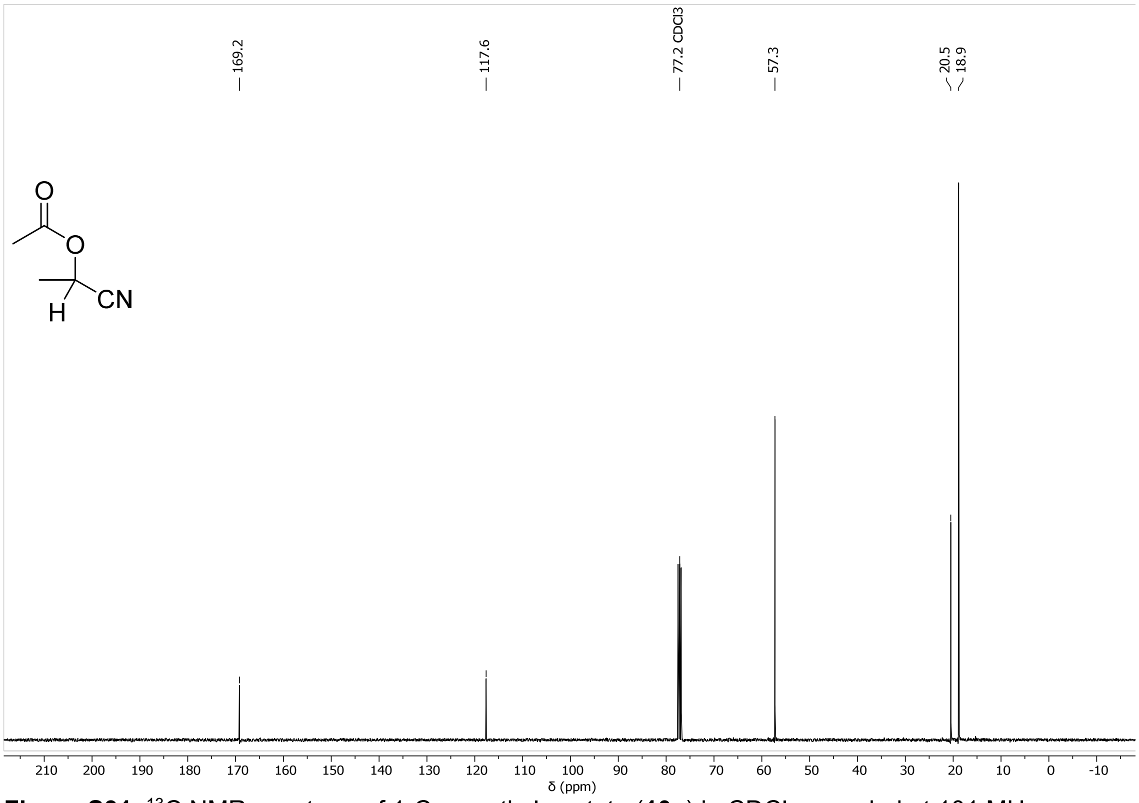

Figure S31. ^{13}C NMR spectrum of 1-Cyanoethyl acetate (**10e**) in $CDCl_3$ recorded at 101 MHz.

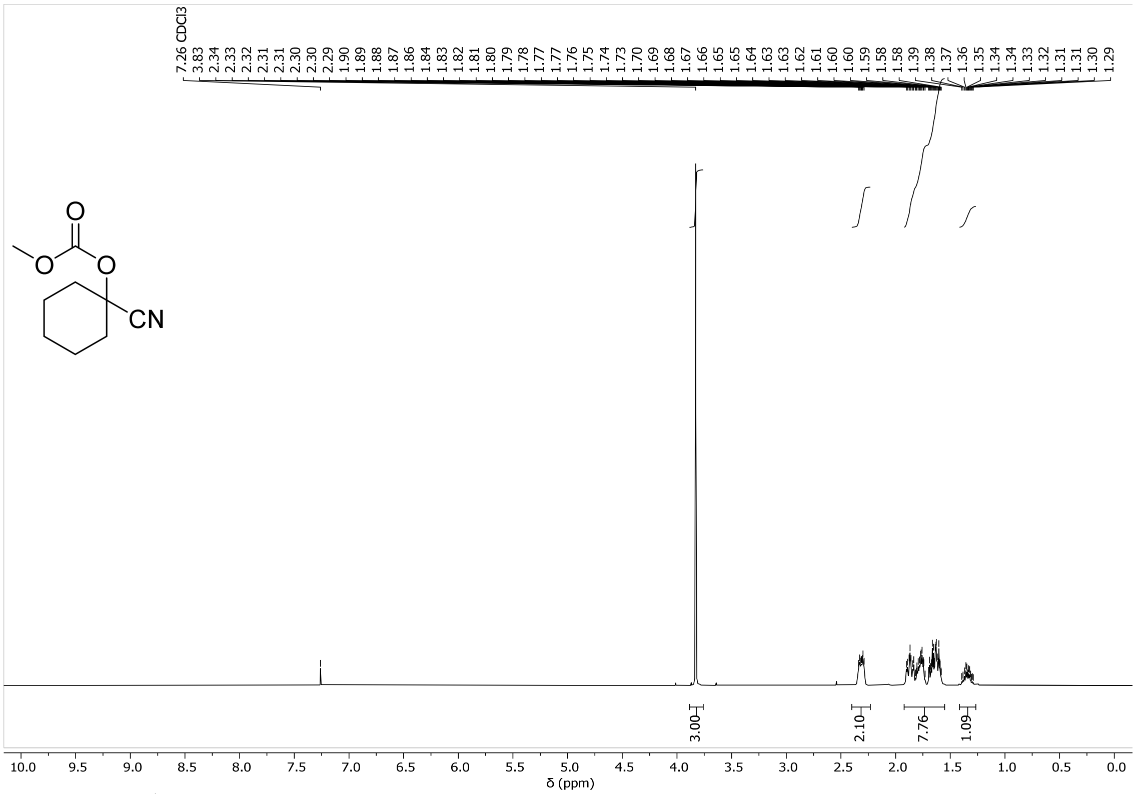

Figure S32. ^{1}H NMR spectrum of 1-Cyanocyclohexyl methyl carbonate (**10f**) in $CDCl_3$ recorded at 400 MHz.

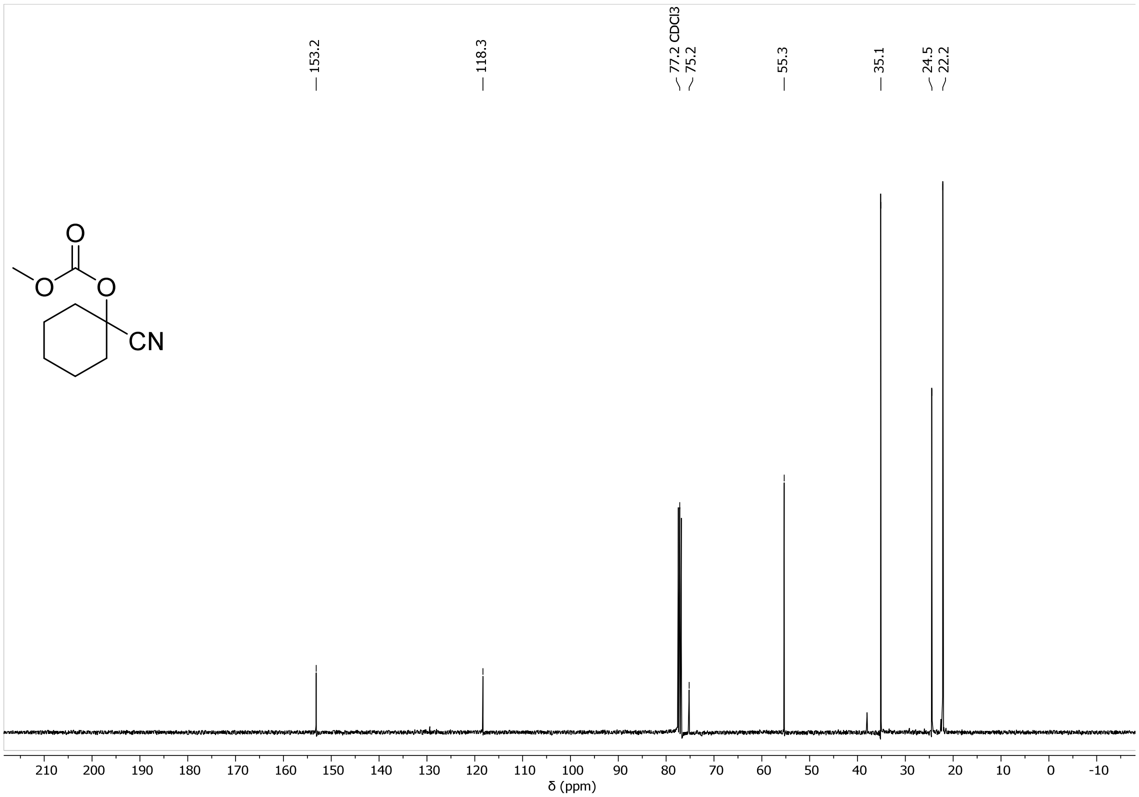

Figure S33. ^{13}C NMR spectrum of 1-Cyanocyclohexyl methyl carbonate (**10f**) in $CDCl_3$ recorded at 101 MHz.

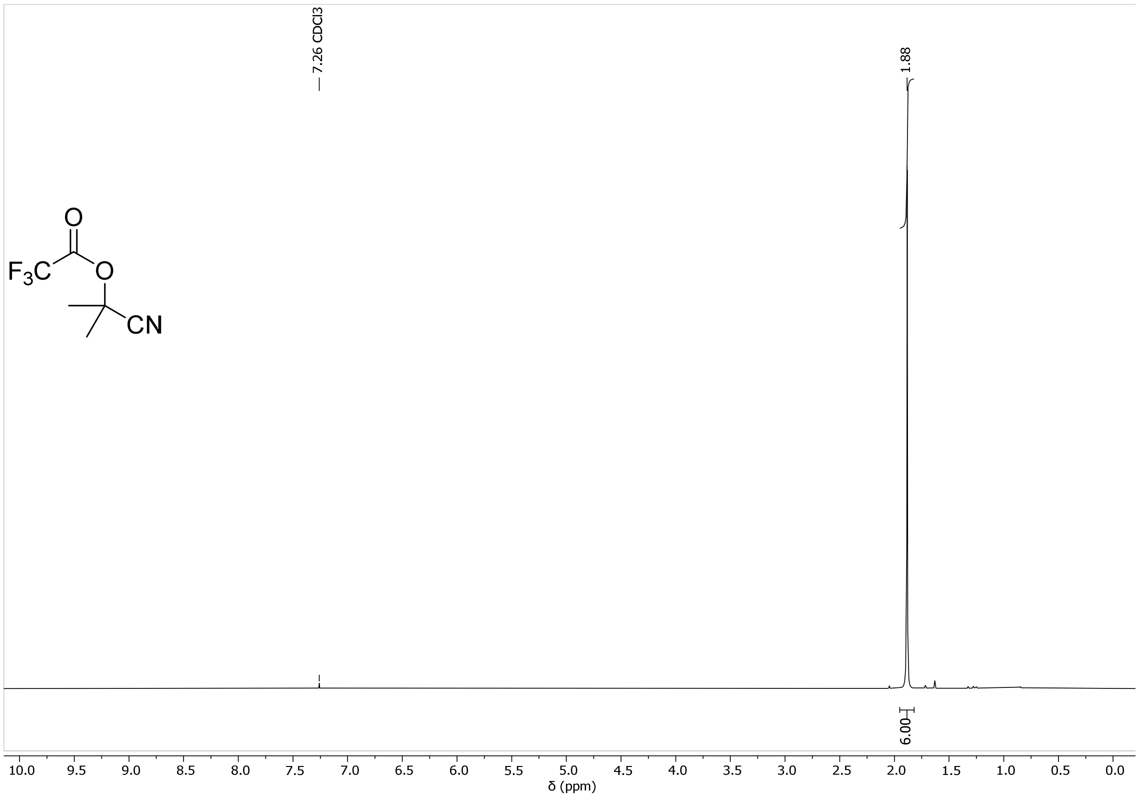

Figure S34. ^{1}H NMR spectrum of 2-Cyanopropan-2-yl 2,2,2-trifluoroacetate (**10g**) in $CDCl_3$ recorded at 400 MHz.

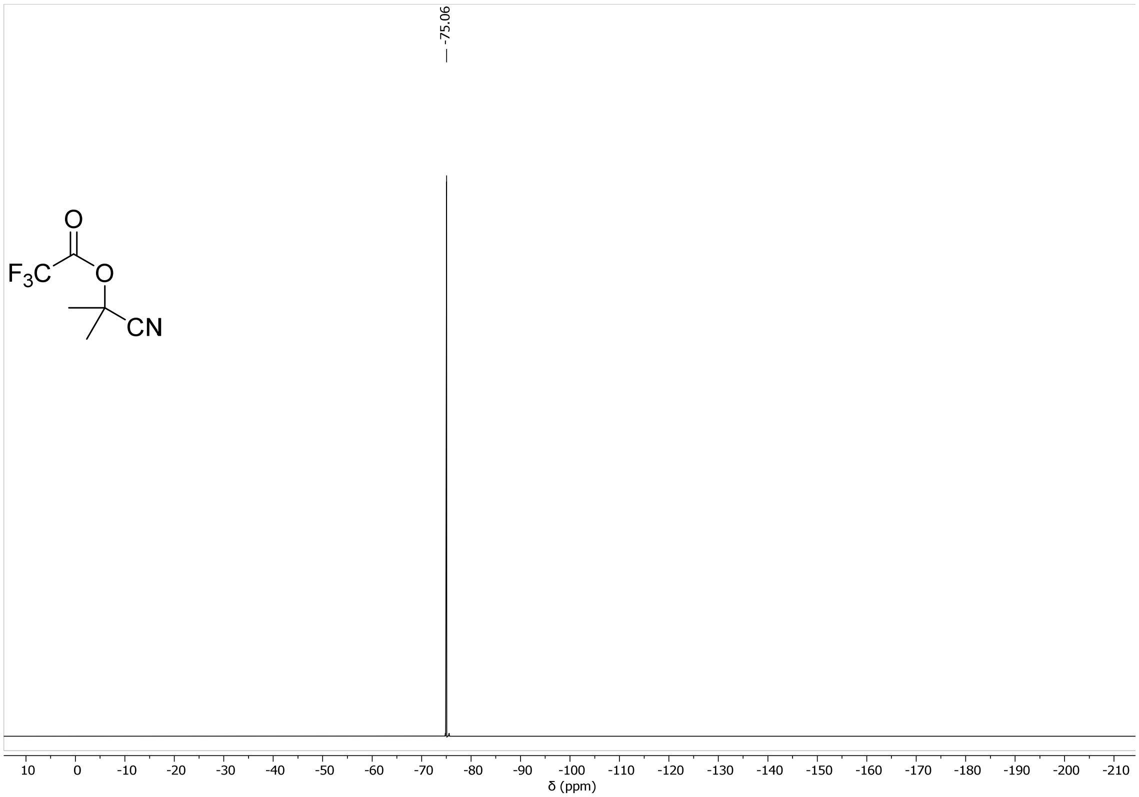

Figure S35. ^{19}F NMR spectrum of 2-Cyanopropan-2-yl 2,2,2-trifluoroacetate (**10g**) in $CDCl_3$ recorded at 376 MHz.

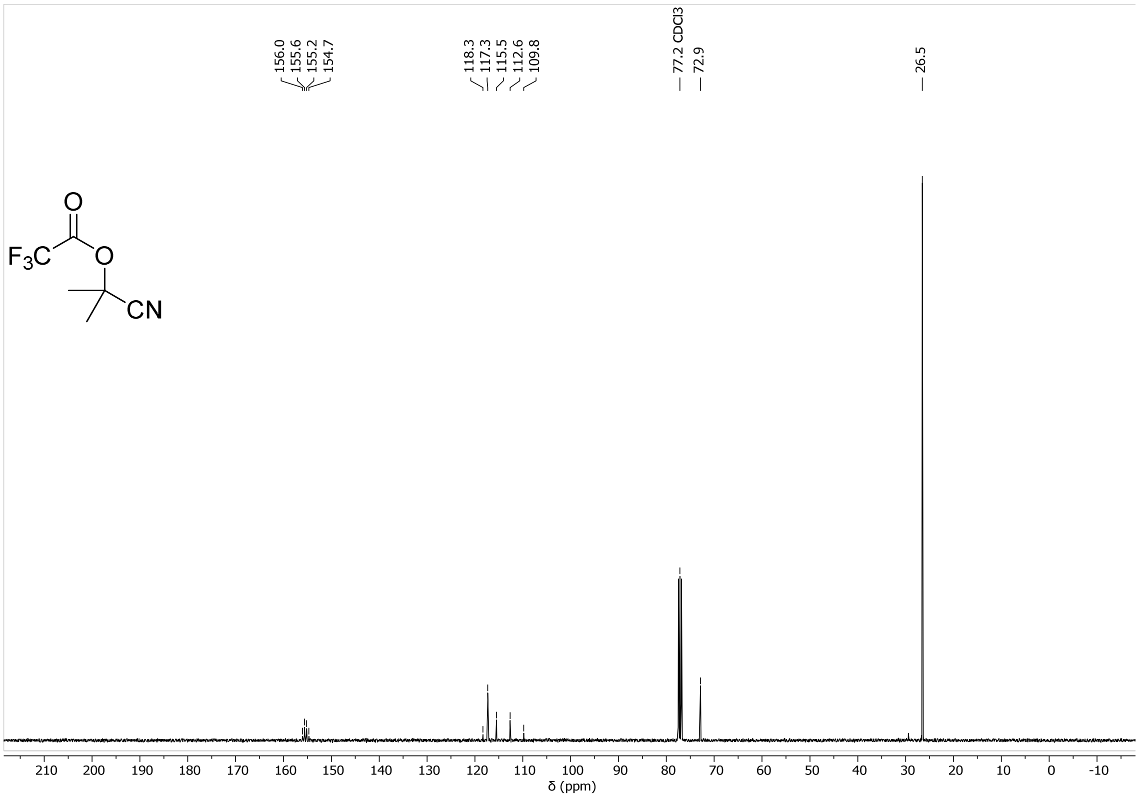

Figure S36. ^{13}C NMR spectrum of 2-Cyanopropan-2-yl 2,2,2-trifluoroacetate (**10g**) in $CDCl_3$ recorded at 101 MHz.

References

(1) *Allotrope Foundation, accessed May 2022*. https://www.allotrope.org.

(2) Bovee, R. *Entab, accessed May 2022*. https://github.com/bovee/entab.

(3) Hewlett-Packard Company. *Macro Programming Guide, accessed May 2022*. https://www.agilent.com/cs/library/usermanuals/Public/MACROS.PDF.

(4) *Waters Knowledge Base, accessed May 2022*. https://support.waters.com/KB_Inf/Empower_Breeze/WKB9865_How_do_you_export_raw_data_from_Empower_to_a_Microsoft_Excel_spreadsheet.

(5) Millecam, T.; Jarrett, A. J.; Young, N.; Vanderwall, D. E.; Della Corte, D. Coming of Age of Allotrope: Proceedings from the Fall 2020 Allotrope Connect. *Drug Discov. Today* **2021**, *26* (8), 1922–1928. https://doi.org/10.1016/j.drudis.2021.03.028.

(6) Koranne, S. *Handbook of Open Source Tools*; 2011. https://doi.org/10.1007/978-1-4419-7719-9.

(7) Horsch, M. T. *Data Technology in Materials Modelling*; 2021.

(8) Agilent Technologies. *Creating an Allotrope file format, accessed May 2022*. https://www.agilent.com/cs/library/technicaloverviews/public/technicaloverview-allotrope-data-format-openlab-cds-5994-0886en-us-agilent.pdf.

(9) Agilent Technologies. *Archiving Waters Empower Data in the Allotrope File Format, accessed August 2022*. https://lcms.cz/labrulez-bucket-strapi-h3hsga3/te_allotrope_waters_data_openlab_ecm_5994_4790en_us_agilent_798e686682/te-allotrope-waters-data-openlab-ecm-5994-4790en-us-agilent.pdf.

(10) Waters Corporation. *Waters press release, accessed August 2022*. https://ir.waters.com/newsroom/press-release-details/2021/Waters-and-TetraScience-Partner-to-Deliver-New-Levels-of-Data-Access-and-Insights-with-Empower-Data-Science-Link/default.aspx.

(11) Thermo Fisher Scientific Inc. *Chromeleon release notes version 7.2.10, accessed August 2022*. https://freshwebmaterial.s3-us-west-2.amazonaws.com/Software/Chromeleon/Documentation/Chremeleon+7.2.10+Documentation/Release+Notes+-+Chromeleon+7.2.10.pdf.

(12) *h5py GitHub repository, accessed August 2022*. https://github.com/h5py/h5py.

(13) Agilent Technologies. *Best Practices for Efficient Liquid Chromatography (LC) Operations, accessed May 2022*.

https://www.agilent.com/cs/library/primers/public/Best_Practice_LC_Operations.pdf.

(14) Agilent Technologies. *Diode Array Detector Optimization, accessed May 2022*. https://www.agilent.com/cs/library/slidepresentation/Public/2 Diode Array Detector Optimization.pdf.

(15) Eilers, P. H.; Boelens, H. F. Baseline Correction with Asymmetric Least Squares Smoothing. *Leiden Univ. Med. Cent. Rep.* **2005**.

(16) Guliev, R. *Optimized algorithm, accessed July 2022*. https://stackoverflow.com/questions/29156532/python-baseline-correction-library.

(17) Virtanen, P.; Gommers, R.; Oliphant, T. E.; Haberland, M.; Reddy, T.; Cournapeau, D.; Burovski, E.; Peterson, P.; Weckesser, W.; Bright, J.; van der Walt, S. J.; Brett, M.; Wilson, J.; Millman, K. J.; Mayorov, N.; Nelson, A. R. J.; Jones, E.; Kern, R.; Larson, E.; Carey, C. J.; Polat, İ.; Feng, Y.; Moore, E. W.; VanderPlas, J.; Laxalde, D.; Perktold, J.; Cimrman, R.; Henriksen, I.; Quintero, E. A.; Harris, C. R.; Archibald, A. M.; Ribeiro, A. H.; Pedregosa, F.; van Mulbregt, P.; Vijaykumar, A.; Bardelli, A. Pietro; Rothberg, A.; Hilboll, A.; Kloeckner, A.; Scopatz, A.; Lee, A.; Rokem, A.; Woods, C. N.; Fulton, C.; Masson, C.; Häggström, C.; Fitzgerald, C.; Nicholson, D. A.; Hagen, D. R.; Pasechnik, D. V.; Olivetti, E.; Martin, E.; Wieser, E.; Silva, F.; Lenders, F.; Wilhelm, F.; Young, G.; Price, G. A.; Ingold, G. L.; Allen, G. E.; Lee, G. R.; Audren, H.; Probst, I.; Dietrich, J. P.; Silterra, J.; Webber, J. T.; Slavič, J.; Nothman, J.; Buchner, J.; Kulick, J.; Schönberger, J. L.; de Miranda Cardoso, J. V.; Reimer, J.; Harrington, J.; Rodríguez, J. L. C.; Nunez-Iglesias, J.; Kuczynski, J.; Tritz, K.; Thoma, M.; Newville, M.; Kümmerer, M.; Bolingbroke, M.; Tartre, M.; Pak, M.; Smith, N. J.; Nowaczyk, N.; Shebanov, N.; Pavlyk, O.; Brodtkorb, P. A.; Lee, P.; McGibbon, R. T.; Feldbauer, R.; Lewis, S.; Tygier, S.; Sievert, S.; Vigna, S.; Peterson, S.; More, S.; Pudlik, T.; Oshima, T.; Pingel, T. J.; Robitaille, T. P.; Spura, T.; Jones, T. R.; Cera, T.; Leslie, T.; Zito, T.; Krauss, T.; Upadhyay, U.; Halchenko, Y. O.; Vázquez-Baeza, Y. SciPy 1.0: Fundamental Algorithms for Scientific Computing in Python. *Nat. Methods* **2020**, *17* (3), 261–272. https://doi.org/10.1038/s41592-019-0686-2.

(18) Stahl, M. *Peak purity analysis in HPLC and CE using diode-array technology, accessed August 2022*. https://www.agilent.com/cs/library/applications/5988-

8647EN.pdf.
(19) Waters Corporation. *PDA Software Getting Started Guide, accessed August 2022*. https://www.waters.com/webassets/cms/support/docs/wat053020tpr1.pdf.
(20) Olivieri, A. C.; Escandar, G. M. *Practical Three-Way Calibration*; 2000.
(21) Bro, R. PARAFAC. Tutorial and Applications. *Chemom. Intell. Lab. Syst.* **1997**, *38* (2), 149–171. https://doi.org/10.1016/S0169-7439(97)00032-4.
(22) O’Hanlon, J. A.; Chapman, R. D.; Taylor, F.; Denecke, M. A. Quantification of Common Aminopolycarboxylic Acids in Trench Leachate from the Low Level Waste Repository. *J. Radioanal. Nucl. Chem.* **2019**, *322* (3), 1915–1929. https://doi.org/10.1007/s10967-019-06895-x.
(23) Escandar, G. M.; Olivieri, A. C. Multi-Way Chromatographic Calibration — A Review. *J. Chromatogr. A* **2019**, *1587*, 2–13. https://doi.org/10.1016/j.chroma.2019.01.012.
(24) Tauler, R. Multivariate Curve Resolution of Multiway Data Using the Multilinearity Constraint. *J. Chemom.* **2020**, No. June 2020, 1–24. https://doi.org/10.1002/cem.3279.
(25) Zhang, J.; Guo, C.; Cai, W.; Shao, X. Direct Non-Trilinear Decomposition for Analyzing High-Dimensional Data with Imperfect Trilinearity. *Chemom. Intell. Lab. Syst.* **2021**, *210* (January), 104244. https://doi.org/10.1016/j.chemolab.2021.104244.
(26) Kossaifi, J.; Panagakis, Y.; Anandkumar, A.; Pantic, M. TensorLy: Tensor Learning in Python. *J. Mach. Learn. Res.* **2019**, *20*, 1–6.
(27) Haas, C. P. *MOCCA GitHub repository*. 2022. https://github.com/HaasCP/mocca.
(28) *Datapane, accessed August 2022*. https://github.com/datapane/datapane.
(29) Leweke, S.; von Lieres, E. Chromatography Analysis and Design Toolkit (CADET). *Comput. Chem. Eng.* **2018**, *113*, 274–294. https://doi.org/10.1016/j.compchemeng.2018.02.025.
(30) Baumgartner, L. M.; Coley, C. W.; Reizman, B. J.; Gao, K. W.; Jensen, K. F. Optimum Catalyst Selection over Continuous and Discrete Process Variables with a Single Droplet Microfluidic Reaction Platform. *React. Chem. Eng. Eng.* **2018**, *3* (3), 301–311. https://doi.org/10.1039/C8RE00032H.
(31) Shields, B. J.; Stevens, J.; Li, J.; Parasram, M.; Damani, F.; Alvarado, J. I. M.;

Janey, J. M.; Adams, R. P.; Doyle, A. G. Bayesian Reaction Optimization as a Tool for Chemical Synthesis. *Nature* **2021**, *590* (7844), 89–96. https://doi.org/10.1038/s41586-021-03213-y.

(32) Sieburth, S. M. N.; Lin, C. H.; Rucando, D. Selective Intermolecular Photo-[4 + 4]-Cycloaddition with 2-Pyridone Mixtures. 2. Preparation of (1α,2β,5β,6α)-3-Butyl-9-Methoxy-3,7- Diazatricyclo[4.2.2.22,5]Dodeca-9,11-Diene-4,8-Dione. *J. Org. Chem.* **1999**, *64* (3), 950–953. https://doi.org/10.1021/jo981932c.

(33) Lethesh, K. C.; Shah, S. N.; Mutalib, M. I. A. Synthesis, Characterization, and Thermophysical Properties of 1,8- Diazobicyclo[5.4.0]Undec-7-Ene Based Thiocyanate Ionic Liquids. *J. Chem. Eng. Data* **2014**, *59*, 1788–1795.

(34) Zhang, W.; Shi, M. DBU Catalyzed Cyanoacylation of Ketones with Acyl Cyanides. *Org. Biomol. Chem.* **2006**, *4* (9), 1671–1674. https://doi.org/10.1039/b602197b.

(35) Kraft, P.; Popaj, K.; Abate, A. Design, Synthesis and Olfactory Properties of 2-Substituted 2-Tert-Butyl-5-Methyl-2,5-Dihydrofurans: Seco-Derivatives of Theaspiranes. *Synthesis (Stuttg).* **2005**, No. 16, 2798–2809. https://doi.org/10.1055/s-2005-918404.

(36) Sharma, M. K.; Sinhababu, S.; Mukherjee, G.; Rajaraman, G.; Nagendran, S. A Cationic Aluminium Complex: An Efficient Mononuclear Main-Group Catalyst for the Cyanosilylation of Carbonyl Compounds. *Dalt. Trans.* **2017**, *46* (24), 7672–7676. https://doi.org/10.1039/c7dt01760j.

(37) Hiyama, T.; Oishi, H.; Suetsugu, Y. Synthesis of 4-Amino-2(5H)-Furanones through Intra- and Intermolecular Nitrile Addition of Ester Enolates. Construction of Carbon Framework of an Antitumor Antibiotic Basidalin. *Bull. Chem. Soc. Jpn.* **1987**, *60* (6), 2139–2150. https://doi.org/10.1246/bcsj.60.2139.

(38) Steemers, L.; Wijsman, L.; van Maarseveen, J. H. Regio- and Stereoselective Chan-Lam-Evans Enol Esterification of Carboxylic Acids with Alkenylboroxines. *Adv. Synth. Catal.* **2018**, *360* (21), 4241–4245. https://doi.org/10.1002/adsc.201800914.

(39) Matsukawa, S.; Kimura, J.; Yoshioka, M. TBD- or PS-TBD-Catalyzed One-Pot Synthesis of Cyanohydrin Carbonates and Cyanohydrin Acetates from Carbonyl Compounds. *Molecules* **2016**, *21* (8), 1030. https://doi.org/10.3390/molecules21081030.
