# Supplementary material for: Open-Source Chromatographic Data Analysis for Reaction Optimization and Screening
Source: ACS Cent Sci. 2023 Feb 9;9(2):307–17. doi: 10.1021/acscentsci.2c01042 (PMC9951288; doi:10.1021/acscentsci.2c01042)
Supplement: Supplementary file 2 — oc2c01042_si_002.zip [file oc2c01042_si_002.zip › cyanation_reports/compound_library.html]

compound\_library created on
